# Cell cycle, oncogenic and tumor suppressor pathways regulate numerous long and macro non-protein-coding RNAs

**DOI:** 10.1186/gb-2014-15-3-r48

**Published:** 2014-03-04

**Authors:** Jörg Hackermüller, Kristin Reiche, Christian Otto, Nadine Hösler, Conny Blumert, Katja Brocke-Heidrich, Levin Böhlig, Anne Nitsche, Katharina Kasack, Peter Ahnert, Wolfgang Krupp, Kurt Engeland, Peter F Stadler, Friedemann Horn

**Affiliations:** 1 Young Investigators Group Bioinformatics and Transcriptomics, Department Proteomics, Helmholtz Centre for Environmental Research – UFZ, Leipzig, Germany; 2 Department for Computer Science, University of Leipzig, Leipzig, Germany; 3 RNomics Group, Department of Diagnostics, Fraunhofer Institute for Cell Therapy and Immunology – IZI, Leipzig, Germany; 4 Bioinformatics Group, Department of Computer Science, University of Leipzig, Leipzig, Germany; 5 LIFE - Leipzig Research Center for Civilization Diseases, University of Leipzig, Leipzig, Germany; 6 Institute of Clinical Immunology, University of Leipzig, Leipzig, Germany; 7 Department of Diagnostics, Fraunhofer Institute for Cell Therapy and Immunology – IZI, Leipzig, Germany; 8 Molecular Oncology, Medical School, University of Leipzig, Leipzig, Germany; 9 Institute for Medical Informatics, Statistics and Epidemiology, University of Leipzig, Leipzig, Germany; 10 Clinic of Neurosurgery, University of Leipzig, Leipzig, Germany; 11 Max-Planck Institute for Mathematics in the Sciences, Leipzig, Germany; 12 Santa Fe Institute, Santa Fe, NM, USA; 13 Department of Theoretical Chemistry, University of Vienna, Vienna, Austria

## Abstract

**Background:**

The genome is pervasively transcribed but most transcripts do not code for proteins, constituting non-protein-coding RNAs. Despite increasing numbers of functional reports of individual long non-coding RNAs (lncRNAs), assessing the extent of functionality among the non-coding transcriptional output of mammalian cells remains intricate. In the protein-coding world, transcripts differentially expressed in the context of processes essential for the survival of multicellular organisms have been instrumental in the discovery of functionally relevant proteins and their deregulation is frequently associated with diseases. We therefore systematically identified lncRNAs expressed differentially in response to oncologically relevant processes and cell-cycle, p53 and STAT3 pathways, using tiling arrays.

**Results:**

We found that up to 80% of the pathway-triggered transcriptional responses are non-coding. Among these we identified very large macroRNAs with pathway-specific expression patterns and demonstrated that these are likely continuous transcripts. MacroRNAs contain elements conserved in mammals and sauropsids, which in part exhibit conserved RNA secondary structure. Comparing evolutionary rates of a macroRNA to adjacent protein-coding genes suggests a local action of the transcript. Finally, in different grades of astrocytoma, a tumor disease unrelated to the initially used cell lines, macroRNAs are differentially expressed.

**Conclusions:**

It has been shown previously that the majority of expressed non-ribosomal transcripts are non-coding. We now conclude that differential expression triggered by signaling pathways gives rise to a similar abundance of non-coding content. It is thus unlikely that the prevalence of non-coding transcripts in the cell is a trivial consequence of leaky or random transcription events.

## Background

Only a minor portion (1.5% to 2%) of mammalian genomic sequences code for proteins. Over the last decade, transcriptomics has shown that the majority of sequences in mammalian genomes are pervasively transcribed into RNA molecules [1-6], an overwhelming fraction of which is not translated [7]. Despite some dissenting opinions that questioned the number of novel intergenic transcripts [8] and hypothesized that there was a high potential for these transcripts to contain short open-reading frames [9], the concept of pervasive non-protein-coding transcription [10] is increasingly being accepted as a fact. Mammalian cells are thus capable of producing a plethora of non-protein-coding RNAs (ncRNAs). ncRNAs have been categorized rather superficially into long ncRNAs (lncRNAs), which are longer than 150 or 200 nt, and short ncRNAs. Most short ncRNAs fall into well-defined classes, such as microRNAs, piRNAs (piwi-interacting RNA), tRNAs (transfer RNAs), etc., for which there is some understanding of their physiological function and molecular mechanism. In contrast, the much larger set of lncRNAs appears to be highly heterogeneous, and so far no larger ncRNA classes have been identified with confidence. At least at the level of the primary sequence, lncRNAs appear to be poorly conserved [11,12], although in many cases they can be traced back over very large phylogenetic distances (see [13,14] for examples). The question to what extent pervasive transcription – either by the actions of the transcripts produced or by the process of transcription itself – is of functional relevance, however currently remains unanswered.

The number of reports on the function of individual lncRNAs is, however, rapidly growing. Many lncRNAs have been found to be involved in epigenetic processes. Several lncRNAs appear to act in *trans*, targeting chromatin-modifying enzymes and/or the proteins associated with them at their sites of action in the genome [15-17]. Recent studies suggest this as a rather common function of lncRNAs [18]. Epigenetic action in *cis* has been demonstrated at the cyclin D1 (*CCND1*) gene, where an ncRNA tethered to the promoter region recruits proteins that repress *CCND1* transcription, at least in part by inhibiting histone acetyltransferase activity [19]. Similarly, the EVF2 ncRNA has been found to recruit either the DLX2 homeobox protein to transactivate the adjacent *DLX5*/*6* gene or the transcriptional repressor *MECP2*[20,21]. lncRNAs can also serve as backbones in the structural organization of large protein complexes, like the NEAT1 RNA in paraspeckles [22]. Finally, several ncRNAs are involved in localizing or sequestering proteins in transcription factor complexes. The NRON RNA, for example, controls nuclear trafficking and dephosphorylation of the transcription factor NFAT [23,24]. The pleiotropic ncRNA GAS5 has recently been shown to sequester the glucocorticoid receptor and thus prevent its activity as a transcriptional activator [25]. Modulation of protein activity has also been observed for a coding RNA, i.e. the TP53 mRNA binds to and modulates the MDM2 protein [26]. Competitive endogenous RNAs can sequester microRNAs to regulate mRNA transcripts with target sites for the same microRNAs [27-29].

Relative to the extent of identified non-coding transcription, however, the number of lncRNAs for which a function has been demonstrated or is assumed is still minute. Reports of a high cell-type specificity for lncRNAs [4,12,30] or the differential expression of many lncRNAs throughout neuronal cell differentiation [31], however, hint at a more global relevance of non-coding transcription.

We argue that over the last decades: (i) the identification of protein-coding mRNAs found to be differentially expressed in the context of important cell-physiological processes has frequently led to the discovery of proteins with critical functions and (ii) that the differential expression of many such transcripts turned out to be associated with disease. We therefore hypothesize that lncRNAs that are differentially expressed in such processes are also likely to play functional roles. Although a number of ncRNAs have been demonstrated to be regulated by cellular signaling pathways, a systematic survey of ncRNAs that are transcriptionally controlled by such pathways is still lacking. We therefore focused here on three oncologically relevant pathways and processes to determine the extent to which these pathways – in addition to their known protein-coding target genes – also control the expression of ncRNAs. For this purpose, we chose the signal transducer and activator of transcription-3 (STAT3) pathway, the p53 pathway and cell-cycle regulation. Each of these systems is intimately involved in tumor development.

The tumor suppressor p53 is activated in response to DNA damage as well as other stress signals and in turn induces DNA repair, growth arrest and apoptosis. As a transcription factor, p53 acts by binding to specific DNA elements in the promoter and enhancer regions of target genes, thereby controlling their transcription. Several lncRNAs that are induced by the p53 pathway and involved in the regulation of p53 target genes have been identified [17]. In turn, ncRNAs that modulate the p53 function have also been reported, e.g. the lncRNAs RoR [32] and MEG3 [33].

STAT3, originally identified and characterized by us as the central signal transducer for the interleukin-6 family of cytokines [34,35], has been shown to be a strongly oncogenic pathway [36]. Constitutively active STAT3 is found in many cancers, and STAT3 has been proved to be an essential component acting downstream of many other oncogenes [37]. Although contributing a proliferative signal as well, the STAT3 pathway is primarily known for its strong anti-apoptotic effect in many tumor cells. We previously reported that the control of known apoptosis regulators by STAT3, however, does not sufficiently explain its strong survival effect on human multiple myeloma cells [38]. We demonstrated that the gene for the microRNA miR-21 hosts a phylogenetically conserved enhancer harboring two STAT3 binding sites and that the induction of this ncRNA critically contributes to the anti-apoptotic and oncogenic potential of the STAT3 pathway [39]. This raised the question as to whether STAT3 might control the transcription of other ncRNAs as well.

Cell-cycle regulation resides at the core of tumor development and progression. A tightly controlled cellular machinery defines the pace of proliferation and the highly ordered progression through the cell-cycle phases G1, S, G2 and M. This machinery employs a number of critical oncogenic and tumor-suppressing components, like cyclins and cyclin-dependent kinase inhibitors, respectively. Our knowledge of the involvement of ncRNAs in cell-cycle regulation is, however, rather limited. Remarkably, Hung *et al*. reported the extensive transcription of ncRNAs from the promoter regions of cell-cycle genes [40], suggesting that ncRNAs do in fact play a role in this process.

Here, we used tiling arrays as an unbiased transcriptomic technique to study the differential expression of lncRNAs: (i) throughout the cell cycle, (ii) controlled by the pro-apoptotic and anti-proliferative p53 pathway and (iii) controlled by the pro-proliferative and anti-apoptotic STAT3 pathway. We showed that a large set of lncRNAs of diverse properties are differentially expressed in response to these pathways and that up to 87% of the transcriptional response can be non-coding. Among the differentially expressed lncRNAs we identified a set of very long, highly cell-type specific macroRNAs. We demonstrated that these macroRNAs are likely continuous transcripts, despite their size of up to 400 kb. We investigated the evolution of the macroRNA STAT3-induced RNA 1 (STAiR1), and found that it contains highly conserved elements, which maintain their spacing during eutherian evolution and partly exhibit RNA secondary structure under stabilizing selection. Based on a comparison of evolutionary rates with adjacent protein-coding genes, we argue that STAiR1 likely acts locally. Finally, we investigated lncRNA expression using the nONCOchip custom array for astrocytoma, a tumor disease not related to the cell lines initially used, and found differential expression of macroRNAs between different grades of the disease.

## Results and discussion

### Global unbiased assessment of transcriptional activity

We first strove to identify transcriptional activity dependent on cell cycle, pro- and anti-proliferative stimuli. We decided to use cellular systems that give the clearest results for each pathway and process, instead of one common cell line.

RNA expression in response to STAT3 activation, as a pro-proliferative anti-apoptotic and oncogenic stimulus, was studied using the human multiple myeloma cell line INA-6. The growth and survival of these cells critically depends on IL-6, and we have shown previously that the IL-6 signal is transduced almost exclusively by STAT3 in these cells [38]. RNA was isolated from: (i) INA-6 cells deprived of IL-6 for 13 h, (ii) cells after 1 h of restimulation and (iii) cells permanently cultured in IL-6. STAT3 activation upon IL-6 restimulation is shown in Additional file 1: Figure S1.

Transcriptional activity under p53 expression as an anti-proliferative pro-apoptotic tumor suppressor stimulus was studied in D53wt cells. This human colorectal carcinoma cell line harbors a defunct endogenous p53 and was stably transfected with tetracycline-responsive wild-type p53. RNA was isolated from cells grown in the presence of tetracycline (control) and 6 h after tetracycline removal (p53 induced). p53 induction is shown in Additional file 1: Figure S2.

The expression of RNA throughout cell-cycle phases was studied by synchronizing human primary foreskin fibroblasts in G0 using serum starvation for 48 h. Cells were harvested before and 14 h, 20 h and 24 h after addition of serum. The cell-cycle phase distribution was examined using flow cytometry (Additional file 1: Figure S3). The time points 14 h, 20 h and 24 h correspond to a maximal enrichment relative to the other phases, G1, S and G2, respectively.

**Figure 1:**
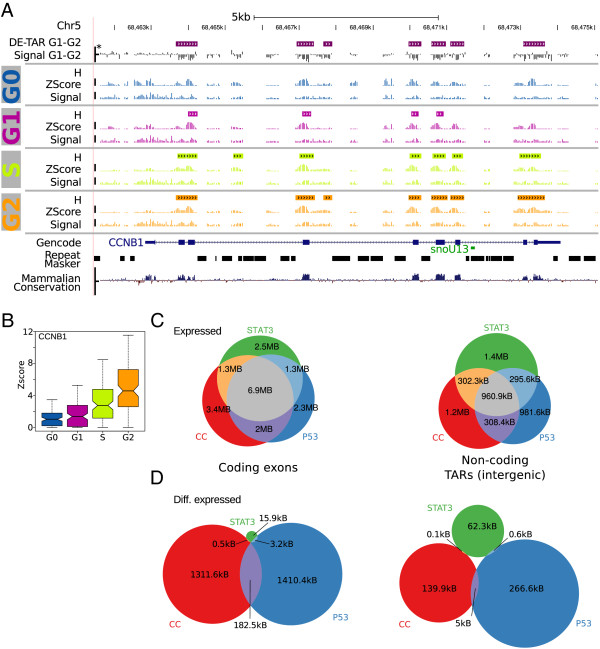
**Differentially expressed TARs (DE-TARs).(A)** The *CCNB1* locus, a positive control for cell-cycle, illustrating the tiling array data analysis workflow employed. For each condition (in this case the cell-cycle phases G0, G1, S and G2), the raw tiling array signal intensities (*Signal*) in overlapping sliding windows of 200 nt were evaluated to see if the expression was significantly higher than a background distribution, using the TileShuffle algorithm with *q*<0.05. The background distribution was generated from 10,000 GC controlled permutations of the individual array’s signals. Overlapping windows of significant expression were summarized to intervals labeled *H*. Analogously, differentially expressed intervals were generated for each pairwise comparison of interest for all intervals designated *H* in at least one condition of the dataset. Difference signals in windows of the same size were evaluated for a significantly higher differential expression than a background of 100,000 difference shuffles, with *q*<0.005 and labeled DE-TAR intervals. Repeat masked intervals are missing in the array design due to the ambiguity of probes mapping to these regions. (*) Wiggle track scale bars indicate *y*-axis scales of (6,16), (0,10), (-3.5,3.5) and (-4,4) for the signal, *z*-score, differential signal and conservation, respectively. **(B)** Expression signal from **(A)** aggregated over all exons of *CCNB1*. Boxes indicate the median, first and third quantiles. Notches are placed at $\pm 1.58\;\text{IQR}/\sqrt{n}$ and approximate a robust 95% confidence interval. **(C)** Overlap in expressed nucleotides between STAT3, p53 and cell-cycle (CC) datasets for known coding exons (Gencode v12, UCSC genes, Ensembl and RefSeq) and *bona fide* non-coding intergenic TARs. **(D)** Overlap between the three datasets in differentially expressed nucleotides. CC, cell cycle; Chr, chromosome; DE-TAR, significantly differentially expressed TAR; IQR, interquartile range; kb, kilobase; MB, million base pairs; TAR, transcriptionally active region.

Global RNA expression was analyzed using Affymetrix whole genome tiling arrays, which interrogate the non-repetitive part, i.e. approximately 40%, of the human genome. Transcriptionally active regions in the genome (TARs) were identified using TileShuffle[41]. Briefly, TileShuffle identifies segments in the tiling array data that are expressed significantly higher than an affinity controlled background distribution. Figure 1A illustrates the performance of this procedure, when applied to *cyclin B1* as a positive control for the cell cycle [42]. As expected, *cyclin B1* was marginally expressed in G0, increased during cell-cycle progression and peaked in the G2 phase (Figure 1B). Fragmentation of the expressed intervals due to signal variation and the lack of knowledge on exon-exon junctions for non-annotated transcripts results in numbers of expressed fragments that are somewhat arbitrary for tiling array data. Following [41], we therefore report the number of expressed, differentially expressed or overlapping nucleotides rather than fragment numbers throughout the manuscript. We identified 19 million base pairs (Mb) to 21 Mb, 20 Mb to 22 Mb, and 17 Mb to 21 Mb expressed for the STAT3, p53 and cell-cycle experiments, respectively (Additional file 1: Table S1).

### *Bona fide* non-protein-coding RNAs exhibit higher cell type specificity

One goal of this analysis was to identify the extent of non-coding transcription in response to pathway actuation. For novel significantly differentially expressed TARs (DE-TARs) overlapping or containing open reading frames we cannot formally rule out expression at the proteome level. We therefore defined the set of *bona fide* non-coding TARs as genomic intervals that did not exhibit any signal for protein-coding potential in a state-of-the-art bioinformatic approach. More specifically, *bona fide* non-protein-coding TARs were defined as TARs that are intergenic and have neither predicted protein-coding potential according to RNAcode (*P*<0.05) nor any obvious similarity with protein-coding sequences as detected by tblastn (*e*<0.05, RefSeq database from 7 March 2012). As expression was analyzed in three different cellular systems, we investigated the cell type specificity of TARs and observed a substantial overlap (Additional file 1: Figure S4). This overlap was mainly due to protein-coding exons. *Bona fide* non-protein-coding TARs were expressed in a more cell type-specific manner than coding exons (Figure 1C). The same holds for *bona fide* non-protein coding TARs detected in introns of known protein-coding genes (Additional file 1: Figure S5). The higher cell type specificity of non-coding expression is in line with observations for the ENCODE pilot phase [4] and subsequent studies [12,30], but in contrast to reports by Ørom and colleagues [43].

### Differentially expressed segments are highly pathway specific

TileShuffle was used again to identify differentially expressed segments. To prevent the misidentification of differential expression due to noise close to the detection limit, we restricted the analysis of differential expression to segments that were classified as significantly expressed in at least one of the compared states (cf. Figure 1A). For assessing differential expression, TileShuffle again relates the differential expression in an interval under consideration to a background distribution obtained by permuting log signal differences between the two arrays of interest. We identified 28 kB to 118 kB, 4 Mb, and 9 kB to 1 Mb nucleotides corresponding to 130 to 394, 12,290, and 53 to 5,057 differentially expressed segments for the STAT3, p53 and cell-cycle experiments, respectively (Additional file 1: Table S2).

DE-TARs were far more specific for the investigated pathway or cell type – which we cannot strictly discriminate in this setup – than expressed TARs (Additional file 1: Figure S6). While the overlap was small for coding exons, it was negligible for *bona fide* non-coding intervals (Figure 1D, Additional file 1: Figure S7). DE-TARs differentially expressed upon STAT3 activation hardly overlapped the other two experiments. In contrast, the observed substantial overlap of about 300 kB between p53 and cell-cycle DE-TARs likely reflects the role of p53 in cell-cycle control.

Whole genome tiling array experiments are demanding of RNA material. This was particularly problematic for the cell-cycle experiment. To allow estimation of false discovery rates (FDRs) in replicated experiments with less material and subsequent quantification of identified TARs in clinical material, we designed a custom array that interrogates a representative subset of the identified TARs. This custom array, called nONCOchip, additionally interrogates the set of human RefSeq mRNAs, structured ncRNAs predicted with RNAz[44] and evofold[45], and human ncRNAs from public databases (see Additional file 1: Tables S11 and S18 for details). Using the nONCOchip in biological triplicates as a reference, we estimated FDRs between 0.18 and 0.33 (Additional file 1: Figure S8).

### *Bona fide* non-coding significantly differentially expressed transcriptionally active regions are enriched for annotated long non-protein-coding RNAs but largely novel

We determined the extent to which differentially expressed segments overlapped annotated coding and non-coding transcripts, and computed the number of nucleotides overlapping between DE-TARs and Gencode v12 annotations [46] or additional sources for ncRNAs listed in Additional file 1: Table S28. To assess whether a similar overlap would have been observed by randomly distributing the DE-TARs over the genome, we computed odds ratios for the relative overlap for DE-TARs and annotation versus the relative overlap for annotation and genomic intervals that have been sampled repeatedly and randomly, while preserving the length distribution and repeat content of the original DE-TARs.

**Figure 2:**
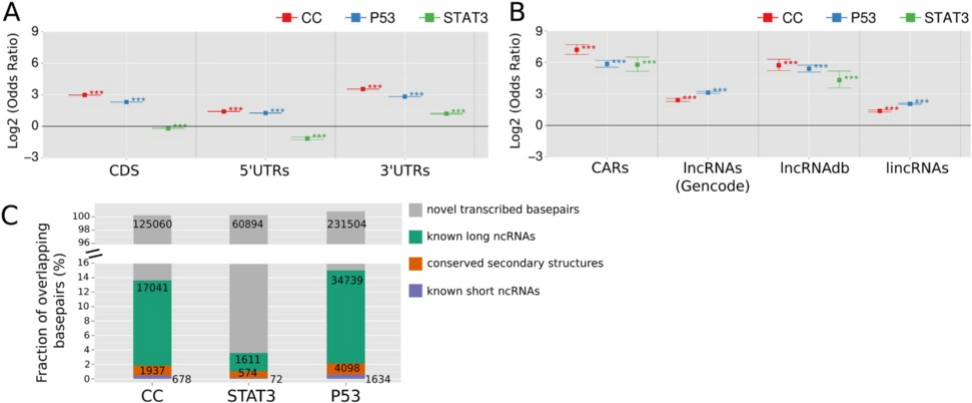
**DE-TAR overlap with genomic annotation.(A,B)** Overlaps in nucleotides between DE-TARs and different annotation categories. Log_2_ transformed odds ratios and their 95% confidence interval for the respective annotation dataset are shown (annotations are described in detail in Additional file 1: Table S28). To assess the significance of the observed overlap, 100 lists containing random intervals from the genome controlling for repeat content and DE-TAR length were sampled. Odds ratios of observed versus randomized relative overlaps were calculated and tested using Fisher’s exact test for significant enrichment or depletion. *** indicates *P*<0.001 for the observed versus random nucleotide overlaps, ** *P*<0.01 and * *P*<0.05. Results are shown for DE-TARs that overlap annotated protein-coding genes **(A)** (additional annotations are shown in Additional file 1: Figure S10) and *bona fide* non-coding DE-TARs that overlap with several classes of experimentally verified and predicted ncRNAs **(B)** (additional annotations shown in Additional file 1: Figure S11). For the detailed output of Fisher’s exact tests refer to Additional file 1: Tables S4 and S6. **(C)** Fraction of nucleotides in intergenic *bona fide* non-coding DE-TARs overlapping with known long ncRNAs (large intergenic non-coding RNAs and transcripts of unknown protein-coding potential as identified in [30], Gencode v12 long ncRNAs, lncRNAs found in the Long Non-Coding RNA Database (lncRNAdb, [49]) and ncRNAs found in chromatin [50]), short RNAs (UCSC sno/miRNA track), conserved secondary structures (Evofold[45], RNAz[44,51] and SISSIz[52]) and novel transcribed nucleotides. CAR, chromatin-associated RNA; CC, cell cycle; CDS, coding sequence; lncRNA, long ncRNA; ncRNA, non-protein-coding RNA; UTR, untranslated region.

As expected, cell-cycle and p53 DE-TARs were found to be strongly enriched for known protein-coding RNAs (Figure 2A, Additional file 1: Figure S10). Although STAT3 is known to regulate the expression of many mRNAs, STAT3 DE-TARs were not enriched for coding sequence (CDS) and 5^′^ UTRs and had only low enrichment in 3^′^ UTRs. This may hint at a particular prominence of non-coding transcription among the targets of STAT3. The salience of 3^′^ UTRs might be a consequence of an independent expression or processing of 3^′^ UTRs, which has been reported by others [47,48]. However, we found only a few cases where this was plausible (Additional file 1: Figure S9 and Table S3).

Pathway-controlled intergenic, *bona fide* non-coding DE-TARs were enriched for previously experimentally identified lncRNAs, which corroborates our experimental approach and strategy for *bona fide* non-coding filtering (Figure 2B, Additional file 1: Figure S11A). While all three pathways resulted in enrichment for chromatin-associated RNAs [50] and lncRNAdb annotations [49], only cell-cycle and p53 were enriched for lncRNAs from Gencode and lincRNAs from the expression atlas by Cabili and colleagues [30]. This outcome may suggest that the tissue distribution of DE-TARs controlled by these pathways is broader than that of STAT3 DE-TARs.

In line with the biological role of the pathways we have triggered, we observed DE-TAR overlaps with lncRNAs of known tumor relevance like MALAT1 [53,54], MEG3 [55] and GAS5 [56]. A more comprehensive list of prominent lncRNAs overlapping DE-TARs is given in Additional file 1: Table S10. With D53wt cells, we did not observe expression of the p53-controlled lincRNA identified by Huarte and colleagues for mice [17]. The human ortholog has only partial sequence complementarity with the murine locus but seems to be inducible by DNA damage in fibroblasts. However, expression of this transcript appears to be highly context dependent, as no spliced transcript could be identified at the human locus in several fibroblast RNAseq datasets from ENCODE (data not shown).

Intergenic *bona fide* non-coding DE-TARs were enriched for H3K4me3 and H3K36me3, patterns that have been used previously for identification of lincRNA loci [57] (Additional file 1: Figure S11B). Also, all three pathways seem to trigger transcription from enhancer sequences, as we observed an enrichment for the enhancer mark H3K4 mono-methylation (H3K4me1) and acetylated H3K27 (H3K27ac), which has been found to discriminate active versus poised enhancers [58].

Despite many overlaps with annotated ncRNAs, the majority of intergenic *bona fide* non-coding DE-TARs represent novel transcripts. Overlaps with annotated RNAs account for only 4% (STAT3) to 15% (p53), with the majority being overlaps with annotated lncRNAs (Figure 2C).

### STAT3-induced macroRNAs

**Figure 3:**
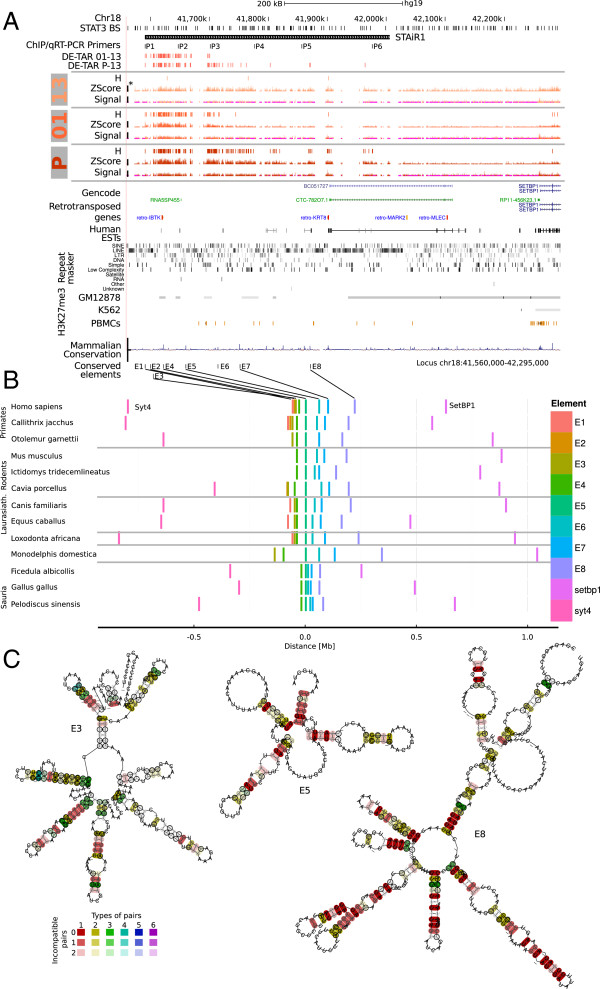
**STAiR1 – a STAT3-controlled macroRNA.(A)** STAiR1 is upregulated in response to STAT3 and was identified by manual inspection of TileShuffle tracks. After 1 h of restimulation with IL-6 (denoted 01 on the left), TileShuffle detects a 130-kB long region of significant upregulation compared to 13-h IL-6 withdrawn cells (13). In cells permanently cultured with IL-6 (P), the region extends to at least 300 kb. It overlaps H3K27me3 domains in ENCODE data identified in GM12878 lymphoblastoid cells and peripheral blood mononuclear cells (PBMCs) derived from healthy donors, which is missing in K562 leukemia cells [5], and several STAT3 binding sites (STAT3 BS). Please refer to the caption of Figure 1, for a definition of signal, H, and DE-TAR tracks and wiggle track scale bars. **(B)** STAiR1 contains highly conserved elements. STAiR1 was aligned to all vertebrate genomes provided by Ensembl using BLAST[64]. Several conserved elements throughout STAiR1 that did not overlap annotated repeat elements were selected for further analysis. The chart displays the relative location of elements E1 to E8, arbitrarily aligned by E6 for selected genomes. Hits in additional genomes, including those where no continuous scaffold was available for the interval E1 to E8, are shown in Additional file 1: Figure S14. **(C)**BLAST hits from **(B)** were initially aligned using Clustalw[65], submitted to RNAalifold[66] and trimmed to regions of conserved secondary structure. The depicted consensus RNA secondary structures were generated by applying LocARNA[67] followed by RNAalifold to the trimmed sequences. The number of different types of base pairs for a consensus pair, i.e. compensatory mutations supporting the structure, is given by the hue, the number of incompatible pairs by the saturation of the consensus base pair. ChIP, chromatin immunoprecipitation; Chr, chromosome; DE-TAR, significantly differentially expressed transcriptionally active region; EST, expressed sequence tag; kb, kilobase; Laurasiath, Laurasiatheria; MB, million base pairs; PBMC, peripheral blood mononuclear cell; PCR, polymerase chain reaction; qRT-PCR, quantitative real-time reverse transcriptase PCR; STAiR, STAT3-induced RNA; STAT3, signal transducer and activator of transcription-3.

Manual inspection of the STAT3 experiment tiling array data identified an intergenic region of at least 300 kb in length that was contiguously upregulated upon STAT3 induction. The region was termed STAT3-induced RNA 1 (STAiR1, Figure 3A). We subsequently identified several similar regions in this dataset, e.g. the intronic STAiR2 (Additional file 1: Figure S12) and STAiR18 (Additional file 1: Figure S13). At least at first glance, these large transcribed regions are reminiscent of imprinted macroRNAs such as *Airn*[59,60], and the highly expressed large ‘dark matter’ very long ncRNA (vlincRNA) transcripts identified in tumor cells [61-63].

**Figure 4:**
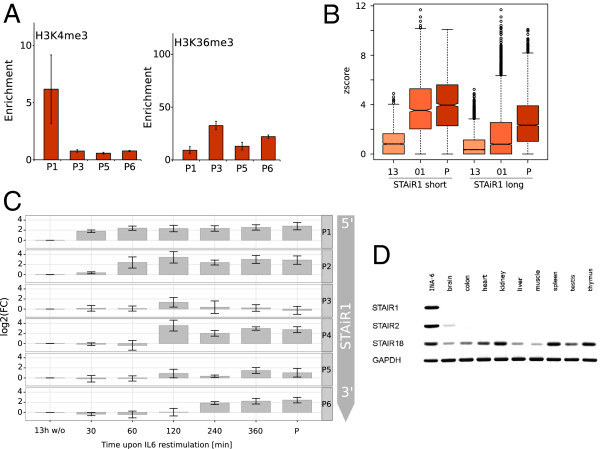
**STAiR1 – a continuous specifically expressed transcript.(A)** INA6 cells were restimulated with IL-6 as described in Figure 3A and chromatin immunoprecipitated (ChIP-ed) for tri-methylated H3K4 and H3K36, respectively. Enrichment compared to an IgG isotype control was assessed by quantitative real-time PCR using primer sets P1, P3, P5 and P6. The location of respective amplicons is shown in Figure 3A. Strong enrichment for H3K4me3 is observed only within P1, indicating an active promoter region. H3K36me3 shows strong enrichment throughout the STAiR1 transcript. **(B)** Expression *z*-score aggregated over STAiR1 expressed after 1 h (STAiR1 short, chr18:41,591,020-41,720,348) or the entire annotated STAiR1 transcript (STAiR1 long). **(C)** INA6 cells were restimulated with IL-6 as described and induction of STAiR1 was detected using qRT-PCR with primer sets P1 to P6, as shown in Figure 3A, and using GAPDH for normalization. This expression time course is consistent with the time-dependent elongation of STAiR1 observed in the tiling array data shown in Figure 3A. **(D)** Expression of macroRNAs in different tissues, as detected by reverse transcriptase PCR, using GAPDH as a normalization control. Tissue specificity varies strongly between different macroRNAs. STAiR, STAT3-induced RNA; STAT3, signal transducer and activator of transcription-3.

STAiR1 carries hallmarks of conventional polymerase II (polII) transcribed genes: using chromatin immunoprecipitation (ChIP) we identified a strong enrichment for the active promoter mark H3K4me3 compared to an immunoglobulin G (IgG) control at the transcription start site but not throughout STAiR1. Within the transcribed STAiR1 regions we observed a strong enrichment for H3K36me3, which is placed during polII transcription (Figure 4A).

Due to the ruggedness of tiling array data and the number of interspersed repeats in the human genome, a STAiR1-sized region, though strongly differentially expressed, was not reported as one continuous interval by TileShuffle, but as numerous densely placed DE-TARs. We therefore investigated whether STAiRs may represent continuously transcribed macroRNAs. STAiR1 (and similarly STAiR2 and STAiR18) was hardly expressed upon IL-6-deprivation. A strong signal covering an approximately 120-kb region was detected 1 h after restimulation, and a longer interval for cells permanently cultivated with IL-6 (Figure 4B). Both intervals seem to share a common start site (Figure 3A). PolII has been found to synthesize between 1.3 and 4.3 kb/min, corresponding to approximately 80 to 275 kb/h, although elongation can be faster under certain circumstances (see [68] and the references therein). This suggests that the joint end of both intervals represents the transcription start site of STAiR1, that the length of the observed transcript is limited by polymerase speed and so we detect the full-length transcript only under permanent IL-6 culture. We repeated this analysis for six time points, detecting STAiR1 expression using qRT-PCR (quantitative real-time reverse transcriptase PCR). Primer pairs P1 to P6 were designed so that their position roughly corresponds to the expected progress of the polymerase at different time points. We found the full-length transcript was expressed 6 h post restimulation. With the exception of primer pair P3 and the corresponding 120 min time point, qRT-PCR data were consistent with the tiling array data and thus corroborate the conclusions drawn from the tiling array data above (Figure 4C, primer positions are shown in Figure 3A). Thus, we conclude that STAiR1 is likely a continuous transcript.

STAiR1 and other STAiR-like intervals showed an apparent decay in signal intensity over the length of the transcript. We therefore investigated the tiling array signal in introns of expressed protein-coding genes as a *bona fide* set of continuously transcribed intervals. The distribution of *z*-scores along the lengths of all protein-coding genes detected by TileShuffle showed a steady decay towards their 3^′^ ends (Additional file 1: Figure S17A). Intergenic or fully intronic STAiR-like intervals displayed a similar decay (Additional file 1: Figure S17C). We therefore conclude that the observed STAiR-like intervals represent continuously transcribed macroRNAs.

### STAiR1 contains conserved structured domains and is syntenic in mammals, birds and reptiles

STAiR1 is located between two evolutionary old protein-coding genes, *SYT4* and *SETBP1*. This interval is syntenic in mammals, birds and reptiles – in rodents but not generally in Glires, synteny has been lost. Overall, STAiR1 did not exhibit a high degree of conservation (Figure 3A). However, aligning STAiR1 regions not overlapping repeats to vertebrate genomes provided by Ensembl using BLAST[64] (*e*<10^-5^) identified several conserved elements. These elements were found to maintain their order in all investigated genomes. Element E1, located at the H3K4me3-enriched region of the presumed transcription start site and element E2 were more weakly conserved (primates and Laurasiatheria). E3 was conserved in Eutheria and contained a conserved STAT3 binding site (Additional file 1: Figure S15). While for sauropsids the highly conserved elements E4 to E8 formed a more compact structure, for mammals the distances observed in human were roughly conserved. Absolute distances within these elements were more stable than to the surrounding protein-coding genes *SYT4* and *SETBP1* (Figure 3B, Additional file 1: Figure S14). Comparing the relative distance changes between man and dog to length changes of conserved introns, we found that both, including the distances to the adjacent protein-coding genes, were comparable (Additional file 1: Figure S16). We concluded that maintenance of distances within STAiR1 at a level comparable to introns of continuously transcribed genes again suggests that STAiR1 is a single transcript. Remarkably, the distances to both adjacent protein-coding genes were also constrained; however, they were rather large for distant exons. We therefore reasoned that the conserved elements are unlikely transcribed with the protein-coding genes, for which we had no evidence from the tiling array data, and that the constraint on distance rather points at some functional relevance for this distance.

Because of the constrained spacing of the conserved elements, we speculated whether these might keep some functional elements at particular distances, e.g. RNA secondary structure motifs serving as protein binding sites. We generated an initial multiple sequence alignment from the BLAST hits using Clustalw[65], computed a consensus secondary structure using RNAalifold[66] and trimmed the sequences to the regions with secondary structure. Elements E3, E5 and E8 had RNA secondary structures, which appeared to be under stabilizing selection given the number of compensatory mutations, which we observed after realigning the trimmed elements with LocARNA[67], followed by application of RNAalifold (Figure 3C).

### STAiR1 is highly specifically expressed, likely unspliced and may act locally

STAiRs showed a broad range of tissue specificity. While STAiR1 was detected in INA-6 cells only, STAiR2 was additionally expressed at a very low level in the brain but was absent from all other organs tested. In addition to its expression in INA-6 cells, STAiR18 was highly expressed in the heart, kidney, spleen and thymus while it showed low expression in the brain, colon, liver, muscle and testis (Figure 4D).

Whether or not STAiR1 may be spliced remains unclear. It overlapped a few expressed sequence tags (ESTs), some of which were spliced. However, there was no spliced EST that is confined within STAiR1 and spans a substantial region of the macroRNA. Compared to a spliced protein-coding RNA, such as CCNB1 in Figure 1A, the tiling signal of STAiR1 also did not hint at splicing. The transcript spans repetitive elements of several types, but there was no general enrichment for repeats. However, STAiR1 was significantly depleted for Alu elements, while enriched for LINE and RNA repeats (Additional file 1: Table S15).

Given the size of STAiR1 one might speculate that if it is functional, it acts rather locally or regionally. STAiR1 is located adjacent to *SETBP1*, which encodes a protein that binds to the SET nuclear oncogene and other proteins containing the SET domain. High expression of SETBP1 and SET is associated with myeloid malignancies (e.g. [69,70]), diseases in which STAT3 is a central oncogene (e.g. [71]). We hypothesized that if STAiR1 interfered *in cis* with *SETBP1*, these would exhibit similar evolutionary patterns, i.e. the substitution rates should not differ significantly. Wong and Nielsen introduced a phylogenetic model, which found faster evolution in non-coding regions compared to a protein-coding ‘reference’ gene [72]. Comparing the substitution rates detected in multiple sequence alignments of STAiR1 and *SETBP1*, we could not reject a joint model in favor of models of independent evolutionary rates (Additional file 1: Table S16). We thus concluded that STAiR1 likely acts locally.

Both STAiR1 and STAiR2 overlap domains of tri-methylated lysine 27(H3K27me3) in ENCODE data for the lymphoblastoid cell line GM12878. STAiR1 also does for peripheral blood mononuclear cells. Both cell lines were derived from healthy donors. For K562 cells from a leukemia donor, this modification is missing [5] (Figure 3A, Additional file 1: Figure S12). Given that other lncRNAs have been found to interfere with H3K27 methylation [15,16], one might speculate on the roles of STAiR1 and STAiR2 in this pathway. As these RNAs are induced by an oncogenic stimulus, and H3K27me3 marks are missing at their loci of expression in tumor cells, they might repress H3K27 methylation in *cis*.

### STAiR-like macroRNAs regulated by p53 and cell-cycle

We suspected differential expression of similar macroRNAs would also be found for the p53 and cell-cycle data. As pointed out above, STAiR-like regions cannot be reported as continuous blocks by TileShuffle. We therefore developed an algorithm to identify comprehensively long differentially expressed intervals of this type in all three experiments.

**Figure 5:**
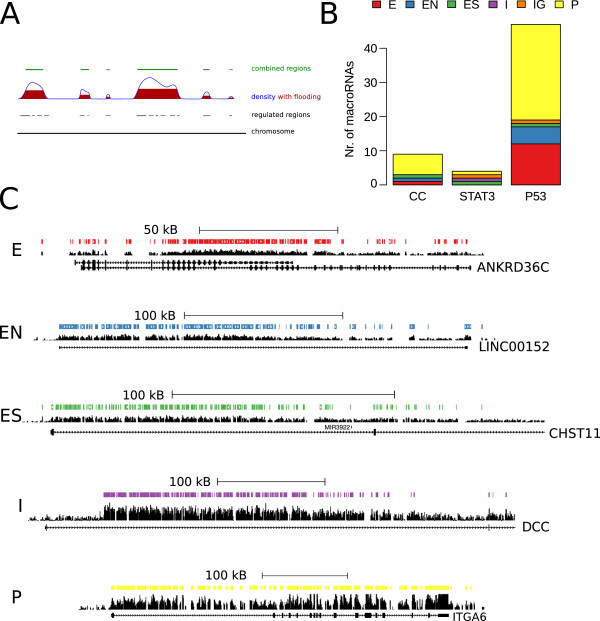
**Genomic organization of DE-macroRNAs.(A)** Schematic representation of the algorithm used to identify macroRNAs resembling the example in Figure 3A. DE and expressed intervals identified by TileShuffle are summarized as the density of positive nucleotides. Local maxima are identified and the density curve is ‘flooded’ to 50% of the local maximum to identify the boundaries of the region. Overlapping regions are merged and for each region a score based on coverage by positive nucleotides and silhouette is calculated. **(B)** Computationally identified macroRNAs with a score > 10,000 were manually inspected to discard false positives, which are typically long protein-coding genes with many exons interspersed by small introns. Identified DE-macroRNAs fall into different genomic categories: intergenic (IG), overlapping exons (E), overlapping non-coding exons (EN), located in introns (I), joint start but different end as coding RNA (ES) and presumed primary transcript (P). **(C)** DE-macroRNA examples for the E, EN, ES, I and P cases. The IG case is illustrated in Figure 3A. Only *z*-scores and selected transcript isoforms are shown. CC, cell cycle; E, overlapping exons; EN, overlapping non-coding exons; ES, joint start but different end as coding RNA; I, located in introns; IG, intergenic; kB, kilobase; Nr, number; P, presumed primary transcript; STAT3, signal transducer and activator of transcription-3.

The stairFinder algorithm uses a flooding approach for the density of TARs and DE-TARs to identify STAiR-like intervals in tiling array data (Figure 5A). While stairFinder reliably identifies STAiR-like regions in the tiling array data, it only ranks the RNAs according to a score combining coverage of the identified region and its silhouette. It cannot discriminate, however, between weakly differentially expressed STAiR-like regions and multi-exon genes with many exons separated by short introns. We therefore manually curated the stairFinder output to obtain a list of *bona fide* STAiR-like intervals.

**Figure 6:**
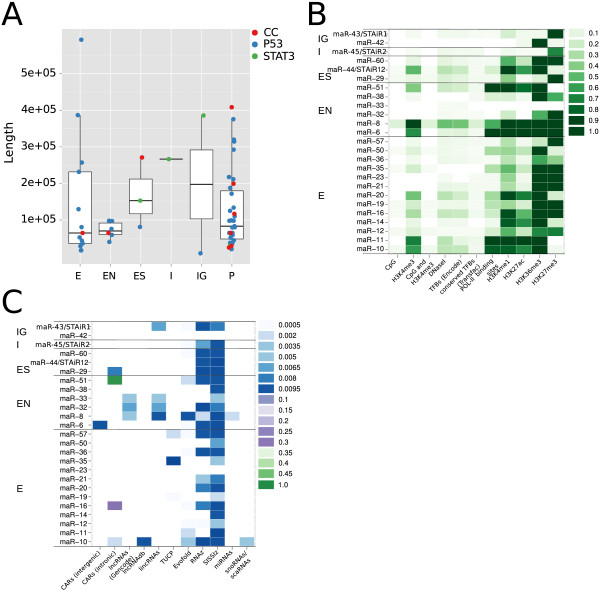
**Characterization of DE-macroRNAs.(A)** The size distribution of DE-macroRNAs indicates similar sizes for the different genomic categories of DE-macroRNAs (intergenic, overlapping exons, overlapping non-coding exons, located in introns, joint start but different end as coding RNA and presumed primary transcript) and throughout the three different transcriptome surveys (cell cycle, p53 and STAT3). **(B)** Fraction of nucleotides in DE-macroRNAs overlapping with putative promoter regions, transcription factor binding sites, polII binding sites and epigenetically modified regions. **(C)** Fraction of nucleotides in DE-macroRNAs overlapping with known ncRNA annotations. Annotations are described in detail in Additional file 1: Table S28. CC, cell cycle; E, overlapping exons; EN, overlapping non-coding exons; ES, joint start but different end as coding RNA; I, located in introns; IG, intergenic; P, presumed primary transcript; STAT3, signal transducer and activator of transcription-3.

Using stairFinder, we identified STAiR-like regions for the p53 and cell-cycle experiments as well. Overall, we found 60 such differentially expressed regions of at least 10^4^ nucleotides in length (Figure 6A, Additional file 1: Table S12). Applying stairFinder to expressed intervals, we found numerous STAiR-like regions (Additional file 2). Roughly, six types of STAiR-like intervals in DE-TARs can be derived due to their genomic organization: (i) fully intergenic, (ii) fully intronic, (iii) overlapping annotated exons, (iv) overlapping annotated exons of non-coding RNAs, (v) regions that start with annotated transcription start sites of protein-coding genes that do not, however, show intron/exon structures and terminate in an intron of the gene and (vi) intervals starting at known transcription start sites and ending at known termini of protein-coding genes, thus most likely representing accumulating primary transcripts. The latter does not necessarily exclude a function at the RNA level, at least not for primary transcripts of lncRNAs. The Air macroRNA appears to function as an unspliced long RNA although spliced transcripts have been identified [73]. The distribution of these types in the different experiments is shown in Figure 5B and examples are given in Figure 5C. The different types of macroRNAs have similar size distributions (Figure 6A).

In DE-TARs, most STAiR-like intervals were found for the p53 experiment. Many of these fall into the category of presumed primary transcripts. Since in this experiment an exogenous TP53 overexpression was used, it cannot be formally ruled out that this high number of STAiR-like intervals was in part due to unphysiological TP53 levels. STAT3 activation by IL-6 in INA-6 cells is a physiological way of activating the transcription factor. However, STAiRs expression might be a consequence of the many genomic aberrations found in INA-6 cells. In contrast, no such artifacts are expected in the primary fibroblasts used for the cell-cycle experiment, where we also identified several STAiR-like regions. We therefore conclude that we did observe a physiological process.

Ignoring suspected primary transcripts, a majority of the macroRNAs overlapped ENCODE H3K36me3 domains and polII binding sites (Figure 6B), substantiating that most of these transcripts are generic polII products. As already demonstrated for STAiR1, many of these macroRNA loci included H3K27me3 sites. Furthermore, the majority of them seemed to contain enhancers, indicated by H3K4 mono-methylation (H3K4me) and acetylated H3K27. Several macroRNA loci also contained promoter sites with H3k4me3 but only a few contained these modification in CpG islands.

Two of the macroRNAs identified here had a substantial overlap with intronic chromatin-associated RNAs [50], and four overlapped the vlincRNAs from [63]. Of these, maR-31 is presumably a primary transcript, maR-33 an annotated spliced lncRNA linc0278, maR-42 a strongly p53-induced intergenic macroRNA, and maR-57 a snoRNA (small nucleolar RNA) host gene. Also, we observed significant expression of KCNQ1OT1 in p53-induced cells, a macroRNA well known to be involved in imprinting. Hardly any overlap was found with lncRNAs annotated in Gencode or lncRNAdb or detected by Cabili and colleagues (Figure 6C). Johnson and colleagues reported a set of REST-controlled macroRNAs, which are, however, not conserved in human [74].

### Pathway-controlled long non-coding RNA expression in an independent brain tumor disease

Given the important role of cell-cycle regulation, p53 and STAT3 in oncogenesis, we hypothesized that pathway-controlled lncRNAs could be of more general relevance in tumor diseases. We therefore investigated expression of the identified DE-TARs in a tumor disease where the selected pathways are of key importance, but which was otherwise not closely related to the cells used for identification of pathway-controlled DE-TARs. We used the above-mentioned nONCOchip custom microarray to investigate RNA expression in different grades of astrocytoma, a neoplasia of glial cells in the brain. Four samples of each of WHO grade I (associated with good prognosis), grade III and grade IV (i.e. primary glioblastomas) astrocytomas were used [75]. Grades III and IV are associated with an increasing reduction in median survival time (Additional file 1: Table S17).

**Figure 7:**
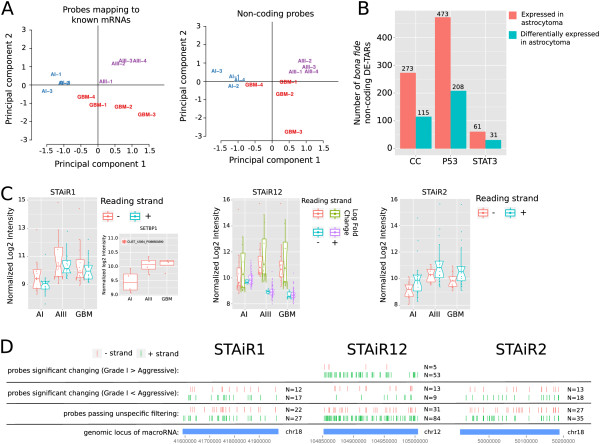
**Disease-associated ncRNAs.** The custom microarray was used with diffuse astrocytoma samples of different grades. **(A)** Principal component analysis for probes passing non-specific filtering. For at least four samples, the expression of the probe must be larger than the background, where background expression is defined by the mean intensities plus three times the standard deviation of negative control spots. The probe must have a non-specific change of expression of IQR>0.5. A separate principal components analysis was done for probes mapping to exons of known protein-coding genes (Gencode v12) and probes for which no evidence of short open reading frames was detected (see Materials and methods). The first two principal components accounted for 55% and 63% of overall variation, for probes mapping to exons of protein-coding genes and *bona fide* non-coding probes, respectively. **(B)** Number of *bona fide* non-coding DE-TARs either expressed in astrocytoma, i.e. overlapping at least one probe with an intensity larger than the background intensity for at least four samples, or differentially expressed in astrocytoma, i.e. overlapping at least one probe significantly differentially expressed between astrocytoma of grade I and aggressive states of grades III and IV (glioblastoma) (FDR<0.05). **(C)** Box plots depicting normalized log_2_ intensities of all probes significantly regulated in astrocytoma (grade I compared to aggressive states, FDR<0.05) and located in the genomic loci of three selected macroRNAs, STAiR1, STAiR12 and STAiR2. Notches depict 95% confidence interval of the median intensity. Normalized log_2_ intensities of a significantly regulated probe corresponding to *SETBP1*, a gene proximal to STAiR1, is shown next to the STAiR1 plot. **(D)** Overview of probe positions of probes located in the genomic loci of STAiR1, STAiR12 or STAiR2. CC, cell cycle; DE-TAR, significantly differentially expressed transcriptionally active region; GBM, glioblastoma; IQR, interquartile range; STAiR, STAT3-induced RNA; STAT3, signal transducer and activator of transcription-3.

Using principal components analysis on the expression data of all mRNAs that passed unspecific filtering, we observed a clear separation into the three grades, when plotting the two first principal components. A similar quality of separation into grades was obtained using *bona fide* non-coding RNAs. This indicates that the investigated non-coding RNAs and mRNAs convey similar degrees of disease information (Figure 7A). This is in line with several other observations that lncRNA expression patterns have diagnostic potential in tumor diseases (e.g. [76,77] and references therein).

Overall, we found 13,308 probes differentially expressed between astrocytomas of grade I compared to the aggressive states grades III and IV, 5,550 of which are *bona fide* non-coding and map to 126 known non-coding long ncRNAs (Gencode v12) (Additional file 1: Table S19). However, we observed comparably few differentially expressed probes that corresponded to pathway-controlled DE-TARs. This appeared to be mainly a consequence of the specific expression of pathway-controlled DE-TARs. Of those expressed at all in astrocytoma (807), more than 40% were differentially expressed between grades (354, Figure 7B).

Differentially expressed probes were mainly enriched for the known lncRNAs represented on the custom array: intergenic chromatin-associated RNAs, Gencode-annotated lncRNAs, annotations in lncRNAdb, Cabili’s catalogue of lincRNAs and snoRNAs (Additional file 1: Figure S18A).

### STAT3-controlled macroRNAs are differentially expressed between grades of astrocytoma

Despite the low number of differentially expressed probes mapping to DE-TARs, we observed remarkable enrichment in three STAT3-controlled macroRNAs. STAiR1 and STAiR2 were both upregulated in aggressive forms of astrocytoma (Figure 7C,D). STAiR12 was primarily downregulated; however, there appeared to exist another transcriptional unit, which was upregulated but represented by fewer probes. For STAiR1 and STAiR2, induction was observed on both strands. However, more significantly differentially expressed probes were located on the + strand, which corresponds to the direction of transcription that was inferred from the tiling array signals. We also observed upregulation of the *SETBP1* gene in aggressive grades and thus correlated expression with the adjacent STAiR1. This might again hint at some *cis* interaction between those two transcripts, which we already suspected based on comparison of evolutionary rates. Apart from STAiR1 and *SETBP1*, we observed a number of pairs of adjacent lncRNA and disease-relevant mRNA with a correlated or anti-correlated expression pattern (Additional file 1: Figure S19 and Table S23, and Additional file 2).

## Conclusions

### Pathway-controlled non-coding transcription

The extent of functionality among the mass of non-coding transcripts that have been discovered over the last few years is one of the pivotal questions in current genomics. Here, we show that just three crucial regulatory processes of the cell control the expression of as many as approximately 17,000 genomic segments within the human genome. Assuming an average of three to five exons per transcript, this may roughly correspond to 3,000 to 5,000 distinct transcripts. Among the well-known protein-coding targets of cell-cycle, p53 and STAT3, which we also picked up in our analysis, are regulators of apoptosis and proliferation as well as components of checkpoint mechanisms. It is plausible, thus, that ncRNAs controlled by the same mechanisms likely participate in these or similarly relevant control processes.

About 30% to 40% of differentially expressed segments (counting by nucleotide) in our cell-cycle experiment did not have any signal for protein-coding capacity using state-of-the-art bioinformatic procedures, and hence have to be considered as *bona fide* non-coding RNAs. This number rises to an impressive 87% for the STAT3 experiment, the majority of which is intergenic. In contrast, the regulated ncRNAs were predominantly intronic in the cell cycle and p53 datasets. This salience of STAT3 is not unexpected. The anti-apoptotic effect of STAT3 cannot be sufficiently explained by its known protein-coding targets [38] and has been attributed in part to the upregulation of miR-21 [39]. Kapranov and colleagues observed that in terms of the total mass of RNA, more than 50% of non-ribosomal transcripts are ‘dark matter RNA’, and hence non-coding [61]. We can now conclude that differential transcription triggered by signaling pathways gives rise to a similar abundance of dark matter content. We observed overlaps of *bona fide* ncRNAs, significantly enriched compared to a random control, for lncRNA annotations from Gencode[46], lncRNAdb[49], Mondal [50] and Cabili and colleagues [30]. However, despite these overlaps and the rapidly increasing number of annotated lncRNAs, more than 85% of the identified *bona fide* non-coding differentially expressed segments were novel. This extent of novelty may reflect a high degree of tissue and context dependence of ncRNA expression even within the same pathway, which might explain why we did not observe expression of the few known cases of p53-controlled lncRNAs.

### MacroRNAs controlled by cell-cycle, p53 and STAT3

We identified a series of pathway-controlled macroRNAs, very large coherently transcribed regions that likely constitute continuous transcripts. Considering their overlaps with ENCODE ChIP data, macroRNAs appear to be generic polymerase II transcripts. Most are highly pathway specific and some, e.g. STAiR2 and even more so STAiR1, exhibit a very precise tissue distribution. The macroRNAs resemble the concept of vlincRNAs, very long ncRNAs with a suspected role in neoplastic transformation [61,63]. Of our 60 differentially expressed macroRNAs, four overlap vlincRNAs completely or partially.

We found these macroRNAs in diverse genomic contexts: intergenic, intronic as well as overlapping either coding or non-coding exons. Several experiment-specific macroRNAs share boundaries with annotated protein-coding genes in particular as seen for the p53 data. As we are analyzing total RNA including non-polyadenylated transcripts, the latter are likely primary transcripts. Alternatively, we might not be able to discriminate mature transcripts and removed introns, due to the unusual stability of the latter. However, *bona fide* spliced transcripts in our datasets show tiling array signals that are very different from those of macroRNAs. Also, it appears unlikely that all macroRNA introns are equally stabilized or sequestered from the degradation machinery. A global analysis of splicing in the ENCODE project has recently shown that splicing occurs predominantly co-transcriptionally but is inefficient for lncRNAs [78]. We might therefore speculate that we should observe, in addition to macroRNAs overlapping annotated lncRNAs, a class of mRNA-derived macroRNAs that have functions at the RNA level as unspliced primary transcripts.

Pathway-controlled macroRNAs resemble in appearance transcriptional units involved in imprinting, such as mouse Air or human Airn and KCNQ1OT1 (e.g. [59,79]). Some of the examples are understood rather well, e.g. Airn, and appear to function mainly by the continuing act of ongoing transcription [79]. One might speculate that the macroRNAs we have identified play similar regulatory roles and act in a mechanistically similar manner. Although cell-type specific imprinting and thus differential expression of imprinting macroRNAs has been observed [79], there is no indication that imprinting is differential over the cell cycle or regulated by an early response pathway such as STAT3. In line with this reasoning, the only RNA known to be involved in imprinting and detected in our data, KCNQ1OT1, is not differentially expressed.

### STAT3-induced RNA 1 – a conserved, locally acting scaffold?

STAiR1 is most likely a continuously transcribed macroRNA, as we inferred from: (i) the time-dependent elongation in the tiling array and qRT-PCR data at the known speed of polymerase II, (ii) the characteristics of tiling array signals and (iii) conserved subsequences that maintain a particular spacing throughout eutherian evolution, comparable to intron length conservation of continuously transcribed mRNAs. Also, there was no hint of splicing of the entire macroRNA either from the tiling array signals or the available EST data.

The macroRNA exhibited a remarkably specific tissue distribution in non-malignant tissue. However, in astrocytoma we observed expression and upregulation in more aggressive cases. One may speculate that STAiR1 is developmentally regulated and completely shut off in adult tissues but reactivated by aggressive forms of tumors. Expression solely due to genomic aberrations in a tumor might be a different cause for this expression pattern, but it is not a consistent reason for the identified conservation pattern of STAiR1.

For the tumor tissue, we also detected correlated expression of STAiR1 and the adjacent oncogenic *SETBP1*, which might just be a coincidental consequence of the chromatin state of the entire locus, or point at some *cis* interaction between both transcripts or loci. The latter would fit with our observation that the evolutionary rates of STAiR1 and *SETBP1* are consistent with a local action of the non-coding RNA.

Conserved elements, which in part had an RNA secondary structure under stabilizing selection, support functioning at the level of the transcript as opposed to the mere act of transcription, like *Airn*. STAiR1 and STAiR2 could have a role in the local repression of H3K27 methylation since there is an apparent anti-correlation of their expression in a tumorigenic pathway and an observed lack of H3K27me3 at their loci in tumor cells of lymphoid origin, whereas there is a strong H3K27me3 signal in non-tumorigenic cells. In summary, we speculate that a possible role for STAiR1 might be local action as a scaffold for a larger ribonucleoprotein complex that promotes but is not sufficient for induction of SETBP1 and might interfere with H3K27 methylation.

## Materials and methods

### Cell culture and RNA isolation

#### STAT3

The human myeloma cell line INA-6 was maintained in RPMI 1640 medium, supplemented with 50 *μ*M 2-mercaptoethanol, 10% FCS and 100 U/ml of penicillin and streptomycin (all from Invitrogen GmbH, Karlsruhe, Germany). RNA was isolated from cells either withdrawn from IL-6 for 13 h with or without restimulation with IL-6 for 1 h or permanently maintained in the presence of 1 ng/ml IL-6 (permanent IL-6). Recombinant human IL-6 was a gift from S Rose-John (Kiel, Germany).

#### P53

D53wt cells were grown in 10% FCS in McCoys 5A modified medium containing 400 *μ*g/ml geneticin (Gibco^®^, Thermo Fisher Scientific, Waltham, MA, USA) and 250 *μ*g/ml hygromycin (Roche, Mannheim, Germany). D53wt are a derivative of the colorectal carcinoma cell line DLD-1, kindly provided by Bert Vogelstein [80]. These cells harbor an inactive 241F p53 mutant and are stably transfected with a tetracycline-responsive p53 expression system (tet-off). Induction of p53wt was performed by replacing the medium with tetracycline-free cell culture media [81]. Previous studies have shown that p53 is efficiently upregulated after 6 h [82,83]. As we were interested in identifying the direct effects of p53 transcriptional regulation, we chose to induce for 6 h for the tiling array experiment [83]. Induction of p53 mRNA and upregulation of the known p53 target gene p21^*CIP*1/*WAF*1^ was controlled by qRT-PCR (Additional file 1: Figure S2, [83]).

#### Cell cycle

Human foreskin fibroblasts obtained from ATCC (American Type Culture Collection, LGC Standards, Teddington, Middlesex, UK) were cultured in DMEM (Gibco^®^, Thermo Fisher Scientific, Waltham, MA, USA) supplemented with 10% FCS (Lonza, Basel, Switzerland). Then 10^6^ cells were subcultured in T300 flasks. After 24 h, the fibroblasts were synchronized in G0 by serum deprivation for 48 h [84]. Restimulation was carried out by adding a medium containing 20% FCS. Cells were harvested at different time points to obtain cell populations mainly at G1, S or G2/M phases of the cell cycle. Synchronized cells were analyzed by flow cytometry as described previously [82-84]. Total RNA was extracted using TRIzol^®^ (Invitrogen GmbH, Karlsruhe, Germany). The RNA integrity for each sample was controlled with the total RNA Nano Assay and the Agilent 2100 Bioanalyzer (Agilent Technologies, Santa Clara, CA, USA) [84]. All samples included in the experiments had RIN >8.

### Whole genome tiling arrays

The Affymetrix Human Whole Genome Tiling Array 1.0 Set consisting of 14 arrays was used according to the manufacturer’s instructions, except that separate labeling reactions were used for each array starting from 10 *μ*g total RNA.

### Tiling array data analysis

We used the TileShuffle algorithm described in [41] to determine expressed and differentially expressed genomic intervals in an unbiased way. Briefly, TileShuffle differentiates expression signals from background noise taking into account common tiling array biases. Windowing was used to reduce cross-hybridization effects. The significance of windows was assessed using empirical *q*-values that were estimated by repeatedly permuting probes on the array. Probes were binned with respect to the GC content of their sequences, and probes belonging to different bins may not be interchanged during permutation. The analysis of differential expression was implemented in a similar manner. Here, log-fold-changes between tiling arrays in both cellular states were used as measures of differential expression. Since sequence-specific effects were canceled out, affinity binning was obsolete in this context. We avoided considering signal intensity variation at the detection limit as differential expression by requiring that differentially expressed intervals must also be significantly expressed relative to the background distribution in at least one of the investigated conditions. Such intervals are called *DE-TARs*. This is analogous to the common non-specific filtering in conventional microarray data analysis.

Affymetrix Human Whole Genome Tiling Array 1.0 Set raw signal intensities were mapped to human genome version NCBI36 using Affymetrix BPMAP files [85]. Expressed segments were detected with the TileShuffle parameter settings: window size =200, the window score was defined as the arithmetic mean trimmed by the maximal and minimal values over signal intensities of all probes in a window, number of permutations =10,000 and number of GC classes =4. All windows with an adjusted *P*<0.05 according to Benjamini and Hochberg [86] were defined to be significantly expressed. DE-TARs are differentially expressed TileShuffle intervals with adjusted *P*<0.005 (window size =200, the window score was defined as the log-fold-change discarding all probes with converse behavior as observed for the relevant significantly expressed windows, number of permutations =100,000 and number of GC classes =1). Finally, the genome coordinates of all significantly expressed and all significantly differentially expressed segments were lifted over to GRCh37 (hg19) using [87].

### Defining a set of *bona fide* non-coding segments

Apart from overlaps with protein-coding annotation, we mainly relied on RNAcode[88] for predicting likely protein-coding segments within the TARs and DE-TARs. RNAcode considers synonymous amino acid substitutions, reading frame conservation and the occurrence of premature stop codons. It was applied to genome-wide Multiz alignments [89] for 46 vertebrate genomes downloaded from [90]. All segments with an RNAcode*P*<0.05 were considered *de novo* protein-coding regions. We refrained from adjusting *P* values for multiple testing, as we were not interested in a set of highly reliable protein-coding segments (i.e. reducing the number of false positives), but in reducing the number of regions falsely interpreted as non-coding (i.e. reducing the number false negatives). An RNAcode*P*<0.05 resulted in 84.8*%* sensitivity (according to known protein-coding exons annotated in Gencode v12) and 97.2*%* specificity (according to 10,000 sampled intergenic intervals preserving the length distribution and repeat content of protein-coding exons). RNAcode requires an input alignment of at least three evolutionarily related sequences leaving stretches of genomic DNA without an RNAcode score, because DNA sequence is not sufficiently conserved.

*Bona fide* non-coding intervals in intergenic and intronic regions were constructed from the significantly expressed and differentially expressed segments by: (i) removing all nucleotides overlapping exons of known protein-coding transcript isoforms (Gencode v12 [46], UCSC genes [91], RefSeq[92] or Ensembl[93] gene annotation) or known pseudogenes (Gencode v12), (ii) removing all nucleotides overlapping predicted protein-coding segments (RNAcode[88]), (iii) removing all segments not classified by RNAcode having a sequence similarity to known human amino acid sequences (RefSeq database from 7 March 2012, tblastn with -word-size 3[94] and *e*<0.05) and (iv) as the smallest species of ncRNAs so far described in humans – tinyRNAs and splice-site RNAs – are between 17 and 18 bp in length, the remaining intervals smaller than 17 bp were discarded.

### Detection of macroRNAs

The statistical tiling array data analysis outlined above reports segments deemed as highly or differentially expressed. Individual segments are typically short but often appear strongly enriched in large genomic intervals. Gaps within such accumulations of segments may be caused by variations in signal intensity, a drop of signal within intronic regions or by repeat regions that are not covered by the tiling array. Regions in which significant segments are highly enriched may thus reflect large biologically relevant entities. Merging segments with a maximum distance only reproduces the same picture at lower resolution but is inadequate for identifying local accumulations. Instead, stairFinder is based on estimating segment density using biweight kernels (see e.g. [95]) and a given bandwidth where segments are represented by their center position and weighted by their length. The bandwidth of the kernel is a smoothing parameter that significantly influences the resulting estimate, because a larger bandwidth tends to aggregate more segments into one single density peak. A bandwidth of 100,000 gave the best results, relative to known annotation. Each estimated peak including its flanking density minima was then processed to identify the accumulation boundaries using a flooding procedure to exclude single short outlying segments. More precisely, the boundaries were defined as the leftmost and rightmost positions between the two flanking minima where the density estimate remains above the local flooding level, which is set to the current peak multiplied by a given level parameter (0≤level≤1). We used a local flooding level of 50%. Setting the flooding level to 0 thus identifies the flanking minima as the boundary of the accumulation region. In the final step, accumulation regions that overlap with each other were combined. The stairFinder software reports these combined regions together with information on their segment coverage and silhouette as a clustering measure.

### Annotation categories

A detailed listing of annotation sets used is given in Additional file 1: Section 7.2 and Table S28.

### Statistical analysis of annotation overlaps

The overlap with annotation sets was calculated using R version 2.14.2 [96] and the Bioconductor library genomeIntervals[97]. We further used the R library Snow to enable parallel processing [98]. For each of the three experimental settings (STAT3, p53 and cell cycle), the overlap with a particular annotation set was computed in terms of: (i) the absolute number of nucleotides in the DE-TAR overlapping with a particular annotation and (ii) the odds ratio of the observed relative overlap versus a mean relative overlap of *N*=100 randomized background lists. Each background list consists of randomly generated genomic intervals of the same length distribution as observed for the corresponding DE-TARs excluding assembly gaps and repeat regions as annotated by RepeatMasker version Open-3.0[99]. Each list contains as many intervals as in DE-TAR. The sampling space for *bona fide* non-coding intergenic DE-TARs and *bona fide* non-coding intronic DE-TARs was reduced to intergenic regions (the complement of protein-coding gene annotation derived from Gencode release v12, UCSC genes, RefSeq and Ensembl genes) or intronic regions (all nucleotides not overlapping with any exon annotated as a protein-coding exon in Gencode release v12, UCSC genes, RefSeq or Ensembl genes). Observed odds, randomized odds and odds ratios are defined as follows:

(1) $$\begin{array}{ll} {\text{odds}}_{\text{DE-TAR}} & =\frac{{\text{ov}}_{\text{DE-TAR}}}{n_{\text{DE-TAR}}-{\text{ov}}_{\text{DE-TAR}}}\; \end{array}$$

(2) $$\begin{array}{ll} {\text{odds}}_{\text{BG}} & =\frac{\frac{\sum_{i}{\text{ov}}_{{\text{BG}}_{i}}}{N}}{\frac{\sum_{i}n_{{\text{BG}}_{i}}-{\text{ov}}_{{\text{BG}}_{i}}}{N}}\; \end{array}$$

(3) $$\begin{array}{ll} \text{odds ratio} & =\frac{{\text{odds}}_{\text{DE-TAR}}}{{\text{odds}}_{\text{BG}}}\; \end{array}$$

The observed number of overlapping nucleotides is given as *ov*_DE-TAR_, while ${\text{ov}}_{{\text{BG}}_{i}}$ corresponds to the number of overlapping nucleotides in the *i*th background list. The number of unique nucleotides contained in DE-TAR is *n*_DE-TAR_ and the number of unique nucleotides in the *i*th background list is $n_{{\text{BG}}_{i}}$. The significance of odds ratios was assessed using Fisher’s exact test as implemented in R. We also report the 95% confidence interval of the odds ratio, which is larger than 1 for an enriched number of overlapping nucleotides and less than 1 for depletion.

The same procedure was followed for assessing the significance of overlaps observed for significantly differentially expressed probes in astrocytoma custom microarray data. Here, the background consists of one list containing all probes on the custom microarray. A probe was interpreted as an overlapping probe if it maps by at least 90% to an interval of the relevant annotation set, and the overlap was calculated in terms of overlapping probes instead of overlapping nucleotides.

### nONCOchip design

We used Agilent 244k microarrays for realizing the custom microarray. Probes of length 60 bp where all genomic regions identified by TAS[100] are significantly differentially expressed in at least one contrast of the three different tiling array experiments were designed following Agilent’s standard design protocol for expression exon microarrays, as available from eArray[101]. Furthermore, probes for known or predicted ncRNAs derived from public databases (Additional file 1: Table S18) as well as probes for all human RefSeq mRNAs (the Agilent 014850 probeset of all mRNAs from the Agilent Whole Human Genome Microarray) were added to the microarray. The eArray was designed according to the base composition methodology where probes are equally distributed across the target sequence and the uniqueness of probes is checked against all human RefSeq RNAs. Target sequences were grouped into three length categories defining the required number of probes. Target sequences of length 60≤*l*<300 were represented by exactly one probe, while target sequences of length 300≤*l*<1,000 were represented by five probes. Target sequences longer than 1,000 bp were split into intervals of 1,000 bp and the number of probes selected according to the length of the subsequence. Probes for the plus and minus strands were designed for target sequences of unknown reading strand, i.e. sequences originating from all tiling array experiments or ncRNA predictions.

### nONCOchip processing

First, 1 *μ*g of total RNA was labeled using the Quick Amp Labeling Kit (Agilent Technologies, Santa Clara, CA, USA), according to the manufacturer’s instructions with the adaptation of using an *N*_6_-*T*7 primer instead of a polyT-T7 primer. Hybridization and scanning were performed following the manufacturer’s instructions (Agilent Technologies, Santa Clara, CA, USA). Arrays were scanned using a GenePix^®^ 4200 Scanner and the GenePix Pro^®^ 6.1 Software (Molecular Devices, Sunnyvale, CA, USA). Laser power was set to 40%, resolution to 5 *μ*m, focus to 1 *μ*m and PMT (photomultiplier tube) was set between 300 and 400 to maximize the dynamic range for each experiment. Three different biological replicates were used to estimate the FDR of the tiling array experiments.

### nONCOchip data analysis

Differentially expressed probes were identified using R version 2.14.2 [96] and the Bioconductor library limma[102]. The quality of arrays was measured by checking the distribution of ‘bright corner’ and ‘dark corner’ probes and the relative spike-in concentration compared to the normalized signal. Further, we checked whether unsupervised clustering of arrays recovered the grouping of replicates in the experiment. This resulted in good quality for all arrays. To retrieve a set of probes that map to unique genomic positions in hg19, we used BLAT[103] with parameter -minIdentity=95, allowing us to detect probes spanning splice sites. All probes that mapped to more than one distinct genomic region were discarded. Quantile normalization was used for normalizing between arrays [104]. For unspecific filtering we retained only probes that: (i) were expressed higher than the background in a predefined number of arrays and (ii) showed an interquartile range across all arrays of > 0.5. Background expression was defined as the mean intensity plus three times the standard deviation of negative control spots (Agilent’s 3xSLv spots). Finally, a linear model was fitted using the R package limma and reliable variance estimates were obtained by empirical Bayes moderated *t*-statistics. The FDR was controlled by a Benjamini–Hochberg adjustment [86].

*Bona fide* non-coding probes were identified similarly to the approach described above for DE-TARs: (i) probes were discarded if they overlapped any strand with at least one nucleotide with protein-coding exons from databases (see section on DE-TARs for details), (ii) probes were discarded if they overlapped a significant RNAcode segment (*P*<0.05) and (iii) probes overlapping segments not classified by RNAcode due to low sequence conservation were discarded if they overlapped genomic regions with a sequence similar to known human proteins (see section on DE-TARs for details). Applying these filters resulted in 53,219 *bona fide* non-coding probes in intergenic regions and 70,863 in introns of protein-coding genes. Probes antisense to protein-coding exons but not containing a significant RNAcode hit on the probe’s sense strand defined a separate set of 14,139 antisense probes.

### Identification of proximal ncRNA-mRNA pairs

For each *bona fide* non-coding probe significantly differentially expressed (FDR<0.05) between astrocytoma of grade I compared to aggressive states III and IV, the protein-coding gene (Gencode release v12) in closest genomic proximity independent of the reading strand was identified. The ncRNA-mRNA pair was retained only if the protein-coding gene was differentially expressed at the same FDR cut-off and if the fold-changes of probes mapping to exons of the same protein-coding gene had the same sign. Preconditions were used to exclude potentially non-annotated long-distance exons of the protein-coding gene: (i) if the *bona fide* non-coding DE-probe and the protein-coding gene were located on the same reading strand, only those pairs that had significant expression changes in opposite directions were used and (ii) if the *bona fide* non-coding DE-probe and the protein-coding gene were located on different reading strands, all pairs with significant expression changes were excluded.

### Gene ontology term enrichment analysis

Gene ontology (GO) term enrichment analysis for the ontology *biological process* was performed using the R library GOstats[105]. Mapping of genes to GO terms was based on the NCBI gene information table [106]. GO terms with evidence code IEA were removed in order to discard all automatically assigned annotations. GO terms with evidence codes IBA, IKR or IRD had to be removed because the version of the GOstats library used did not accept these as valid codes. The significance of enrichment was assessed by a one-sided hypergeometric test where the universe contained all genes of the custom microarray that passed non-specific filtering.

### Estimation of false discovery rate using the nONCOchip

Following [41], we ran the custom microarray in triplicate for each of the cell-cycle, p53 and IL-6 experiments with the same samples hybridized on the Affymetrix tiling arrays. The custom microarray was used as a reference to estimate the sensitivity (TP/*P*), the specificity:

$$1-\frac{\text{FP}}{N}$$

and the false discovery rate:

$$\text{FDR}=\frac{\text{FP}}{\text{FP}+\text{TP}}$$

for the DE-TARs detected on the tiling arrays. The number of true positives (TP) was the number of nucleotides that are significantly differentially expressed in the tiling array analysis (TileShuffle false discovery rate *q* ranging from 10^-4^ to 1) and overlap with nucleotides of a probe that was found significantly differentially expressed in the corresponding custom microarray experiment (FDR<0.05). The number of false positives (FP) was defined as the number of DE-TAR nucleotides that overlap with a probe that is not significantly differentially expressed in the custom microarray experiment. The number of positive nucleotides (*P*) was defined as the sum over all nucleotides of probes that are significantly differentially expressed in the custom microarray experiment. Analogously, the number of negative nucleotides (*N*) was the sum of all nucleotides of probes that are not differentially expressed.

### Detection of macroRNAs using qRT-PCR

To detect the induction of STAiR1 using qRT-PCR, total RNA was prepared from INA-6 cells (permanently cultivated with IL-6 or withdrawn from IL-6 for 13 h and restimulated with IL-6 for 30, 60, 120, 240 or 360 min) using TRIzol^®^ Reagent (Life Technologies, Carlsbad, USA). RNA was quantified using the NanoPhotometer (Implen GmbH, Munich, Germany). Then 1 *μ*g of total RNA was used for reverse transcription with the RevertAid First Strand cDNA Synthesis Kit (Thermo Fisher Scientific, Waltham, MA, USA). cDNA was amplified and detected by quantitative real-time PCR using the LightCycler™ TaqMan™ Master Kit (Roche Diagnostics, Mannheim, Germany) with a set of specific primer pairs and a hydrolysis probe from the Universal Probe Library (Roche Diagnostics, Mannheim, Germany). Signals were normalized to values obtained for glyceraldehyde-3-phosphate dehydrogenase. Sequences of the primers we used are provided in Additional file 1: Table S27.

### Tissue distribution of STAiRs

Total RNA (FirstChoice™ Human Total RNA Survey Panel, Ambion, Thermo Fisher Scientific Waltham, MA, USA) from nine different normal human tissues was reverse transcribed using specific primers. cDNA was amplified by PCR using specific primers followed by gel electrophoresis. PCR primers are listed in Additional file 1: Table S27.

### Chromatin immunoprecipitation

ChIP assays were performed according to the protocol provided with the ChIP assay kit from Upstate (Millipore, Schwalbach, Germany). INA-6 cells were either withdrawn from IL-6 for 12 h or withdrawn from IL-6 for 12 h and restimulated for 1 h. Recombinant human IL-6 was a generous gift from S Rose-John (Kiel, Germany). Cells were cross-linked by addition of formaldehyde to the medium at a final concentration of 1% and incubated for 10 min. Sonication was carried out 30 times, 30 sec on/off, level ‘high’ using the Bioruptor (Diagenode, Liège, Belgium). Aliquots containing chromatin from 3×10^6^ cells were used for immunoprecipitation with antibodies against H3K4me3 (ab1012), H3K36me3 (ab9050, both Abcam, Cambridge, UK) and rabbit IgG (# 12-370, Millipore, Schwalbach, Germany), as an isotype control. Co-immunoprecipitated DNA was amplified and detected by qRT-PCR using the LightCycler™ TaqMan™ Master Kit (Roche Diagnostics, Mannheim, Germany) and a set of specific primer pairs and a hydrolysis probe from the Universal Probe Library (Roche Diagnostics, Mannheim, Germany). Primer sequences are given in Additional file 1: Table S27.

### Non-protein-coding RNA expression profiling of brain samples

#### Patients and samples

A total of 12 patients with astrocytoma (WHO grades I and III) and primary glioblastoma (WHO grade IV) were analyzed. All patients underwent complete tumor resection and recovered without neurological deficits. All tumors were diagnosed and classified according to WHO criteria [75]. Clinical and pathological data are summarized in Additional file 1: Table S17. The study was approved by the local ethics review board at the Medical Faculty of the University of Leipzig (086-2008) and was performed in accordance with the Helsinki declaration. All patients provided written informed consent.

#### RNA extraction

After surgical resection, tumor samples were immediately frozen in DMSO (Dimethyl sulfoxide) and stored at -80°C. For RNA extraction, samples were transferred to a mortar resting in liquid nitrogen. Still in the cooled mortar, small pieces (5 to 30 mg) of tumor tissue were separated from ice and crushed mechanically. The material was then transferred directly into 1 ml TRIzol^®^ (Invitrogen GmbH, Karlsruhe, Germany), and immediately vortexed vigorously for at least 1 min. After further incubation at room temperature for 5 min, the solution was pulled up and down through a 21 gauge needle, if necessary, to dissolve all remaining visible tissue. To remove any remaining particles, the samples were centrifuged at 13,400 rcf for 10 min at 4°C. All subsequent procedures were performed on a clean bench. RNA was extracted with chloroform according to standard TRIzol^®^ protocols. GlycoBlue (Ambion Inc., Applied Biosystems, Darmstadt, Germany) was added to support precipitation. RNA pellets were washed twice with 75% ethanol and RNA was resuspended in RNase-free water. RNA samples were subjected twice to DNA digestion for 30 min at 37°C with TURBO DNA-free (Ambion Inc.) as suggested by the supplier. DNase was inactivated using the supplied Inactivation Reagent. RNA concentration and quality were assessed using a NanoDrop ND-1000 UV/VIS spectrophotometer (Thermo Scientific, Wilmington, Delaware, USA) and the RNA 6000 Nano Kit on an Agilent 2100 Bioanalyzer (Agilent Technologies, Waldbronn, Germany). Sample concentrations were adjusted to between 0.31 and 1 *μ*g/ *μ*l with RNase-free water. If necessary, ammonium acetate precipitation and resuspension in RNase-free water preceded adjustment of concentrations. RNA samples were stored at -80°C until use.

### Data availability

All expression and differential expression studies are accessible from the Gene Expression Omnibus (GEO) database. In detail, transcriptome-wide surveys for highly expressed segments during mitotic cell cycle are available through [GEO:GSE44627] (G0), [GEO:GSE44628] (G1), [GEO:GSE44629] (S) and [GEO:GSE44630] (G2M). Significantly differentially expressed segments are stored in [GEO:GSE44631] (G0/G1), [GEO:GSE44632] (G1/S), [GEO:GSE44633] (S/G2M) and [GEO:GSE44634] (G0/G2M).

Transcriptional activity under p53 expression can be accessed through [GEO:GSE43912] (defunct p53), [GEO:GSE43913] (p53 induced), while significant changes are available through [GEO:GSE43914] (p53 induced/defunct p53).

Transcriptional activity in response to STAT3 activation is available through [GEO:GSE44657] (INA-6 cells deprived from IL-6 for 13 h), [GEO:GSE44656] (restimulated after 1 h) and [GEO:GSE44658] (permanently cultured in IL-6). Significant changes of expression are stored in [GEO:GSE44659].

Microarray data used to estimate the FDR are deposited in [GEO:GSE29792] (G0/G1), [GEO:GSE29794] (p53 induced/defunct p53) and [GEO:GSE29793] (INA-6 cells deprived from IL-6 for 13 h and restimulated after 1 h) and to estimate differential expression in brain tumors in [GEO:GSE43911].

## Abbreviations

CAR: chromatin-associated RNA; CC: cell cycle; CDS: coding sequence; ChIP: chromatin immunoprecipitation; DE: significantly differentially expressed; DE-TAR: significantly differentially expressed TAR; DMEM: Dulbecco’s; modified Eagle’s medium; ENCODE: Encyclopedia of DNA Elements (ENCODE) Consortium; EST: expressed sequence tag; FCS: fetal calf serum; FDR: false discovery rate; GO: Gene ontology; H3K4me1: histone H3 lysine 4 mono-methylation; H3K4me3: histone H3 lysine 4 tri-methylation; H3K27ac: histone H3 lysine 27 acetylation; H3K27me3: histone H3 lysine 27 tri-methylation; H3K36me3: histone H3 lysine 36 tri-methylation; IgG: immunoglobulin G; IL-6: interleukin 6; kb: kilobase; lncRNA: long ncRNA; MB: million base pairs; ncRNA: non-protein-coding RNA; nt: nucleotide; PCR: polymerase chain reaction; polII: RNA polymerase II; qRT-PCR: quantitative real-time reverse transcriptase PCR; STAiR: STAT3-induced RNA; STAT3: signal transducer and activator of transcription-3; TAR: transcriptionally active region; UTR: untranslated region; vlincRNA: very long intergenic non-coding RNA.

## Competing interests

The authors declare no competing interests.

## Authors’ contributions

JH, KR, PFS and FH conceived the study and wrote the manuscript. KR, CO, AN and JH developed the software and performed the bioinformatics analyses. NH, CB, KBH, LB, KK, KE and FH designed and performed the STAT, cell-cycle and p53 experiments. PA and WK designed and performed the experiments on glioblastoma. All authors read, corrected and approved the final manuscript.

## Supplementary Material

Additional file 1 Supplemental material. Click here for file

Additional file 2 Online supplemental material containing BED formatted files of differentially expressed regions and macroRNAs, as well as CSV tables listing all proximal ncRNA-mRNA pairs regulated in astrocytoma. Click here for file
