# Supplementary material for: Cell cycle, oncogenic and tumor suppressor pathways regulate numerous long and macro non-protein-coding RNAs
Source: Genome Biol. 2014 Mar 4;15(3):R48. doi: 10.1186/gb-2014-15-3-r48 (PMC4054595; doi:10.1186/gb-2014-15-3-r48)
Supplement: Additional file 1 — Supplemental material. [file gb-2014-15-3-r48-S1.pdf]

Additional file:
Cell cycle, oncogenic and tumor suppressor pathways regulate numerous long and macro non-protein coding RNAs

February 6, 2014

Jörg Hackermüller[1,2,†,*], Kristin Reiche[1,2,†,*], Christian Otto[3,4], Nadine Hösler[5,4], Conny Blumert[5,6], Katja Brocke-Heidrich[5], Levin Böhlig[7], Anne Nitsche [3], Katharina Kasack[5,4,2], Peter Ahnert[4,8], Wolfgang Krupp[9], Kurt Engeland[7], Peter F. Stadler[3,2,10,11,12], Friedemann Horn[5,6]

1 Young Investigators Group Bioinformatics and Transcriptomics, Department Proteomics, Helmholtz Centre for Environmental Research – UFZ, Leipzig, Germany and Department for Computer Science, University of Leipzig, Leipzig, Germany

2 RNomics Group, Department of Diagnostics, Fraunhofer Institute for Cell Therapy and Immunology– IZI, Leipzig, Germany

3 Bioinformatics Group, Department of Computer Science, University of Leipzig, Leipzig, Germany

4 LIFE - Leipzig Research Center for Civilization Diseases, University of Leipzig, Leipzig, Germany

5 Institute of Clinical Immunology, University of Leipzig, Leipzig, Germany

6 Department of Diagnostics, Fraunhofer Institute for Cell Therapy and Immunology– IZI, Leipzig, Germany

7 Molecular Oncology, Medical School, University of Leipzig, Leipzig, Germany

8 Institute for Medical Informatics, Statistics and Epidemiology, University of Leipzig, Leipzig, Germany

9 Clinic of Neurosurgery, University of Leipzig, Leipzig, Germany

10 Max-Planck Institute for Mathematics in the Sciences, Leipzig, Germany

11 Santa Fe Institute, Santa Fe, New Mexico, United States of America

12 Department of Theoretical Chemistry, University of Vienna, Vienna, Austria

† These authors contributed equally.

∗ E-mail: joerg.hackermueller@ufz.de, kristin.reiche@ufz.de

Contents

1 STAT3 and P53 activation, and cell cycle synchronization **5**
- 1.1 STAT3 activation 5
- 1.2 P53 activation 5
- 1.3 Cell cycle synchronization 6

2 Transcriptionally active regions (TARs) **7**
- 2.1 Significantly expressed regions (`TileShuffle`) 7

3 Differentially expressed TARs (DE-TARs) **9**
- 3.1 Significantly differentially expressed regions (`TileShuffle`) 9
- 3.2 FDR estimation using the nONCOchip custom array 11
- 3.3 Independent 3'UTR expression 12
- 3.4 DE-TAR overlap with genomic annotation 14
- 3.5 *Bona fide* non-coding DE-TARs overlap with genomic annotation 17

4 Representation of TARs and DE-TARs on nONCOchip custom array **23**

5 MacroRNAs **24**
- 5.1 Evolutionary selection acting on STAiR1 compared to its neighbor protein-coding genes 39

6 Disease associated ncRNAs **41**
- 6.1 Clinical data of astrocytoma tumor subtypes 41
- 6.2 Content of the custom microarray - nONCOchip 42
- 6.3 Differential expression of astrocytoma of grade I versus aggressive states (grade III or IV) 43
- 6.4 *Bona fide* non-coding probes overlap with genomic annotation and DE-TARs . 44
- 6.5 Proximal ncRNA – mRNA pairs 48

7 Supplemental material and methods **54**
- 7.1 Primers for RT-PCR and ChIP 54
- 7.2 Annotation categories 55

List of Figures

Figure S1 STAT3 activation in INA-6 cells 5
Figure S2 P53 activation 5
Figure S3 Cell cycle synchronization of HFF cells 6
Figure S4 Transcriptionally active regions 7
Figure S5 Transcriptionally active *bona fide* non-coding regions in introns . . . 8
Figure S6 Differentially expressed TARs (DE-TARs) 9
Figure S7 Differentially expressed *bona fide* non-coding TARs in introns 10
Figure S8 FDR estimation using the nONCOchip custom array 11
Figure S9 Independent 3'UTR expression 12
Figure S10 DE-TAR overlap with genomic annotation 14
Figure S11 *Bona fide* non-coding DE-TARs in intergenic space overlap with genomic annotation 17
Figure S12 STAiR2 24
Figure S13 STAiR18 25
Figure S14 STAiR1 conserved elements 26
Figure S15 STAT3 binding site in STAiR1 27
Figure S16 Intron length variation man - dog 28
Figure S17 Macro RNA signal slopes 29
Figure S18 DE-Probes overlap with different annotation categories 44
Figure S19 Proximal ncRNA – mRNA pairs 48

List of Tables

Table S1 Transcriptionally active regions 7
Table S2 Differentially expressed regions (DE-TARs) 9
Table S3 Independent 3'UTR expression upon STAT3 activation 13
Table S4 DE-TAR overlap with protein coding genes. 15
Table S5 DE-TAR overlap with regulatory sites and epigenetically modified regions. 16
Table S6 Intergenic *bona fide* non-coding DE-TAR overlap with known non-coding RNAs. 18
Table S7 Intergenic *bona fide* non-coding DE-TAR overlap with regulatory sites and epigenetically modified regions. 19
Table S8 Intronic *bona fide* non-coding DE-TAR overlap with known non-coding RNAs. 20
Table S9 Intronic *bona fide* non-coding DE-TAR overlap with regulatory sites and epigenetically modified regions. 21
Table S10 LncRNAs with known function overlapped by *bona fide* non-coding DE-TARs 22
Table S11 Representation of TARs and DE-TARs on custom array 23
Table S12 DE-macroRNAs. 30
Table S13 DE-macroRNA overlap with known non-coding RNAs. 33
Table S14 DE-macroRNA overlap with regulatory sites and epigenetically modified regions. 35
Table S15 DE-macroRNA overlap with repeat regions. 37

Table S16 Evolutionary selection acting on STAiR1 compared to its neighbor protein-coding genes *SETBP1* and *SYT4*. 40
Table S17 Clinical, pathological, and immunohistochemical data of presented tumors. For the proliferative marker Ki67, percentage values were attributed to each case evaluating ten fields (x400 magnification). 41
Table S18 nONCOchip custom microarray 42
Table S19 Differential expression between brain tumors 43
Table S20 *Bona fide* non-coding DE-Probes overlap with known ncRNAs. 45
Table S21 *Bona fide* non-coding DE-Probes overlap with regulatory sites and epigenetically modified regions. 46
Table S22 *Bona fide* non-coding DE-Probes overlap with DE-TARs. 47
Table S23 Protein-coding genes proximal to *bona fide* non-coding DE-Probes and related to astrocytoma 49
Table S24 GO term enrichment for protein-coding genes proximal to *bona fide* non-coding DE-Probes in intergenic space. 50
Table S25 GO term enrichment for protein-coding genes with *bona fide* non-coding DE-Probes in introns. 52
Table S26 GO term enrichment for protein-coding genes with antisense *bona fide* non-coding DE-Probes. 53
Table S27 Primer sequences 54
Table S28 Overview of used annotation categories 57

1 STAT3 and P53 activation, and cell cycle synchronization

1.1 STAT3 activation

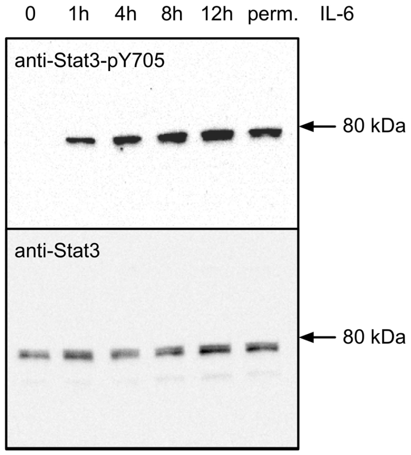

Figure S1: **STAT3 activation in INA-6 cells.** Human multiple myeloma INA-6 cells were permanently (perm.) cultivated in the presence of interleukin-6 (IL-6), or were restimulated by addition of IL-6 to the medium for the indicated periods after withdrawal from cytokine for 12 h. Subsequently, cells were lysed and proteins separated by SDS-polyacrylamide electrophoresis. STAT3 and activated STAT3 were detected by immunoblotting using antibodies to STAT3 (# 9132; Cell Signaling Technology, Dancers, MA, USA) and to STAT3 phosphorylated at tyrosine residue 705 (STAT3-pY705; # 9131; Cell Signaling Technology), respectively. STAT3 bands were visualized by chemiluminescence.

1.2 P53 activation

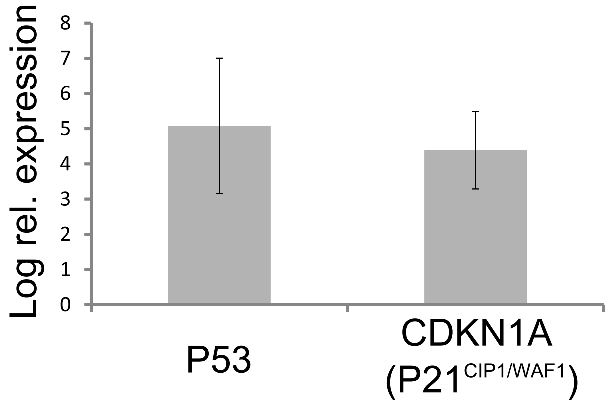

Figure S2: **P53 activation.** Regulation of p21$^{CIP1/WAF1}$ mRNA after induction of P53 for $6h$ in D53wt cells. mRNAs were measured by real time RT-PCR as previously described [1, 2]. Relative expression (log2 values) compared to expression in control cells is shown. GAPDH mRNA expression was used for normalization [3].

1.3 Cell cycle synchronization

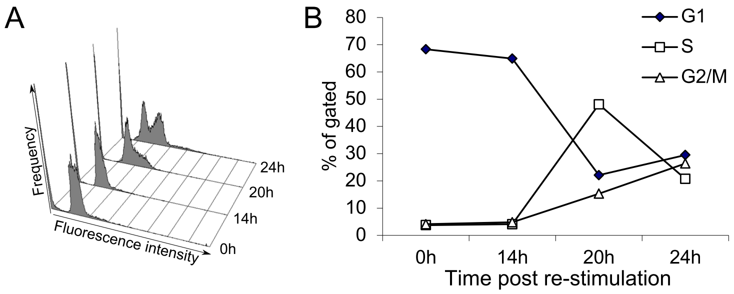

Figure S3: **Cell cycle distribution after starvation and restimulation of HFF cells.** Cells were starved for two days ($0h$) and restimulated with FCS containing media for the indicated time points [4]. (**A**) and (**B**) Cell cycle profiles were analysed by FACS and data evaluation was done with WinMDI [4].

2 Transcriptionally active regions (TARs)

2.1 Significantly expressed regions (`TileShuffle`)

					Bona fide non-coding			
Survey	Survey	all	CDS		intergenic		intronic	
CC	G0	17926188	8411574	(0.46)	1499907	(0.08)	5193188	(0.28)
	G1	21117194	10574212	(0.50)	1626415	(0.07)	5768644	(0.27)
	S	17610295	8771773	(0.49)	1405967	(0.07)	4632563	(0.26)
	G2	19682850	9948039	(0.50)	1494893	(0.07)	5186835	(0.26)
P53	Normal	22766570	10906780	(0.47)	1739895	(0.07)	7187124	(0.31)
	Induced	20635594	8318917	(0.40)	1750770	(0.08)	6430997	(0.31)
STAT3	Permanent cultured in IL-6	20949089	9824387	(0.46)	1927838	(0.09)	5844054	(0.27)
	13h withdrawal of IL-6	19656569	9101103	(0.46)	1745686	(0.08)	5613915	(0.28)
	1h IL-6 restimulation after 13h withdrawal	21467128	9848306	(0.45)	1952753	(0.09)	6313951	(0.29)

Table S1: **Transcriptionally active regions.** Overall number of significantly expressed nucleotides (`TileShuffle` $q < 0.05$) as well as number of significantly expressed nucleotides in protein-coding exons (`Gencode` v12, `Ensembl` genes, `UCSC` genes or `RefSeq` genes), in intergenic regions and in introns of known protein-coding genes.

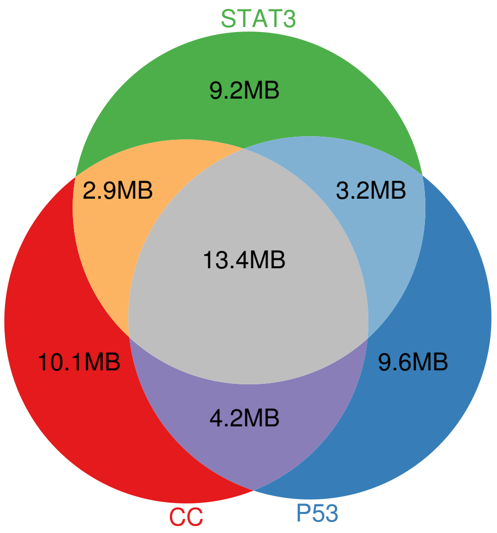

Figure S4: **Transcriptionally active regions.** Overall number of significantly expressed nucleotides (`TileShuffle` $q < 0.05$) and their nucleotide-wise overlaps between all three transcriptome-wide surveys.

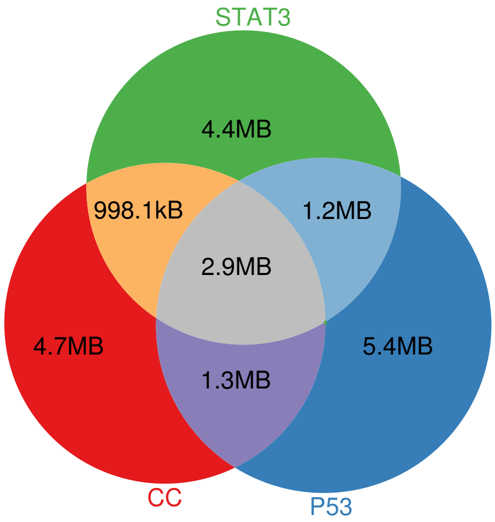

Figure S5: **Transcriptionally active *bona fide* non-coding regions in introns.** Number of significantly expressed nucleotides (`TileShuffle` $q < 0.05$) in introns of known protein-coding genes (`Gencode` v12, `Ensembl` genes, `UCSC` genes or `RefSeq` genes) and their nucleotide-wise overlaps between all three transcriptome-wide surveys.

3 Differentially expressed TARs (DE-TARs)

3.1 Significantly differentially expressed regions (TileShuffle)

					Bona fide non-coding			
Survey	Survey	all	CDS		intergenic		intronic	
CC	G0 vs. G1	1713009	966179	(0.56)	94602	(0.05)	491298	(0.28)
	G1 vs. S	34628	25015	(0.72)	3371	(0.09)	5004	(0.14)
	S vs. G2	9203	5008	(0.54)	1089	(0.11)	2776	(0.30)
	G2 vs. G0	1627902	931887	(0.57)	73824	(0.04)	432643	(0.26)
P53	Normal vs. induced	4094296	1596403	(0.38)	269710	(0.06)	1861615	(0.45)
STAT3	Permanent cultured in IL-6 vs. 1h restimulation	28582	3871	(0.13)	15204	(0.53)	8325	(0.29)
	Permanent cultured in IL-6 vs. 13h withdrawal	53409	4704	(0.08)	27500	(0.51)	19349	(0.36)
	1h IL-6 restimulation vs. 13h withdrawal	118045	14868	(0.12)	37422	(0.31)	60273	(0.51)

Table S2: **Differentially expressed regions (DE-TARs).** Overall number of significantly differentially expressed nucleotides (TileShuffle q $<$ 0.005) as well as number of significantly differentially expressed nucleotides in protein-coding exons (Gencode v12, Ensembl genes, UCSC genes or RefSeq genes), intergenic regions and in introns of known protein-coding genes.

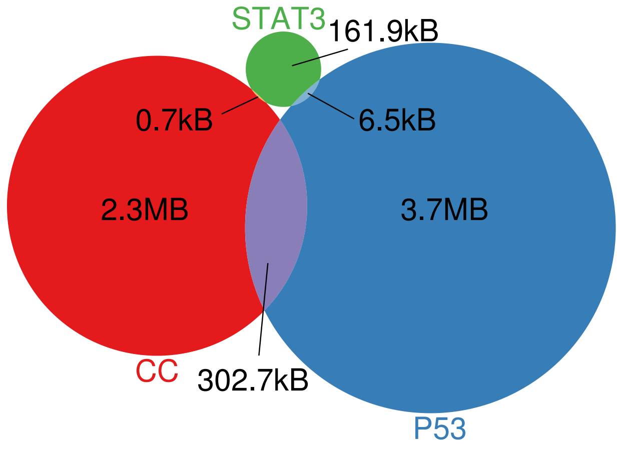

Figure S6: **Differentially expressed TARs (DE-TARs).** Overall number of significantly differentially expressed nucleotides (TileShuffle $q < 0.005$) and their nucleotide-wise overlaps between all three transcriptome-wide surveys.

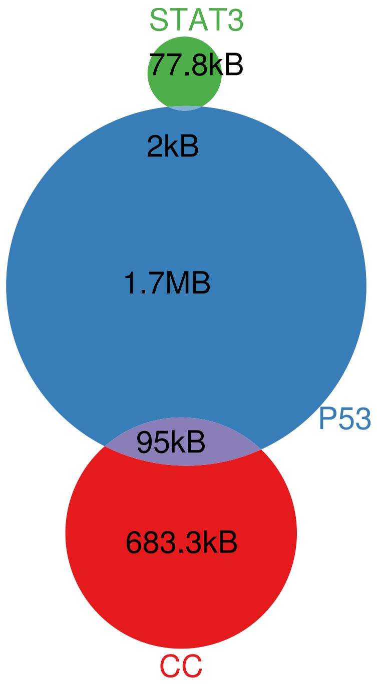

Figure S7: **Differentially expressed *bona fide* non-coding TARs in introns.** Number of significantly differentially expressed nucleotides (`TileShuffle` $q < 0.005$) in introns of known protein-coding genes (`Gencode` v12, `Ensembl` genes, `UCSC` genes or `RefSeq` genes) and their nucleotide-wise overlaps between all three transcriptome-wide surveys.

3.2 FDR estimation using the nONCOchip custom array

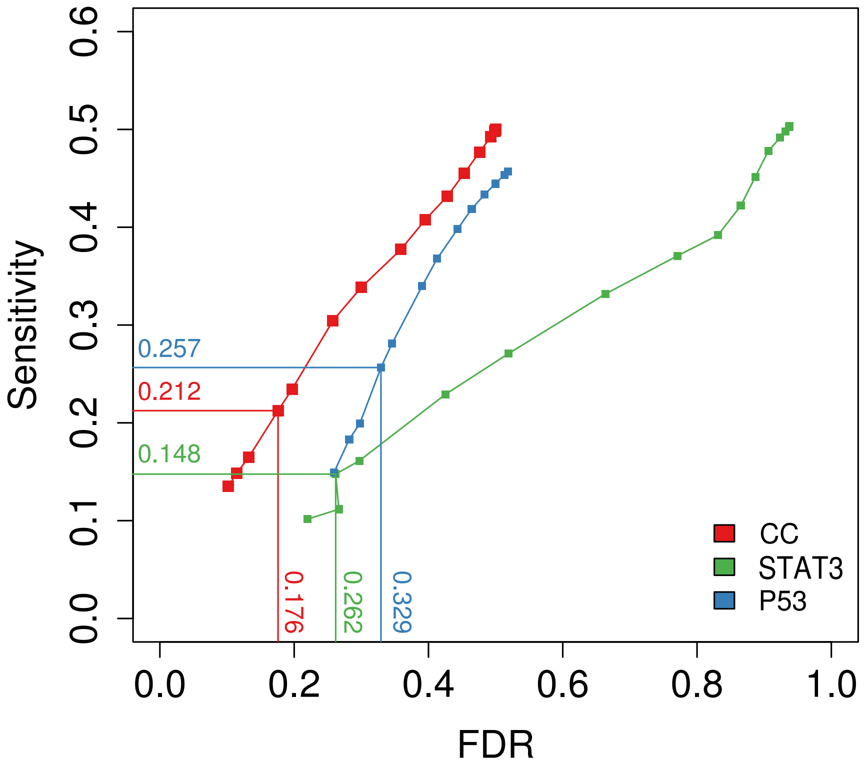

Figure S8: **FDR estimation using the nONCOchip custom array.** ROC curves showing sensitivity versus FDR of detecting differential expression with the tiling array approach. The nONCOchip custom array interrogates a significant subset of the differentially expressed intervals displayed in Figure 1D (see Supplemental Table S11 for detailed numbers). The nONCOchip was applied in biological triplicates to the following conditions: G0/G1 (CC), p53 induced/defunct p53 (p53), and INA-6 cells deprived from IL-6 for 13 hours/restimulated after 1h (STAT3). Subsequently, probes significantly differentially expressed were identified (see Materials and Methods). As already performed in [5], this set of RNAs was used as a "true" reference for estimating sensitivity and specificity of the tiling array experiment. Different points in the ROC curve are achieved by varying the q parameter of `TileShuffle` for differential expression analysis. Based on these data the q parameter has been set to $q = 0.005$ to give an overall FDR between 18% and 33% for all three data sets.

3.3 Independent 3'UTR expression

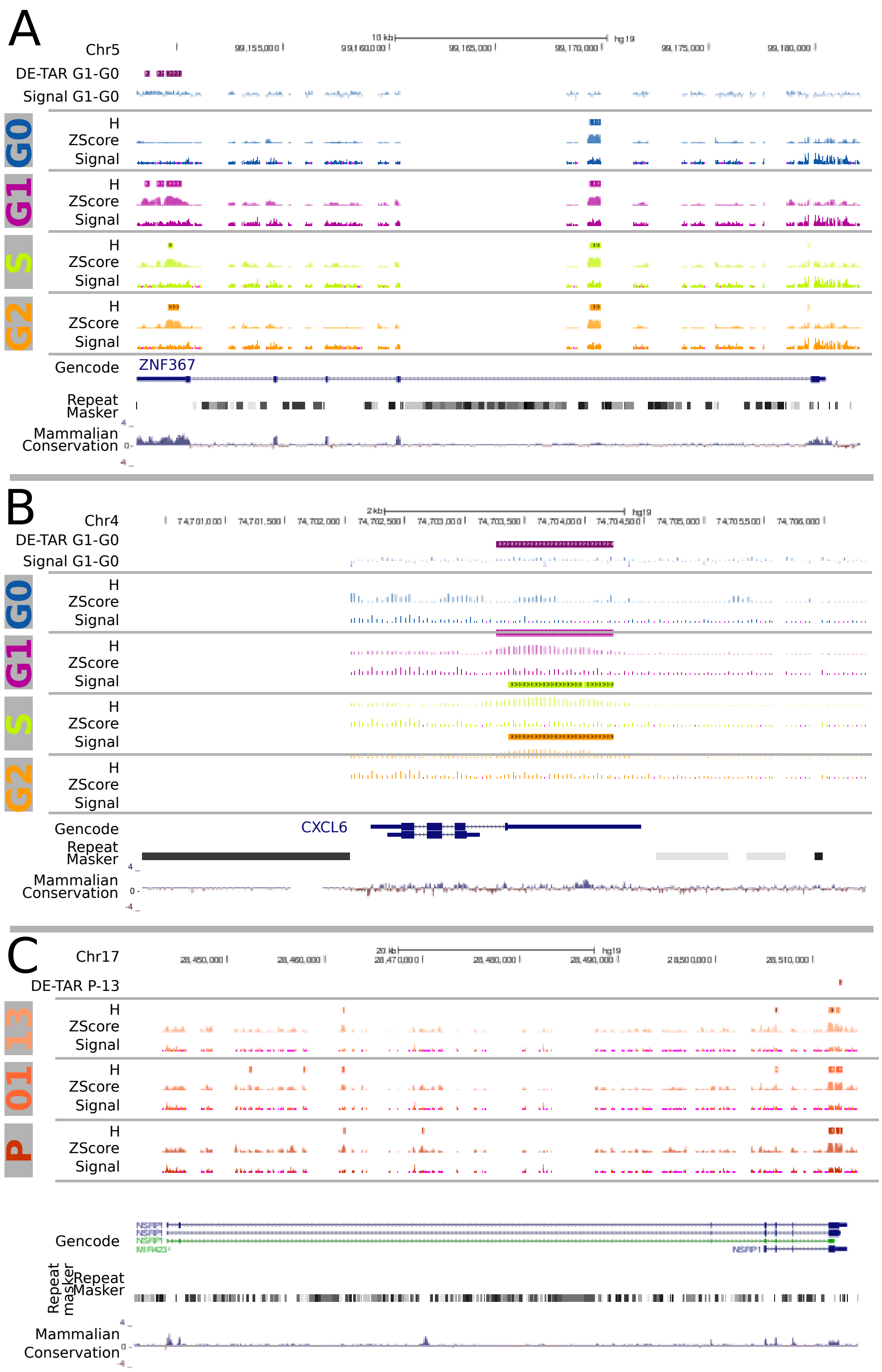

Figure S9: **Independent 3'UTR expression.** In approximately 400 cases, larger parts of a gene's 3'UTR are differentially expressed while the coding sequence is not. (**A**) shows the example ZNF 367 and (**B**) CXCL6. (**C**) CCDC55 (NSRP1) is a case where mainly the 3'UTR is expressed, but not differential.

Gene						Antisense Gene			
Name	Strand	DE-TARs				Name	Strand	DE-TARs	Expressed
		3'UTR	5'UTR	CDS	intron				
NSRP1	+	01-13		01-13					
SLC7A6	+	01-13				SLC7A6OS	-	01-13	13, 1, P
ICAM1	+	01-13, 13-P				CTD-2369P2	-	01-13, 13-P	1h, P
KDSR	-	01-13							

Table S3: **Independent 3'UTR expression upon STAT3 activation.** The observed discrepancy between non-enriched CDS and 5'-UTRs versus enriched 3'-UTRs in STAT3-DE-TARs might reflect an independent expression or processing of 3'-UTRs. Applying a stringent filtering scheme interrogating (i) 3'UTR must not overlap any protein-coding exon (CDS), (ii) 3'UTR must be covered by DE-TARs in at least 20% of nucleotides, and (iii) the average `TileShuffle` z-score for expression of the 3'UTR must be twice as high as the z-score for corresponding CDS exons, resulted in four candidates with 3'UTR regulation independent of the protein-coding gene. Two 3'UTRs overlap with annotated antisense transcripts (`Gencode` v12) which points to regulation of the antisense transcript rather than to independent 3'UTR expression.

3.4 DE-TAR overlap with genomic annotation

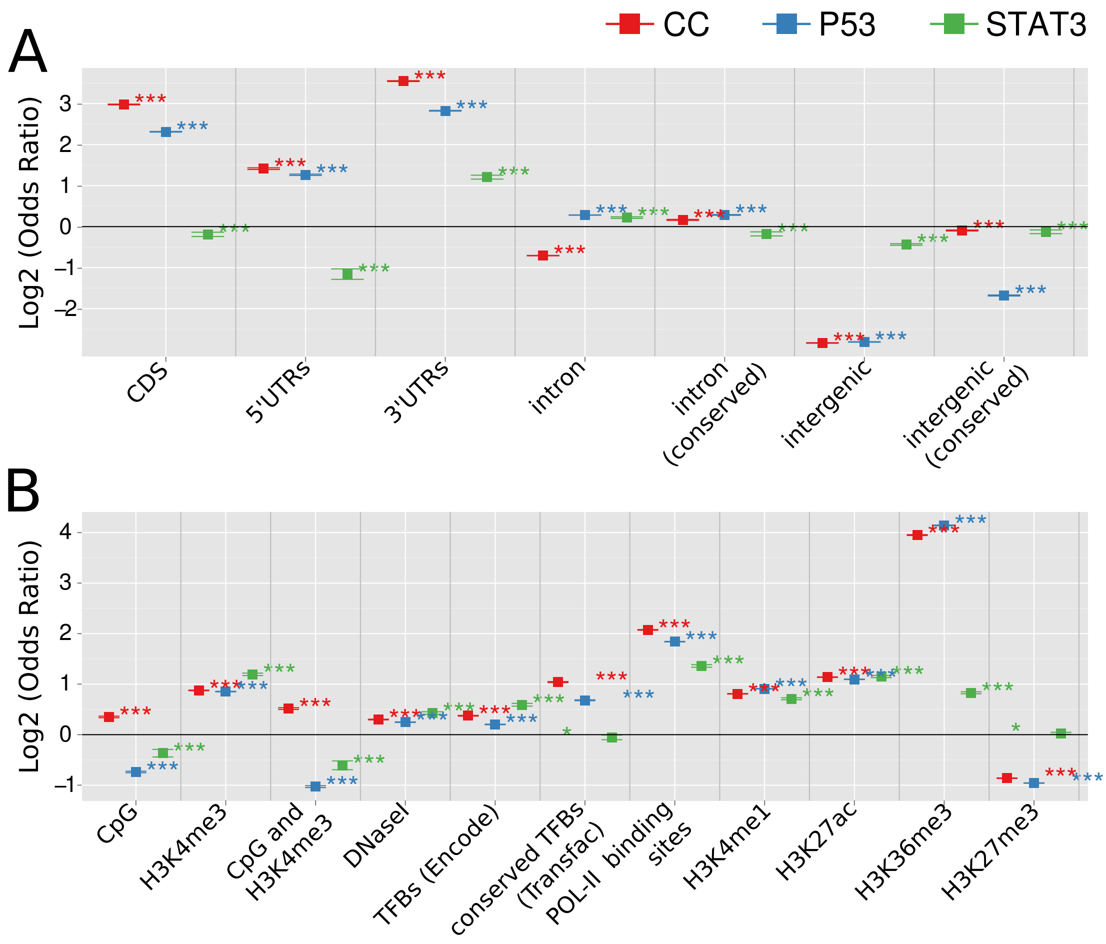

Figure S10: **DE-TAR overlap with genomic annotation.** Overlaps in nucleotides between DE-TARs and different annotation categories. Log2 transformed odds ratios and their 95% confidence interval for the respective annotation dataset are shown (annotations are described in detail in Supplemental Table S28). To assess the significance of the observed overlap, 100 lists containing random intervals from the genome controlling for repeat content and DE-TAR length are sampled. Odds ratios of observed versus randomized relative overlaps are calculated and tested by Fisher's exact test for significant enrichment or depletion. *** indicates a p-value $p < 0.001$ of the observed versus random nucleotide overlaps, ** a p-value $p < 0.01$, and * a p-value $p < 0.05$, respectively. Results are shown for DE-TARs which overlap (**A**) annotated protein coding genes versus intergenic space based on `Gencode` release v12, and (**B**) putative promoter regions, transcription factor binding sites, polII binding sites and epigenetically modified regions.

Table S4: **DE-TAR overlap with protein coding genes.** Overlaps in nucleotides between DE-TARs and annotated protein coding genes as well as intergenic space based on `Gencode` release v12. Annotation datasets are described in Supplemental Table S28. Overlaps are calculated by using the Bioconductor `genomeIntervals` package [6]. The significance of the observed overlap is assessed by generating a background (**BG**) of 100 random lists containing as much as random intervals from the human genome (hg19) than DE-TARs were identified. Random intervals are controlled for repeat content and DE-TAR length. Odds ratios of observed versus expected relative overlaps are calculated and tested by Fisher's exact test for significant enrichment or depletion (see Materials and Methods). Column heading **Annotation** indicates annotation datasets for which overlap is computed, and **Survey** if overlap is for cell cycle (CC), p53 or STAT3 (IL-6) pathway. Remaining columns indicate the results (**Odds ratio**, **P-value**, and 95% confidence interval for odds ratio - **95%CI**) and the data (**DE-TARs**: number of overlapping or non-overlapping nucleotides of DE-TARs with annotation; **BG**: average number of overlapping or non-overlapping nucleotides of random intervals with annotation among 100 random lists) of Fisher's exact test.

		Fisher's exact test			overlap		no overlap	
Annotation	Survey	Odds ratio	P-value	95% CI	DE-TARs	BG	DE-TARs	BG
CDS	CC	7.88	0.00e+00	[7.83, 7.93]	691846	113406.78	1993219	2573233.04
	P53	4.97	0.00e+00	[4.94, 4.99]	715884	167363.44	3378412	3922503.26
	IL6	0.88	1.01e-12	[0.85, 0.91]	5738	6529.13	163680	163331.07
intergenic	CC	0.14	0.00e+00	[0.14, 0.14]	279104	1217176.17	2405961	1469463.65
	P53	0.14	0.00e+00	[0.14, 0.14]	435732	1862021.38	3658564	2227845.32
	IL6	0.74	0.00e+00	[0.73, 0.75]	65464	78121.30	103954	91738.9
intergenic (conserved)	CC	0.94	5.07e-62	[0.93, 0.94]	132323	140866.19	2552742	2545773.63
	P53	0.31	0.00e+00	[0.31, 0.32]	68823	212292.36	4025473	3877574.34
	IL6	0.92	4.71e-08	[0.89, 0.95]	7841	8544.62	161577	161315.58
introns	CC	0.61	0.00e+00	[0.61, 0.61]	911084	1225247.42	1773981	1461392.4
	P53	1.22	0.00e+00	[1.21, 1.22]	2062161	1861055.49	2032135	2228811.21
	IL6	1.16	2.14e-108	[1.15, 1.18]	84059	77834.86	85359	92025.34
intron (conserved)	CC	1.12	1.45e-168	[1.11, 1.13]	131043	117640.97	2554022	2568998.85
	P53	1.22	0.00e+00	[1.21, 1.23]	213478	176729.33	3880818	3913137.37
	IL6	0.88	2.19e-12	[0.85, 0.92]	6371	7190.09	163047	162670.11
3′UTRs	CC	11.68	0.00e+00	[11.60,11.76]	772100	89692.14	1912965	2596947.68
	P53	7.08	0.00e+00	[7.04, 7.12]	794681	134497.59	3299615	3955369.11
	IL6	2.31	0.00e+00	[2.23, 2.38]	11695	5290.21	157723	164569.99
5′UTRs	CC	2.66	0.00e+00	[2.62, 2.70]	71971	27519.87	2613094	2659119.95
	P53	2.40	0.00e+00	[2.37, 2.43]	97974	41346.06	3996322	4048520.64
	IL6	0.45	1.15e-77	[0.41, 0.49]	730	1624.24	168688	168235.96

Table S5: **DE-TAR overlap with regulatory sites and epigenetically modified regions.** Overlaps in nucleotides between DE-TARs and putative promoter regions, transcription factor bindings sites and epigenetically modified regions. Annotation datasets are described in Supplemental Table S28. Overlaps are calculated by using the Bioconductor `genomeIntervals` package [6]. The significance of the observed overlap is assessed by generating a background (**BG**) of 100 random lists containing as much as random intervals from the human genome (hg19) than DE-TARs were identified. Random intervals are controlled for repeat content and DE-TAR length. Odds ratios of observed versus expected relative overlaps are calculated and tested by Fisher's exact test for significant enrichment or depletion (see Materials and Methods). Column heading **Annotation** indicates annotation datasets for which overlap is computed, and **Survey** if overlap is for cell cycle (CC), p53 or STAT3 (IL-6) pathway. Remaining columns indicate the results (**Odds ratio**, **P-value**, and 95% confidence interval for odds ratio - **95%CI**) and the data (**DE-TARs**: number of overlapping or non-overlapping nucleotides of DE-TARs with annotation; **BG**: average number of overlapping or non-overlapping nucleotides of random intervals with annotation among 100 random lists) of Fisher's exact test.

		Fisher's exact test			overlap		no overlap	
Annotation	Survey	Odds ratio	P-value	95% CI	DE-TARs	BG	DE-TARs	BG
CpG	CC	1.28	0.00e+00	[1.26, 1.29]	77215	60969.15	2607850	2625670.67
	P53	0.60	0.00e+00	[0.59, 0.61]	54228	89564.36	4040068	4000302.34
	IL6	0.78	1.58e-22	[0.74, 0.82]	2646	3405.58	166772	166454.62
CpG and H3K4me3	CC	1.43	0.00e+00	[1.41, 1.45]	66321	46740.30	2618744	2639899.52
	P53	0.49	0.00e+00	[0.48, 0.50]	34535	69762.53	4059761	4020104.17
	IL6	0.66	1.73e-43	[0.62, 0.70]	1782	2705.22	167636	167154.98
DNaseI	CC	1.23	0.00e+00	[1.22, 1.24]	503016	423909.11	2182049	2262730.71
	P53	1.19	0.00e+00	[1.18, 1.19]	741748	642687.34	3352548	3447179.36
	IL6	1.35	4.83e-238	[1.32, 1.37]	33823	26567.89	135595	143292.31
H3K27ac	CC	2.20	0.00e+00	[2.19, 2.21]	1047799	605185.53	1637266	2081454.29
	P53	2.14	0.00e+00	[2.13, 2.14]	1561428	915764.87	2532868	3174101.83
	IL6	2.22	0.00e+00	[2.19, 2.25]	65466	37524.99	103952	132335.21
H3k27me3	CC	0.55	0.00e+00	[0.55, 0.55]	1389000	1775246.93	1296065	911392.89
	P53	0.51	0.00e+00	[0.51, 0.52]	2039681	2693411.65	2054615	1396455.05
	IL6	1.02	1.25e-02	[1.00, 1.03]	112678	112282.79	56740	57577.41
H3K36me3	CC	15.46	0.00e+00	[15.38,15.55]	2519047	1330688.44	166018	1355951.38
	P53	17.66	0.00e+00	[17.58,17.74]	3869404	2018061.43	224892	2071805.27
	IL6	1.78	0.00e+00	[1.75, 1.80]	106903	83320.06	62515	86540.14
H3K4me1	CC	1.75	0.00e+00	[1.75, 1.76]	1659045	1289558.86	1026020	1397080.96
	P53	1.88	0.00e+00	[1.87, 1.88]	2587045	1953834.56	1507251	2136032.14
	IL6	1.64	0.00e+00	[1.61, 1.66]	102037	81646.76	67381	88213.44
H3K4me3	CC	1.84	0.00e+00	[1.83, 1.84]	739183	460680.09	1945882	2225959.73
	P53	1.81	0.00e+00	[1.80, 1.81]	1100823	691851.60	2993473	3398015.1
	IL6	2.29	0.00e+00	[2.25, 2.33]	52862	28101.65	116556	141758.55
POL-II	CC	4.21	0.00e+00	[4.19, 4.23]	1067581	364073.75	1617484	2322566.07
	P53	3.58	0.00e+00	[3.57, 3.59]	1457432	546846.35	2636864	3543020.35
	IL6	2.57	0.00e+00	[2.52, 2.61]	46853	22016.88	122565	147843.32
TFBs (Transfac)	CC	2.06	0.00e+00	[2.04, 2.07]	260450	133371.08	2424615	2553268.74
	P53	1.60	0.00e+00	[1.59, 1.61]	312822	200721.49	3781474	3889145.21
	IL6	0.96	2.30e-02	[0.93, 0.99]	7804	8105.31	161614	161754.89
TFBs (Encode)	CC	1.30	0.00e+00	[1.29, 1.30]	422704	338458.03	2262361	2348181.79
	P53	1.15	0.00e+00	[1.15, 1.15]	580090	513351.52	3514206	3576515.18
	IL6	1.51	0.00e+00	[1.48, 1.54]	29775	21050.02	139643	148810.18

3.5 *Bona fide* non-coding DE-TARs overlap with genomic annotation

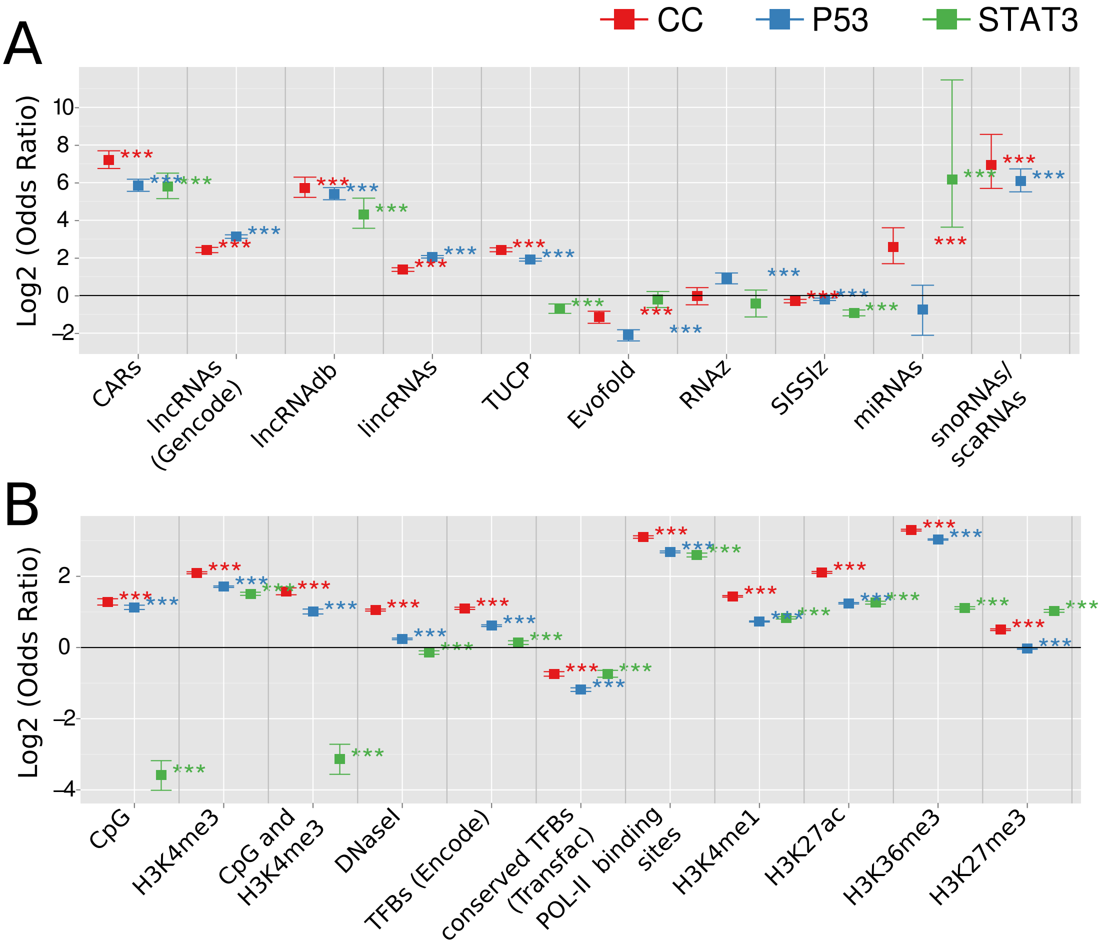

Figure S11: ***Bona fide* non-coding DE-TARs in intergenic space overlap with genomic annotation.** Overlaps in nucleotides between *bona fide* non-coding DE-TARs and different annotation categories. Log2 transformed odds ratios and their 95% confidence interval for the respective annotation dataset are shown (annotations are described in detail in Supplemental Table S28). To assess the significance of the observed overlap, 100 lists containing random intervals from the genome controlling for repeat content and DE-TAR length are sampled. Odds ratios of observed versus randomized relative overlaps are calculated and tested by Fisher's exact test for significant enrichment or depletion. *** indicates a p-value $p < 0.001$ of the observed versus random nucleotide overlaps, ** a p-value $p < 0.01$, and * a p-value $p < 0.05$, respectively. Results are shown for *bona fide* non-coding DE-TARs in intergenic space which overlap (**A**) with several classes of experimentally verified and predicted ncRNAs, and (**B**) putative promoter regions, transcription factor binding sites, polII binding sites and epigenetically modified regions.

Table S6: **Intergenic *bona fide* non-coding DE-TAR overlap with known non-coding RNAs.** Overlaps in nucleotides between intergenic *bona fide* non-coding DE-TARs and known non-coding RNAs. Annotation datasets are described in Supplemental Table S28. Overlaps are calculated by using the Bioconductor `genomeIntervals` package [6]. The significance of the observed overlap is assessed by generating a background (**BG**) of 100 random lists containing as much as random intervals from the human genome (hg19) than DE-TARs were identified. Random intervals are controlled for repeat content and DE-TAR length. Odds ratios of observed versus expected relative overlaps are calculated and tested by Fisher's exact test for significant enrichment or depletion (see Materials and Methods). Column heading **Annotation** indicates annotation datasets for which overlap is computed, and **Survey** if overlap is for cell cycle (CC), p53 or STAT3 (IL-6) pathway. Remaining columns indicate the results (**Odds ratio**, **P-value**, and 95% confidence interval for odds ratio - **95%CI**) and the data (**DE-TARs**: number of overlapping or non-overlapping nucleotides of DE-TARs with annotation; **BG**: average number of overlapping or non-overlapping nucleotides of random intervals with annotation among 100 random lists) of Fisher's exact test.

		Fisher's exact test			overlap		no overlap	
Annotation	Survey	Odds ratio	P-value	95% CI	DE-TARs	BG	DE-TARs	BG
CARs (intergenic)	CC	146.66	0.00e+00	[107.68, 206.83]	5641	39.84	138731	144152.03
	P53	57.71	0.00e+00	[46.43, 72.57]	4661	82.08	265049	269077.68
	IL6	55.08	3.40e-293	[35.49, 90.81]	1094	20.16	61875	62310.83
Evofold	CC	0.45	6.40e-14	[0.36, 0.56]	119	263.81	144253	143928.06
	P53	0.23	1.74e-58	[0.19, 0.28]	117	504.17	269593	268655.59
	IL6	0.86	3.47e-01	[0.64, 1.16]	89	102.17	62880	62228.82
lincRNAs	CC	2.60	3.82e-194	[2.43, 2.78]	3144	1224.27	141228	142967.6
	P53	4.16	0.00e+00	[3.98, 4.36]	9302	2289.35	260408	266870.41
	IL6	0.00	2.21e-164	[0.00, 0.01]	0	537.94	62969	61793.05
lncRNAdb	CC	52.97	0.00e+00	[37.18, 78.33]	1626	30.57	142746	144161.3
	P53	42.35	0.00e+00	[34.07, 53.24]	3519	83.87	266191	269075.89
	IL6	19.96	4.39e-70	[11.91, 36.10]	301	15.01	62668	62315.98
lncRNAs (Gencode)	CC	5.34	0.00e+00	[4.85, 5.89]	2629	499.23	141743	143692.64
	P53	8.77	0.00e+00	[8.22, 9.36]	8894	1042.81	260816	268116.95
	IL6	0.00	4.80e-69	[0.00, 0.02]	0	225.07	62969	62105.92
RNAz	CC	0.97	8.77e-01	[0.71, 1.34]	82	84.02	144290	144107.85
	P53	1.87	1.17e-10	[1.54, 2.29]	291	155.21	269419	269004.55
	IL6	0.75	2.40e-01	[0.45, 1.22]	31	41.33	62938	62289.66
miRNAs	CC	5.99	1.39e-11	[3.23, 12.14]	72	11.94	144300	144179.93
	P53	0.60	2.29e-01	[0.23, 1.46]	9	15.43	269701	269144.33
	IL6	71.35	1.64e-20	[12.40,2815.52]	72	0.78	62897	62330.21
snoRNAs or scaRNAs	CC	121.55	9.49e-173	[51.72, 378.44]	606	4.54	143766	144187.33
	P53	67.97	0.00e+00	[45.56, 106.17]	1625	23.57	268085	269136.19
	IL6	0.00	3.75e-03	[0.00, 0.58]	0	7.76	62969	62323.23
SISSIz	CC	0.81	1.28e-10	[0.76, 0.87]	1804	2205.88	142568	141985.99
	P53	0.87	7.34e-10	[0.83, 0.91]	3694	4229.90	266016	264929.86
	IL6	0.53	2.06e-33	[0.47, 0.59]	526	980.20	62443	61350.79
TUCP	CC	5.39	0.00e+00	[5.02, 5.80]	4709	896.27	139663	143295.6
	P53	3.74	0.00e+00	[3.55, 3.94]	7125	1939.15	262585	267220.61
	IL6	0.62	2.07e-08	[0.52, 0.73]	216	346.11	62753	61984.88

Table S7: **Intergenic *bona fide* non-coding DE-TAR overlap with regulatory sites and epigenetically modified regions.** Overlaps in nucleotides between intergenic *bona fide* non-coding DE-TARs and putative promoter regions, transcription factor bindings sites and epigenetically modified regions. Annotation datasets are described in Supplemental Table S28. Overlaps are calculated by using the Bioconductor `genomeIntervals` package [6]. The significance of the observed overlap is assessed by generating a background (**BG**) of 100 random lists containing as much as random intervals from the human genome (hg19) than DE-TARs were identified. Random intervals are controlled for repeat content and DE-TAR length. Odds ratios of observed versus expected relative overlaps are calculated and tested by Fisher's exact test for significant enrichment or depletion (see Materials and Methods). Column heading **Annotation** indicates annotation datasets for which overlap is computed, and **Survey** if overlap is for cell cycle (CC), p53 or STAT3 (IL-6) pathway. Remaining columns indicate the results (**Odds ratio**, **P-value**, and 95% confidence interval for odds ratio - **95%CI**) and the data (**DE-TARs**: number of overlapping or non-overlapping nucleotides of DE-TARs with annotation; **BG**: average number of overlapping or non-overlapping nucleotides of random intervals with annotation among 100 random lists) of Fisher's exact test.

		Fisher's exact test			overlap		no overlap	
Annotation	Survey	Odds ratio	P-value	95% CI	DE-TARs	BG	DE-TARs	BG
CpG	CC	2.43	2.30e-198	[2.29, 2.59]	3603	1501.97	140769	142689.9
	P53	2.19	0.00e+00	[2.10, 2.28]	7121	3296.56	262589	265863.2
	IL6	0.08	1.19e-128	[0.06, 0.11]	54	633.60	62915	61697.39
CpG and H3K4me3	CC	2.99	9.87e-257	[2.79, 3.20]	3401	1154.95	140971	143036.92
	P53	2.02	1.95e-192	[1.92, 2.12]	5110	2553.57	264600	266606.19
	IL6	0.11	1.43e-83	[0.09, 0.15]	54	463.84	62915	61867.15
DNaseI	CC	2.07	0.00e+00	[2.03, 2.12]	33570	18378.60	110802	125813.27
	P53	1.18	5.41e-100	[1.17, 1.20]	39834	34389.84	229876	234769.92
	IL6	0.91	1.30e-08	[0.88, 0.94]	7416	7999.10	55553	54331.89
H3K27ac	CC	4.32	0.00e+00	[4.24, 4.40]	61253	21017.48	83119	123174.39
	P53	2.37	0.00e+00	[2.34, 2.40]	77119	38931.89	192591	230227.87
	IL6	2.40	0.00e+00	[2.33, 2.46]	18648	9313.25	44321	53017.74
H3k27me3	CC	1.41	0.00e+00	[1.39, 1.44]	111922	102259.12	32450	41932.75
	P53	0.98	1.88e-04	[0.97, 0.99]	189126	189991.37	80584	79168.39
	IL6	2.04	0.00e+00	[1.99, 2.10]	52672	44553.31	10297	17777.68
H3K36me3	CC	9.83	0.00e+00	[9.67,10.00]	111081	36530.86	33291	107661.01
	P53	8.19	0.00e+00	[8.09, 8.29]	198477	68313.26	71233	200846.5
	IL6	2.16	0.00e+00	[2.11, 2.21]	27053	16104.44	35916	46226.55
H3K4me1	CC	2.71	0.00e+00	[2.67, 2.75]	90407	55095.49	53965	89096.38
	P53	1.66	0.00e+00	[1.64, 1.68]	136575	102780.36	133135	166379.4
	IL6	1.78	0.00e+00	[1.74, 1.82]	33473	24250.92	29496	38080.07
H3K4me3	CC	4.29	0.00e+00	[4.20, 4.37]	49607	15688.67	94765	128503.2
	P53	3.27	0.00e+00	[3.22, 3.32]	77639	29630.58	192071	239529.18
	IL6	2.85	0.00e+00	[2.76, 2.94]	16635	6978.55	46334	55352.44
POL-II	CC	8.60	0.00e+00	[8.39, 8.81]	51059	8626.30	93313	135565.57
	P53	6.44	0.00e+00	[6.32, 6.56]	78295	16078.41	191415	253081.35
	IL6	6.06	0.00e+00	[5.83, 6.29]	17439	3706.56	45530	58624.43
TFBs (Transfac)	CC	0.60	3.17e-120	[0.57, 0.62]	3305	5436.41	141067	138755.46
	P53	0.44	0.00e+00	[0.42, 0.46]	4588	10179.23	265122	258980.53
	IL6	0.60	1.39e-52	[0.56, 0.64]	1441	2343.18	61528	59987.81
TFBs (Encode)	CC	2.15	0.00e+00	[2.10, 2.19]	27198	14079.38	117174	130112.49
	P53	1.53	0.00e+00	[1.50, 1.56]	38275	26262.47	231435	242897.29
	IL6	1.10	2.38e-07	[1.06, 1.14]	6903	6273.74	56066	56057.25

Table S8: **Intronic *bona fide* non-coding DE-TAR overlap with known non-coding RNAs.** Overlaps in nucleotides between intronic *bona fide* non-coding DE-TARs and known non-coding RNAs. Annotation datasets are described in Supplemental Table S28. Overlaps are calculated by using the Bioconductor `genomeIntervals` package [6]. The significance of the observed overlap is assessed by generating a background (**BG**) of 100 random lists containing as much as random intervals from the human genome (hg19) than DE-TARs were identified. Random intervals are controlled for repeat content and DE-TAR length. Odds ratios of observed versus expected relative overlaps are calculated and tested by Fisher's exact test for significant enrichment or depletion (see Materials and Methods). Column heading **Annotation** indicates annotation datasets for which overlap is computed, and **Survey** if overlap is for cell cycle (CC), p53 or STAT3 (IL-6) pathway. Remaining columns indicate the results (**Odds ratio**, **P-value**, and 95% confidence interval for odds ratio - **95%CI**) and the data (**DE-TARs**: number of overlapping or non-overlapping nucleotides of DE-TARs with annotation; **BG**: average number of overlapping or non-overlapping nucleotides of random intervals with annotation among 100 random lists) of Fisher's exact test.

		Fisher's exact test			overlap		no overlap	
Annotation	Survey	Odds ratio	P-value	95% CI	DE-TARs	BG	DE-TARs	BG
CARs (intron)	CC	63.06	0.00e+00	[60.36,65.98]	107043	1965.59	662053	767356.04
	P53	22.76	0.00e+00	[22.13,23.42]	111823	5198.03	1749792	1850772.97
	IL6	1.20	4.74e-02	[1.00, 1.44]	270	223.60	78835	78384.63
Evofold	CC	0.43	3.96e-55	[0.38, 0.48]	445	1039.95	768651	768281.68
	P53	0.71	7.23e-35	[0.67, 0.75]	2158	3035.82	1859457	1852935.18
	IL6	0.82	1.80e-01	[0.62, 1.09]	91	110.41	79014	78497.82
lincRNAs	CC	1.96	3.74e-28	[1.73, 2.22]	750	382.52	768346	768939.11
	P53	0.93	1.09e-01	[0.85, 1.02]	896	963.21	1860719	1855007.79
	IL6	0.00	3.21e-12	[0.00, 0.10]	0	38.35	79105	78569.88
lncRNAdb	CC	1.96	3.94e-13	[1.62, 2.38]	327	166.89	768769	769154.74
	P53	10.18	0.00e+00	[9.14,11.37]	3709	363.73	1857906	1855607.27
	IL6	0.00	3.81e-03	[0.00, 0.58]	0	8.27	79105	78599.96
PINs	CC	6.91	0.00e+00	[6.59, 7.25]	13656	2007.16	755440	767314.47
	P53	4.77	0.00e+00	[4.61, 4.94]	18935	3986.98	1842680	1851984.02
	IL6	2.04	5.24e-17	[1.71, 2.43]	399	195.14	78706	78413.09
RNAz	CC	0.45	2.19e-28	[0.39, 0.53]	265	583.72	768831	768737.91
	P53	1.14	1.16e-03	[1.05, 1.23]	1409	1236.88	1860206	1854734.12
	IL6	0.65	5.90e-02	[0.40, 1.03]	32	49.36	79073	78558.87
miRNAs	CC	1.47	6.42e-02	[0.96, 2.29]	56	38.27	769040	769283.36
	P53	0.49	7.34e-06	[0.35, 0.68]	56	113.53	1861559	1855857.47
	IL6	0.00	1.53e-02	[0.00, 0.84]	0	5.63	79105	78602.6
snoRNAs or scaRNAs	CC	3.22	5.77e-09	[2.08, 5.11]	90	27.61	769006	769294.02
	P53	12.02	4.35e-138	[9.17,16.04]	687	56.68	1860928	1855914.32
	IL6	0.00	1.24e-01	[0.00, 2.41]	0	2.93	79105	78605.3
SISSIz	CC	0.74	1.67e-115	[0.72, 0.76]	10110	13598.05	758986	755723.58
	P53	0.73	0.00e+00	[0.71, 0.74]	24010	32823.03	1837605	1823147.97
	IL6	1.13	8.43e-04	[1.05, 1.22]	1560	1371.42	77545	77236.81
TINs	CC	1.93	0.00e+00	[1.89, 1.98]	24770	13021.62	744326	756300.01
	P53	2.29	0.00e+00	[2.26, 2.32]	73035	32556.82	1788580	1823414.18
	IL6	1.00	9.85e-01	[0.93, 1.08]	1396	1385.97	77709	77222.26
TUCP	CC	1.30	8.52e-04	[1.11, 1.52]	376	289.78	768720	769031.85
	P53	0.05	4.24e-148	[0.03, 0.07]	30	630.87	1861585	1855340.13
	IL6	0.00	3.40e-09	[0.00, 0.14]	0	28.37	79105	78579.86

Table S9: **Intronic *bona fide* non-coding DE-TAR overlap with regulatory sites and epigenetically modified regions.** Overlaps in nucleotides between intronic *bona fide* non-coding DE-TARs and putative promoter regions, transcription factor bindings sites and epigenetically modified regions. Annotation datasets are described in Supplemental Table S28. Overlaps are calculated by using the Bioconductor `genomeIntervals` package [6]. The significance of the observed overlap is assessed by generating a background (**BG**) of 100 random lists containing as much as random intervals from the human genome (hg19) than DE-TARs were identified. Random intervals are controlled for repeat content and DE-TAR length. Odds ratios of observed versus expected relative overlaps are calculated and tested by Fisher's exact test for significant enrichment or depletion (see Materials and Methods). Column heading **Annotation** indicates annotation datasets for which overlap is computed, and **Survey** if overlap is for cell cycle (CC), p53 or STAT3 (IL-6) pathway. Remaining columns indicate the results (**Odds ratio**, **P-value**, and 95% confidence interval for odds ratio - **95%CI**) and the data (**DE-TARs**: number of overlapping or non-overlapping nucleotides of DE-TARs with annotation; **BG**: average number of overlapping or non-overlapping nucleotides of random intervals with annotation among 100 random lists) of Fisher's exact test.

		Fisher's exact test			overlap		no overlap	
Annotation	Survey	Odds ratio	P-value	95% CI	DE-TARs	BG	DE-TARs	BG
CpG	CC	0.70	6.50e-92	[0.68, 0.73]	5511	7847.24	763585	761474.39
	P53	0.69	2.43e-236	[0.68, 0.71]	13631	19515.52	1847984	1836455.48
	IL6	0.65	3.37e-15	[0.58, 0.72]	537	821.20	78568	77787.03
CpG and H3K4me3	CC	0.79	4.12e-35	[0.76, 0.82]	4946	6250.06	764150	763071.57
	P53	0.42	0.00e+00	[0.41, 0.43]	6304	14947.42	1855311	1841023.58
	IL6	0.00	5.82e-197	[0.00, 0.01]	0	647.30	79105	77960.93
DNaseI	CC	1.36	0.00e+00	[1.35, 1.37]	167249	130622.10	601847	638699.53
	P53	1.28	0.00e+00	[1.27, 1.29]	393312	320879.54	1468303	1535091.46
	IL6	1.44	2.47e-189	[1.41, 1.48]	18609	13804.38	60496	64803.85
H3K27ac	CC	2.45	0.00e+00	[2.43, 2.47]	364853	207181.01	404243	562140.62
	P53	2.27	0.00e+00	[2.26, 2.27]	851864	503676.57	1009751	1352294.43
	IL6	2.05	0.00e+00	[2.01, 2.09]	34893	21844.10	44212	56764.13
H3k27me3	CC	0.78	0.00e+00	[0.78, 0.79]	443341	488349.40	325755	280972.23
	P53	0.73	0.00e+00	[0.73, 0.73]	1048757	1185976.83	812858	669994.17
	IL6	0.67	0.00e+00	[0.66, 0.68]	43241	50519.10	35864	28089.13
H3K36me3	CC	10.32	0.00e+00	[10.20,10.44]	731877	504529.61	37219	264792.02
	P53	14.91	0.00e+00	[14.78,15.04]	1797078	1208756.46	64537	647214.54
	IL6	1.22	3.47e-77	[1.20, 1.25]	55265	51475.71	23840	27132.52
H3K4me1	CC	2.08	0.00e+00	[2.07, 2.10]	546933	416652.84	222163	352668.79
	P53	2.25	0.00e+00	[2.24, 2.26]	1363174	1017808.49	498441	838162.51
	IL6	1.37	7.52e-205	[1.34, 1.40]	49582	43325.43	29523	35282.8
H3K4me3	CC	2.02	0.00e+00	[2.01, 2.04]	243866	143566.92	525230	625754.71
	P53	1.97	0.00e+00	[1.96, 1.98]	592117	355839.29	1269498	1500131.71
	IL6	2.06	0.00e+00	[2.01, 2.10]	26043	15144.67	53062	63463.56
POL-II	CC	3.76	0.00e+00	[3.73, 3.79]	292669	107961.14	476427	661360.49
	P53	2.96	0.00e+00	[2.95, 2.98]	606671	260196	1254944	1595775
	IL6	1.52	9.20e-213	[1.48, 1.56]	15934	11193.32	63171	67414.91
TFBs (Transfac)	CC	0.86	2.06e-65	[0.84, 0.87]	23024	26779.83	746072	742541.8
	P53	0.85	3.36e-168	[0.85, 0.86]	59494	69032.83	1802121	1786938.17
	IL6	1.06	4.23e-02	[1.00, 1.11]	3034	2861.91	76071	75746.32
TFBs (Encode)	CC	1.24	0.00e+00	[1.23, 1.25]	117394	97453.82	651702	671867.81
	P53	1.21	0.00e+00	[1.20, 1.22]	281104	237620.77	1580511	1618350.23
	IL6	1.55	1.81e-218	[1.51, 1.59]	14893	10239.14	64212	68369.09

Table S10: **LncRNAs with known function overlapped by *bona fide* non-coding DE-TARs.**

Survey	LncRNA	Role	Reference
CC, STAT3	MIAT1	associated with myocardial infarction; modulates Oct4 levels in embryonal stem cells	[7]
CC	MALAT1	associated with metastasis in lung adenocarcinoma	[8, 9]
CC	MEG3	tumor suppressor expressed imprinted locus; frequently downregulated in primary tumors	[10]
P53	Cyrano	antisense to tumor drug target OIP5; interference with neuronal development in zebra fish	[11, 12]
P53	ZNFX1-AS1	potential marker for breast cancer	[13]
P53	HOTAIRM1	regulator of HOXA1 cluster gene expression in myeologenesis	[14]
P53	HOTTIP	activator of HOXA1 gene expression acting by promoting H3K4 trimethylation	[15]
P53	GAS5	pleitropic, associated with growth arrest; some but not all transcript variants have been found to induce apoptosis	[16, 17]
STAT3	TMEVPG1	regulator of interferon gamma-expression in T cells	[18, 19]

4 Representation of TARs and DE-TARs on nONCOchip custom array

	Number of TARs	Fraction of TARs
	TARs	
CC	15816	0.09
P53	16673	0.09
STAT3	13283	0.08
	DE-TARs	
CC	4336	0.27
P53	6351	0.25
STAT3	385	0.31

Table S11: **Representation of TARs and DE-TARs on custom microarray.** Number and fraction of significantly expressed tiling array regions (TARs) and significantly differentially expressed tiling array regions (DE-TARs) which overlap at least one probe on the custom microarray. Each probe overlapping to at least 95% (i.e. 57 nucleotides) with an tiling array region is counted.

5 MacroRNAs

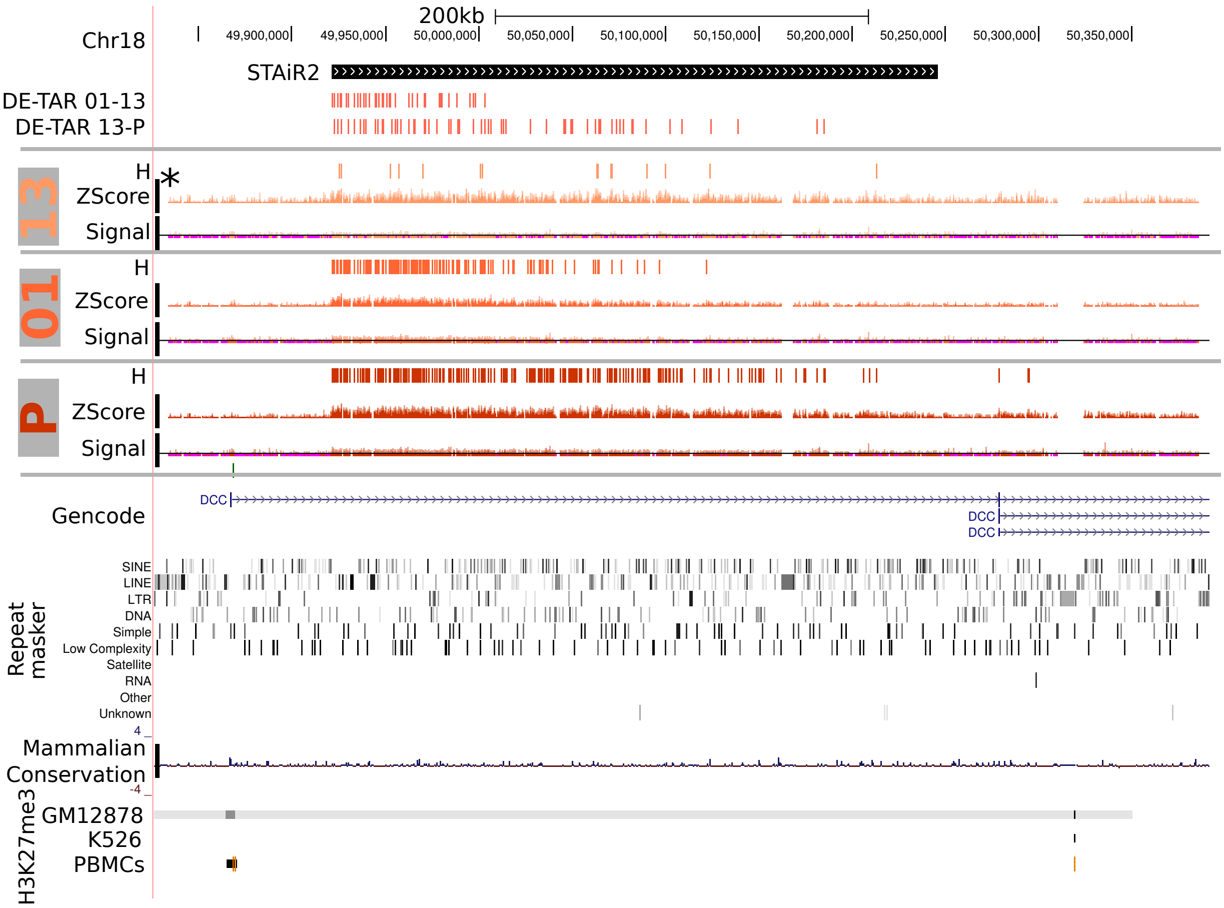

Figure S12: **STAT3-induced RNA 2 (STAiR2).** INA6 cells were cultured in absence of IL-6 for $13h$ (13), restimulated for $1h$ (01), or permanently grown in presence of IL-6 (P) and RNA expression was analyzed using tiling arrays. `TileShuffle` identified strong differential expression between P and 13 states over a range of up to $200kb$ (DE P-13), between 01 and 13 a similarly regulated but shorter region was identified (DE 01-13). This putative macro RNA is located in the first intron of the protein coding gene DCC (deleted in colorectal carcinoma), a tumor suppressor that is frequently found mutated or downregulated in colorectal and oesophagal cancer. (*) Wiggle track scale bars indicate y-axis scales of (6,16), (0,10), (-3.5,3.5), and (-4,4) for signal, z-score, differential signal, and conservation, respectively.

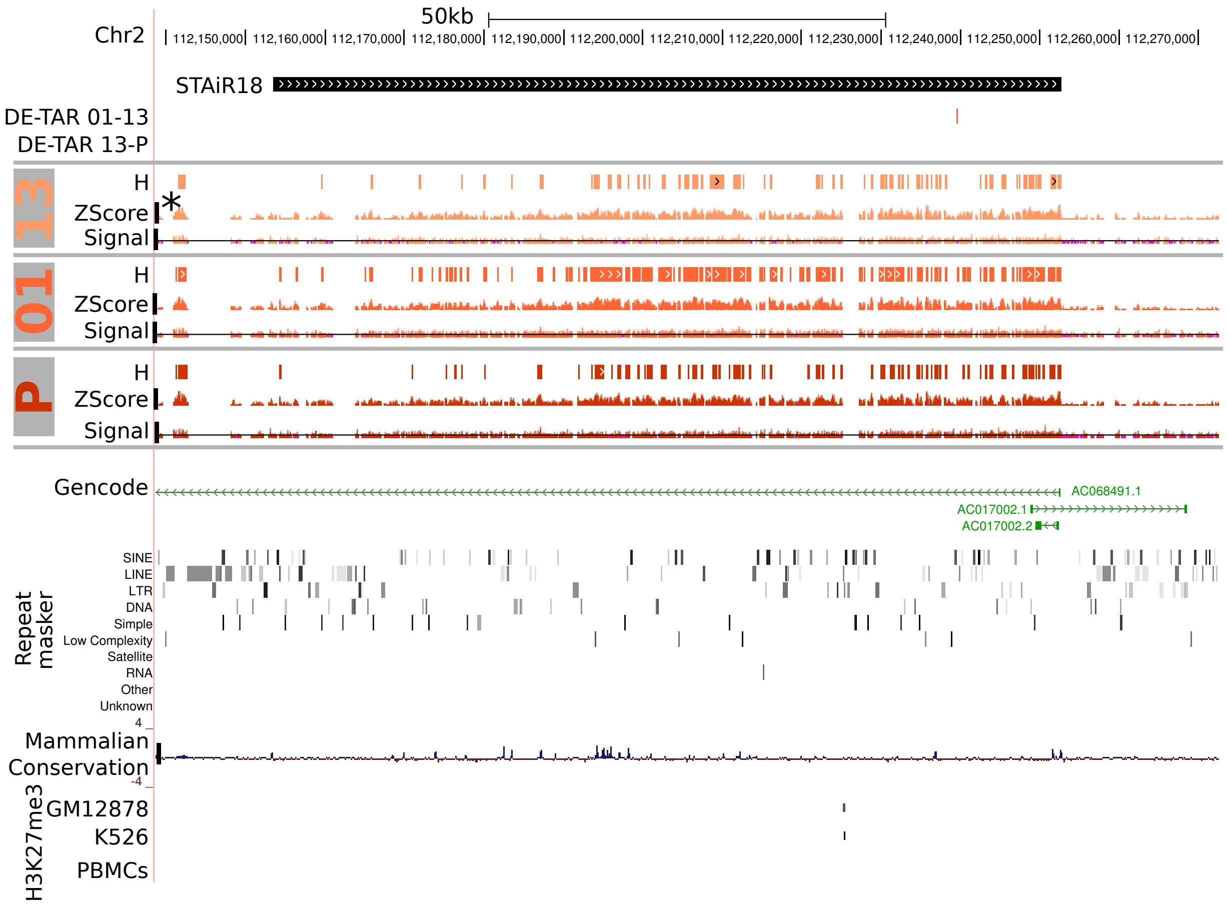

Figure S13: **STAT3-induced RNA 18 (STAiR18).** INA6 cells were cultured in absence of IL-6 for $13h$ (13), restimulated for $1h$ (01), or permanently grown in presence of IL-6 (P) and RNA expression was analyzed using tiling arrays. `TileShuffle` identified strong expression for all states but no significant differential expression at the stringent cutoff of q < 0.005. This putative macro RNA is located intergenic and overlaps the complex locus of an annotated non-coding RNA (AC068491.1, `Gencode` v12) of multiple isoforms. (*) Wiggle track scale bars indicate y-axis scales of (6,16), (0,10), (-3.5,3.5), and (-4,4) for signal, z-score, differential signal, and conservation, respectively.

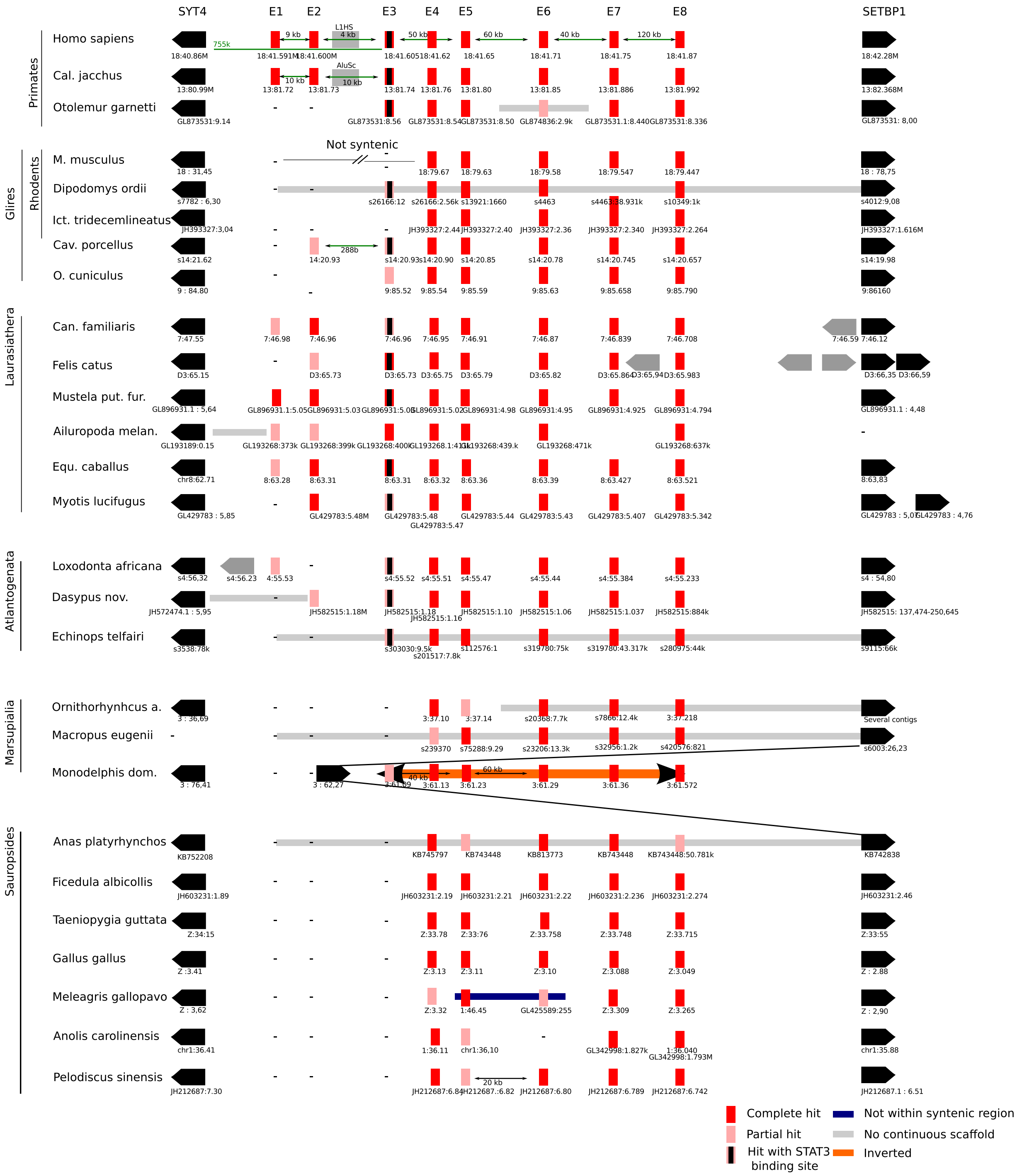

Figure S14: **STAT3-induced RNA 1 (STAiR1) conserved elements.** STAiR1 was aligned to all `Ensembl` provided vertebrate genomes using BLAST. Several conserved elements throughout STAiR1 that did not overlap annotated repeat elements were selected for further analysis.

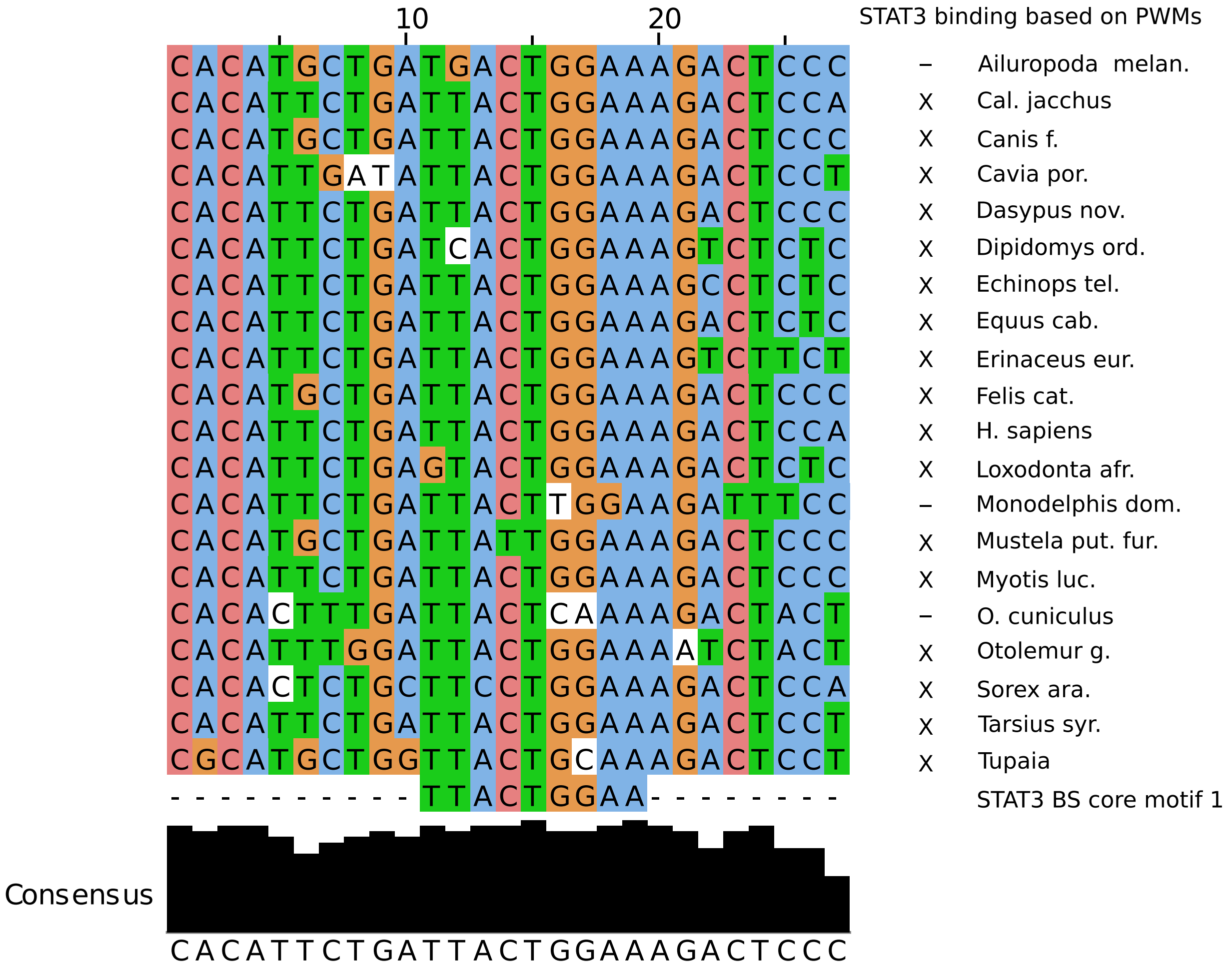

Figure S15: **Conserved STAT3 binding site in STAiR1.** Element E3 sequences were aligned using `clustalw` and trimmed to the occurrence of a STAT3 consensus motif. STAT3 binding was inferred using PWM data from [20, 21]

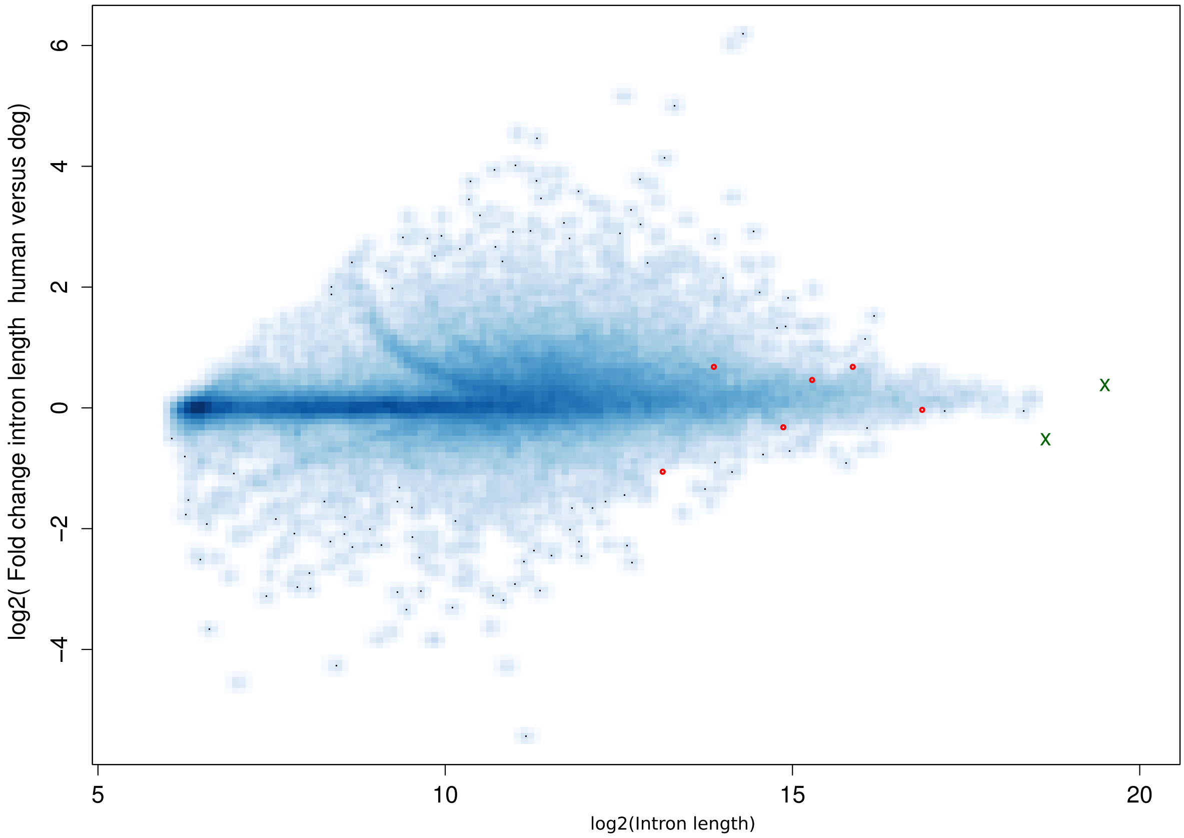

Figure S16: **Variation of intron lengths between man and dog over human intron length.** Lengths of introns conserved fully in man and dog were computed. The log2 fold changed of human versus canine intron length was plotted on the y, log2 of human intron length on the x axis. Changes in distances within STAiR1 conserved elements between man and dog, versus human distances were plotted in red circles. Distances of the terminal elements to the adjacent protein coding genes *SYT4* and *SETBP1* were plotted in green x.

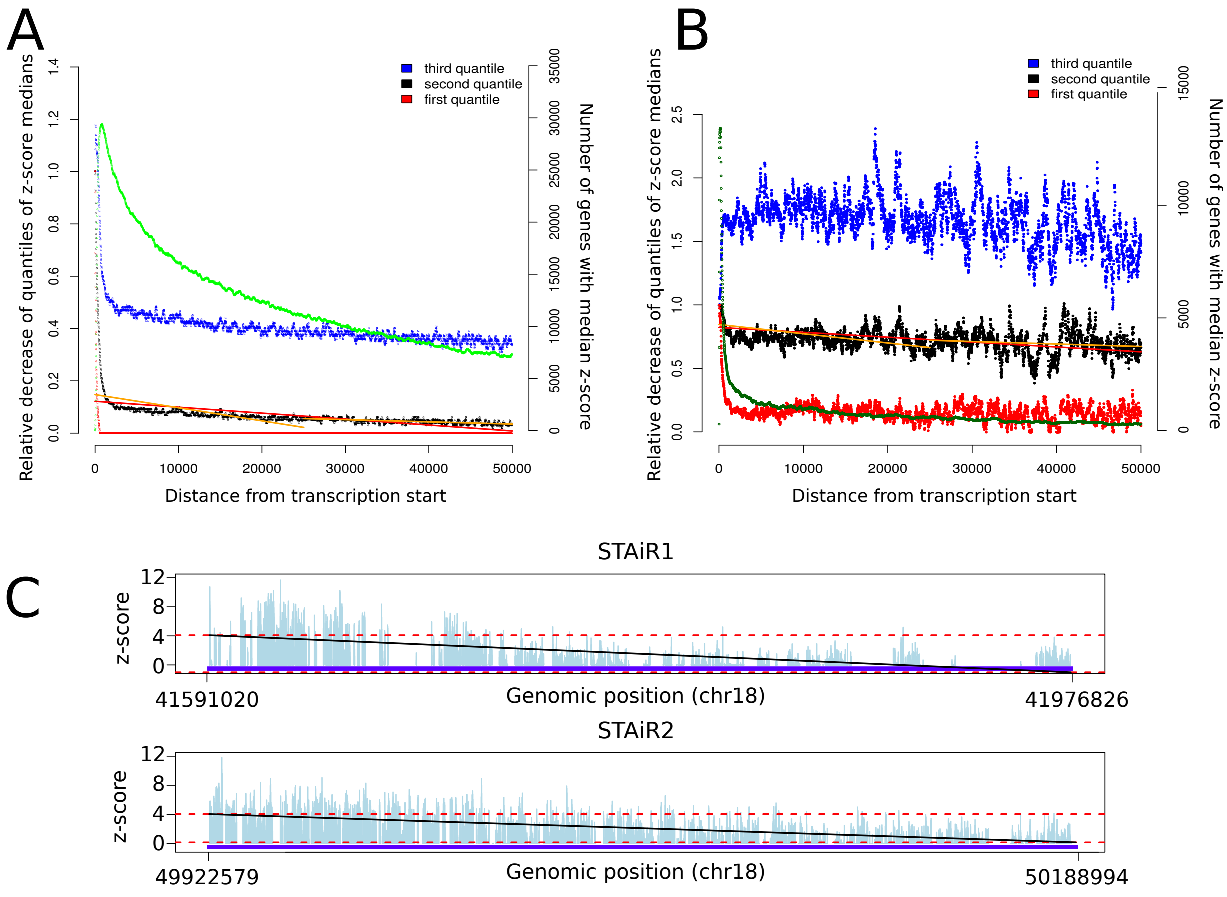

Figure S17: **Continuously transcribed genomic intervals are characterized by a decreasing tiling array expression signal.** (**A**) Continuous primary transcripts are characterized by a steady signal decay in tiling array data. All human protein coding genes which are expressed in the STAT3 data set after restimulation for $1h$ have been aligned by their annotated transcription start site (TSS). For each gene, tiling array signal z-scores have been scaled to 1.0 at the TSS. Distribution of z-scores over distance to start site are shown, integrating all genes which are expressed at all in the data set and are intronic at the respective distance to TSS. The green line gives the number of genes which have been included for the respective data point, red, black, and blue lines the first, second, and third quartile of the distribution, respectively. The median is characterized by a steep descent close to the TSS (-5.01 per MB) and a continuous decay of -0.84 per MB over the remaining range of TSS distance. The overall decay over the complete range of distances to TSS is -2.27 per MB (red straight line). A similar, but due to less data more rugged decay is observed for exonic data (**B**). (**C**) Identified DE macro ncRNAs show a similar signal descent as observed in A, which hints at a continuous transcription of these intervals. Also, the direction of transcription may be inferred for these macroRNAs in analogy to A.

Table S12: **DE-macroRNAs.** Table summarizing identified DE-macroRNAs. **ID:** identifier of DE-macroRNA; **Name:** internal name of DE-macroRNA (if any assigned); **Cov:** coverage of DE-macroRNA by DE-TAR intervals; **Sil:** denotes the `stairFinder` silhouette score and **Score** the overall `stairFinder` score; **Chr**, **Start** and **End:** the genomic location of the macroRNA, **Type:** the genomic category the macroRNA falls into (IG - intergenic, E - overlapping exons, EN - overlapping non-coding exons, I - located in introns, ES - joint start but different end as mRNA, P - presumed primary transcript); **Gene:** indicates the protein coding gene which contains or overlaps macroRNA; **Exp_CC**, **Exp_P53**, **Exp_STAT3:** tiling array survey the macroRNA was differentially expressed; **Comment:** any comment and information about known ncRNAs overlapping the macroRNA.

ID	Name	Cov	Sil	Score	Chr	Start	End	Type	Gene	Ex_CC	Ex_P53	Ex_STAT3	Comment
maR-1		18.5	92.5	22666	chr5	127419281	127544384	P	SLC12A2	0	1	0	
maR-2		14.5	96.5	22491	chr17	46626185	46724063	P	HOXB-AS3	0	1	0	
maR-3		10.5	94.9	20117	chr2	200134573	200326143	P	SATB2	0	1	0	
maR-4		11.4	93.7	15539	chr17	56429861	56494480	P	RNF43	0	1	0	
maR-5		5.7	70.6	12421	chr10	114710009	114927437	P	TCF7L2	0	1	0	
maR-6		5.2	71.5	11847	chr2	87755362	87814710	EN	LINC00152	1	1	1	lincRNA, longer in CC and STAT3 but not diff. there
maR-7		42.6	94.7	11829	chr15	63334884	63364088	P	TPM1	1	1	0	
maR-8		9.8	94.4	10345	chr17	46626185	46724063	EN		1	1	1	miR hostgene
maR-9		17.1	86.5	9415	chr14	21677448	21737989	P	HNRNPC	0	1	1	
maR-10		22.6	95.1	8940	chr5	138610886	138654113	E	MTR3	1	1	1	
maR-11		52.6	98.2	8537	chr16	9196774	9213757	E	C16ORF72	1	1	1	
maR-12		18.5	27.2	50276	chr15	82640603	83233508	E		0	1	0	
maR-13		24.1	97.3	32673	chr4	144261292	144395123	P	GAB1	0	1	1	
maR-14		19.5	95.9	31041	chr2	96553100	96605535	E	ANKRD36C	1	1	1	
maR-15		35.00	96.7	29071	chr2	173292604	173372186	P	ITGA6	0	1	0	
maR-16		8.9	84.8	27752	chr15	99251777	99508803	E	IGF1R	1	1	0	
maR-17		42.5	92.4	25912	chr7	116165376	116202367	P	CAV1	0	1	0	
maR-18		23.3	96.3	25335	chr6	54712575	54811493	P	FAM83B	0	1	0	
maR-19		15.3	92.9	24827	chr17	43582572	43663011	E		0	1	0	

ID	Name	Cov	Sil	Score	Chr	Start	End	Type	Gene	Ex_CC	Ex_P53	Ex_STAT3	Comment
maR-20		17.4	94.9	24646	chr4	87932770	88062243	E	AFF1	0	1	0	
maR-21		33.00	90.2	24273	chr4	106822308	106858303	E	NPNT	0	1	0	
maR-22		7.3	95.4	24009	chr10	52076229	52395961	P	SGMS1	0	1	0	
maR-23		6.3	93.7	23894	chr17	62891406	62908341	E	LRRC37A3	0	1	0	
maR-24		41.00	89.1	19725	chr14	60716572	60768042	P	PPM1A	0	1	0	
maR-25		41.1	98.00	18292	chr2	111881424	111925974	P	BCL2L11	0	1	1	
maR-26		22.5	93.1	18220	chr6	18389190	18469131	P	RNF144B	0	1	0	
maR-27		19.00	98.1	15772	chr7	20371792	20454781	P	ITGB8	0	1	0	
maR-28		55.1	99.9	15745	chr8	74209940	74230161	P	RDH10	0	1	0	
maR-29		17.00	87.4	13748	chr1	245318243	245399217	ES	KIF26B	0	1	0	
maR-30		9.2	94.6	13672	chr3	149532698	149642694	P	RNF13	0	1	0	
maR-31		6.4	98.8	13469	chr5	60629894	60841385	P	ZSWIM6	0	1	1	
maR-32		17.00	94.00	12827	chrX	88655983	88731544	EN	RP13-348B13.2	0	1	0	lincRNA
maR-33		9.8	98.4	12545	chrY	2879261	2976244	EN	linc00278	0	1	0	lincRNA
maR-34		7.7	53.00	11459	chr15	100174764	100212490	P	MEF2A	0	1	0	
maR-35		7.2	80.7	11273	chr15	85748543	85777210	E	RP11-561C5.3	0	1	0	
maR-36		5.00	96.2	10849	chr12	44246475	44478028	E	TMEM117	0	1	0	
maR-37		5.6	10.8	10560	chr4	99391694	99579364	P	TSPAN5	0	1	0	
maR-38		23.8	96.4	9554	chr17	45093034	45133094	EN	RP11-156P1.3	1	1	1	lincRNA
maR-39		15.9	99.1	9319	chr8	134249383	134308157	P	NDRG1	0	1	0	
maR-40		27.1	93.7	9210	chr2	172380452	172414397	P	CYBRD1	0	1	0	
maR-41		21.8	99.7	8165	chr4	170908379	170945902	P	MFAP3L	0	1	0	
maR-42		94.7	99.2	8080	chr16	85437862	85446703	IG		1	1	0	
maR-43	STAiR1	10.1	18.7	21501	chr18	41591020	41976826	IG		0	0	1	
maR-44	STAiR12	14.9	99.3	22445	chr12	104851810	105004739	ES	CHST11	0	0	1	
maR-45	STAiR2	13.3	99.7	10944	chr18	49922579	50188994	I	DCC	0	0	1	
maR-46		28.9	99.3	8145	chr11	111221493	111250722	P	POU2AF	0	0	1	

ID	Name	Cov	Sil	Score	Chr	Start	End	Type	Gene	Ex_CC	Ex_P53	Ex_STAT3	Comment
maR-47		11.8	98.3	22672	chr2	36590795	36789955	P	CRIM1	1	0	0	
maR-48		47.6	99.4	11386	chr5	98104819	98133830	P	RGMB	1	0	0	
maR-49		11.2	95.9	13360	chr15	32908000	32932636	P	ARHGAP11A	1	0	0	
maR-50		9.4	97.1	9266	chr15	30918495	30982898	E	ARHGAP11B	1	0	0	
maR-51		21.3	48.1	24408	chr9	21442869	21559668	EN	MIR31HG	1	0	0	miR hostgene
maR-52		34.2	98.1	23545	chr5	95222066	95286499	P	ELL2	1	0	0	
maR-53		12.5	87.2	14604	chr4	177595309	177712068	P	VEGFC	1	0	0	
maR-54		57.80	-87.20	150765	chr9	20346052	20638345	P	CELF2	0	1	1	
maR-55		31.10	-97.50	93298	chr15	81072954	81244747	P	KIA1199	1	1	0	
maR-56		15.90	-98.90	45247	chr2	153191751	153506348	P	FMNL2	0	1	0	
maR-57		8.80	-1.20	25454	chr4	82564046	82950759	E	RP11-689K5.3	0	1	0	snoRNA host gene
maR-58		5.00	-75.70	15985	chr1	174133990	174509554	P	RABGAP1L	0	1	0	
maR-59		6.00	-43.00	14111	chr11	19734881	20143144	P	NAV2	1	0	0	
maR-60		11.80	-69.00	28638	chr6	73339569	73610550	ES	KCNQ5	1	1	1	

Table S13: **DE-macroRNA overlap with known non-coding RNAs.** Overlaps in nucleotides between DE-macroRNAs and known non-coding RNAs. Annotation datasets are described in Supplemental Table S28. Column heading **Survey** indicates if overlap is for cell cycle (CC) or for P53 or STAT3 (IL-6) pathway, **Type** indicates the genomic category the macroRNA falls into (IG - intergenic, E - overlapping exons, EN - overlapping non-coding exons, I - located in introns, ES - joint start but different end as mRNA, P - presumed primary transcript). The length of the genomic region containing the macroRNA is given by **Length**.

macroRNA			short ncRNAs		long ncRNAs				CARs		Secondary Structures			
ID	Survey	Type	miRNAs	sno/scaRNAs	Gencode	lncRNAdb	lincRNAs	TUCP	intergenic	intron	RNAz	SISSIz	Evofold	Length
maR-6	P53	EN	0	0	0	0	0	0	1115	0	870	1399	18	59348
maR-8	P53	EN	180	0	379	0	1169	0	0	0	197	2640	1509	97878
maR-10	P53	E	0	200	0	690	0	0	0	91	1474	1026	175	43227
maR-11	P53	E	0	0	0	0	0	0	0	0	0	610	34	16983
maR-12	P53	E	0	0	0	0	0	0	0	0	194	1985	277	592905
maR-14	P53	E	0	0	0	0	0	0	0	0	0	490	0	52435
maR-16	P53	E	0	0	0	0	0	0	0	76619	2098	5989	121	257026
maR-19	P53	E	0	0	0	0	0	11	0	0	0	225	0	80439
maR-20	P53	E	0	0	0	0	0	0	0	0	909	2013	0	129473
maR-21	P53	E	0	0	0	0	0	0	0	0	168	308	0	35995
maR-23	P53	E	0	0	0	0	0	0	0	0	0	0	0	16935
maR-29	P53	ES	0	0	0	0	0	0	0	708	2119	2153	0	80974
maR-32	P53	EN	0	0	469	0	469	0	0	0	2358	570	0	75561
maR-33	P53	EN	0	0	469	0	469	0	0	0	0	329	0	96983
maR-35	P53	E	0	0	0	0	0	1261	0	0	0	102	0	28667
maR-36	P53	E	0	0	0	0	0	0	0	0	4330	2542	22	231553
maR-38	P53	EN	0	0	0	0	0	0	0	0	0	443	0	40060
maR-42	P53	IG	0	0	0	0	0	0	0	0	0	0	0	8841
maR-43	IL6	IG	0	0	0	0	2205	0	0	0	5699	3227	132	385806
maR-44	IL6	ES	0	0	0	0	0	0	0	0	2011	4817	0	152929

macroRNA			short ncRNAs		long ncRNAs				CARs		Secondary Structures			
ID	Survey	Type	miRNAs	sno/scaRNAs	Gencode	lncRNAdb	lincRNAs	TUCP	intergenic	intron	RNAz	SISSIz	Evofold	Length
maR-45	IL6	I	0	0	0	0	0	0	0	0	1742	3133	29	266415
maR-50	CC	E	0	0	0	0	0	0	0	0	0	355	0	64403
maR-51	CC	EN	0	0	0	0	0	0	0	59140	690	774	179	64433
maR-57	P53	E	0	0	0	0	0	416	0	0	4090	5849	18	386713
maR-60	CC	ES	0	0	0	0	0	0	0	0	4042	4259	21	270981

Table S14: **DE-macroRNA overlap with regulatory sites and epigenetically modified regions.** Overlaps in nucleotides between DE-macroRNAs and putative promoter regions, transcription factor bindings sites and epigenetically modified regions. Annotation datasets are described in Supplemental Table S28. Column heading **Survey** indicates if overlap is for cell cycle (CC) or for P53 or STAT3 (IL-6) pathway, **Type** indicates the genomic category the macroRNA falls into (IG - intergenic, E - overlapping exons, EN - overlapping non-coding exons, I - located in introns, ES - joint start but different end as mRNA, P - presumed primary transcript). The length of the genomic region containing the macroRNA is given by **Length**.

macroRNA										TFBs		CpG		
ID	Survey	Type	H3K27ac	H3K36me3	H3K4me1	H3K4me3	H3k27me3	POL-II	DNaseI	Encode	Transfac	CpG	and H3K4me3	Length
maR-6	P53	EN	58448	59348	59348	39098	59348	58923	7028	7669	1655	0	0	59348
maR-8	P53	EN	85678	97878	95678	91953	97878	53178	45858	43684	14904	13821	13325	97878
maR-10	P53	E	29390	43227	35065	26140	1615	35215	10630	10231	1567	1146	1146	43227
maR-11	P53	E	16983	16983	16983	12050	0	16983	4487	1696	935	0	0	16983
maR-12	P53	E	131200	401563	304042	91350	423880	100925	3980	3382	4139	10915	10049	592905
maR-14	P53	E	34500	52435	43223	4300	8962	2000	2000	1878	0	0	0	52435
maR-16	P53	E	129750	257026	220276	78775	257026	49525	59220	53708	9902	1139	890	257026
maR-19	P53	E	13319	80439	26919	14469	80439	21244	4670	4761	912	870	870	80439
maR-20	P53	E	92181	129473	112023	58081	22875	42206	34070	23605	2633	0	0	129473
maR-21	P53	E	3650	35995	10577	0	35995	575	2853	2486	552	0	0	35995
maR-23	P53	E	607	16935	6032	0	16935	325	210	0	171	0	0	16935
maR-29	P53	ES	675	11050	31274	7284	80974	2175	11787	9288	2260	2004	2004	80974
maR-32	P53	EN	0	40386	14475	0	75561	0	590	510	315	0	0	75561
maR-33	P53	EN	0	0	0	0	0	0	630	1027	0	0	0	96983
maR-35	P53	E	5989	27553	14014	4989	28667	8117	0	0	0	86	86	28667
maR-36	P53	E	11383	131808	60083	5850	88508	725	13700	11549	2499	0	0	231553
maR-38	P53	EN	1625	40060	6250	725	21975	5925	0	0	674	0	0	40060
maR-42	P53	IG	287	8841	0	0	279	0	0	0	0	0	0	8841
maR-43	IL6	IG	15625	16925	64850	6600	347156	2350	18080	13247	4910	0	0	385806
maR-44	IL6	ES	94360	152929	140454	76110	15910	26360	39959	31987	2763	585	585	152929

macroRNA										TFBs		CpG		
ID	Survey	Type	H3K27ac	H3K36me3	H3K4me1	H3K4me3	H3k27me3	POL-II	DNaseI	Encode	Transfac	CpG	and H3K4me3	Length
maR-45	IL6	I	273	1775	11048	275	181390	0	13480	7098	7881	0	0	266415
maR-50	CC	E	15863	64403	22663	8738	8828	20163	3170	1699	26	70	70	64403
maR-51	CC	EN	49680	64433	60283	26505	0	60103	13190	10991	2684	0	0	64433
maR-57	P53	E	14125	83850	107150	18025	386713	7500	20940	18115	6054	0	0	386713
maR-60	CC	ES	62260	270981	143110	26435	236806	5875	30380	16900	5422	0	0	270981

Table S15: **DE-macroRNA overlap with repeat regions.** Overlaps are calculated by using the Bioconductor `genomeIntervals` package [6]. The significance of the observed overlap is assessed by sampling 100 genomic loci for each macroRNA preserving macroRNA length. Odds ratios of observed versus expected relative overlaps are calculated and tested by Fisher's exact test for significant enrichment or depletion (see Materials and Methods). Column heading **Repeat class** indicates type of repeat as indicated in the UCSC `RepeatMasker` track (December 2013). Remaining columns indicate the results (**log2(Odds ratio)**, **P-value**, and 95% confidence interval) and the data (**DE-macroRNAs**: number of overlapping or non-overlapping nucleotides of DE-macroRNAs with repeats; **BG**: average number of overlapping or non-overlapping nucleotides of random intervals with repeats) of Fisher's exact test. Only significantly enriched or depleted overlaps of at least two fold are reported, i.e. P-value < 0.01 and $|\log2(\text{Odds ratio})| > 1$.

		Fisher's exact test			overlap		no overlap	
	Repeat class	log2(Odds ratio)	P-value	95% CI	DE-macroRNAs	BG	DE-macroRNAs	BG
maR-10	Alu	1.90	0.00e+00	[1.80, 1.95]	11511	1959	31716	19760
	DNA	-1.70	2.36e-100	[-1.82,-1.51]	557	863	42670	20856
	LINE	-2.40	0.00e+00	[-2.47,-2.32]	2260	4888	40967	16831
	RNAs	1.70	5.62e-07	[0.94, 2.52]	109	17	43118	21702
	Simple	-2.90	6.38e-42	[-3.36,-2.38]	47	170	43180	21549
	SINE	1.60	0.00e+00	[1.52, 1.65]	12417	2576	30810	19142
maR-11	LINE	-2.70	0.00e+00	[-2.81,-2.54]	633	1740	16350	7045
	Simple	-1.60	2.64e-08	[-2.19,-1.00]	42	65	16941	8721
maR-12	LINE	-1.30	0.00e+00	[-1.32,-1.28]	53612	56857	539293	232226
maR-14	Alu	-4.40	0.00e+00	[-4.58,-4.23]	306	2821	52129	22703
	LINE	-2.70	0.00e+00	[-2.73,-2.57]	2080	5262	50355	20263
	Low_compl	-2.60	1.08e-36	[-3.12,-2.18]	53	160	52382	25364
	Simple	-2.00	5.41e-27	[-2.36,-1.59]	89	169	52346	25356
	SINE	-4.80	0.00e+00	[-4.93,-4.58]	306	3486	52129	22038
maR-16	LINE	-1.90	0.00e+00	[-1.92,-1.86]	16837	22263	240189	85692
	LTR	-1.80	0.00e+00	[-1.88,-1.79]	6827	9580	250199	98375
	RNAs	2.00	3.14e-28	[1.58, 2.46]	467	49	256559	107906
	Simple	-1.30	9.61e-92	[-1.39,-1.15]	1106	1112	255920	106843
maR-19	Alu	1.40	0.00e+00	[1.39, 1.50]	18178	4022	62261	37426
	LTR	-3.00	0.00e+00	[-3.11,-2.90]	944	3599	79495	37849
	RNAs	2.20	2.50e-27	[1.71, 2.71]	344	39	80095	41410
	SINE	1.30	0.00e+00	[1.24, 1.33]	20656	5150	59783	36298
maR-20	LINE	-1.90	0.00e+00	[-1.90,-1.82]	8583	14909	120890	57934
	LTR	-2.60	0.00e+00	[-2.72,-2.56]	1904	6201	127569	66641
maR-21	DNA	-1.40	5.93e-47	[-1.55,-1.17]	433	530	35562	16932
	LINE	-1.00	9.07e-192	[-1.12,-0.98]	4347	3864	31648	13598
	LTR	-4.20	0.00e+00	[-4.37,-3.94]	205	1617	35790	15845
maR-23	Alu	1.40	2.23e-172	[1.33, 1.55]	4637	993	12298	7129
	LINE	-2.70	0.00e+00	[-2.86,-2.58]	629	1646	16306	6476
	Low_compl	-1.80	1.43e-08	[-2.45,-1.13]	33	54	16902	8067

	Repeat class	log2(Odds ratio)	P-value	95% CI	overlap		no overlap	
				,	DE-macroRNAs	BG	DE-macroRNAs	BG
	SINE	1.20	1.48e-138	[1.12, 1.33]	4872	1196	12063	6926
maR-29	LTR	-2.50	0.00e+00	[-2.64,-2.44]	1191	3001	79783	34562
maR-32	LINE	1.60	0.00e+00	[1.55, 1.63]	32922	8528	42639	33324
	Simple	1.60	1.59e-84	[1.38, 1.73]	1659	318	73902	41534
	SINE	-1.30	0.00e+00	[-1.37,-1.24]	3611	4608	71950	37243
maR-33	LINE	1.70	0.00e+00	[1.66, 1.74]	43780	9012	53203	35569
maR-35	LINE	-1.10	1.12e-142	[-1.17,-1.00]	2640	2699	26027	12522
	LTR	2.70	0.00e+00	[2.64, 2.85]	9069	983	19598	14238
	RNAs	3.30	1.57e-24	[2.40, 4.30]	199	11	28468	15210
maR-38	LTR	-4.60	0.00e+00	[-4.84,-4.33]	144	1611	39916	18690
	RNAs	2.70	2.91e-30	[2.13, 3.42]	302	23	39758	20278
maR-42	LTR	-2.40	1.27e-76	[-2.73,-2.17]	159	371	8682	3708
maR-43	Alu	-1.10	0.00e+00	[-1.13,-1.07]	16638	17423	369168	180722
STAiR1	LINE	1.30	0.00e+00	[1.31, 1.35]	149121	39750	236685	158395
	RNAs	1.20	4.60e-19	[0.94, 1.55]	515	112	385291	198034
maR-44	LINE	-1.20	0.00e+00	[-1.26,-1.19]	14611	16557	138318	66840
STAiR12								
maR-45	Alu	-1.10	0.00e+00	[-1.11,-1.04]	12213	12361	254202	122063
STAiR2	LTR	-1.30	0.00e+00	[-1.31,-1.23]	11216	12886	255199	121538
maR-50	LTR	-1.30	1.40e-171	[-1.41,-1.23]	1981	2046	62422	25809
	RNAs	1.80	9.13e-11	[1.19, 2.54]	181	22	64222	27833
maR-51	LINE	-1.60	0.00e+00	[-1.66,-1.54]	5016	6388	59417	25005
maR-57	Alu	-1.00	0.00e+00	[-1.05,-0.99]	20942	19023	365771	163523
maR-60	LTR	-1.20	0.00e+00	[-1.27,-1.19]	11067	11732	259914	117152
	RNAs	3.00	3.98e-133	[2.65, 3.32]	1337	81	269644	128804
maR-6	DNA	-1.00	1.20e-58	[-1.13,-0.88]	1154	1107	58194	27781
	LINE	-2.30	0.00e+00	[-2.41,-2.27]	2711	5623	56637	23264
	LTR	-1.10	2.25e-158	[-1.19,-1.03]	2665	2661	56683	26227
maR-8	Alu	-3.60	0.00e+00	[-3.74,-3.52]	858	5090	97020	46471
	DNA	-4.40	0.00e+00	[-4.61,-4.12]	150	1574	97728	49986
	LINE	-7.30	0.00e+00	[-7.55,-7.07]	154	10244	97724	41316
	Low_compl	1.30	7.54e-51	[1.13, 1.51]	1299	277	96579	51283
	SINE	-3.30	0.00e+00	[-3.35,-3.18]	1494	6679	96384	44881

5.1 Evolutionary selection acting on STAiR1 compared to its neighbor protein-coding genes

STAiR1 is located in intergenic space of the protein-coding genes of SETBP1 (SET binding protein 1) and SYT4 (Synaptotagmin IV). The gene *SETBP1* encodes a protein that binds to the protein product of *SET* (SET nuclear oncogene). High expression of both SETBP1 or SET is associated with myeloid malignancies [22, 23]. We found STAiR1 to be differentially expressed in a human myeloma cell line (INA-6) depending on STAT3 expression, and asked ourselves if STAiR1 expression may interfere with *SETBP1* expression in *cis*. If STAiR1 would function in *cis*, it should evolve closely with its protein-coding target gene *SETBP1*, i.e. substitution rates should not differ largely. Wong and Nielsen, *Genetics* 2004 [24], introduced a phylogenetic model to detect faster evolution in non-coding regions when compared to a protein-coding "reference" gene. Selection on protein-coding genes is usually assessed by the d_N/d_S ratio of non-synonymous substitution rates (d_N) and synonymous substitution rates (d_S). Accordingly, Wong and Nielsen define $\zeta = d_{NC}/d_S$, with d_{NC} denoting the nucleotide substitution rate in the non-coding region under the HKY85 model [25] for neutral evolution. On the basis of a phylogenetic tree (which is assumed to be the same for the coding and non-coding sequences) they propose to compare the likelihoods of this tree under different ranges of ζ and to conduct likelihood ratio tests to detect which model is more likely. They propose three models, a model for neutral evolution (constraints: $0 < \zeta_0 < 1$ and $\zeta_1 = 1$), and two different models for faster evolution (two-category model: $0 < \zeta_0 < 1$ and $\zeta_1 \geq 1$, three-category model $0 < \zeta_0 < 1$, $\zeta_1 = 1$, and $\zeta_2 > 1$).

We constructed a phylogenetic tree from orthologous sequences (*Homo sapiens* - hg19, *Callithrix jacchus* - calJac3.2.1, *Cavia porcellus* - cavPor3, *Canis familiaris* - canFam3.1, *Felis catus* - felCat6.2, *Equus caballus* - equCab2, *Gallus gallus* - galGal4, *Anolis carolinensis* - anoCar2.0) of the conserved element 2 of STAiR1 and the `Multiz` alignment of a conserved region of the first exon in *SETBP1*. We ensured that the open reading frame for all orthologous sequences of the protein-coding was in line with the known ORF of the human *SETBP1* by visual inspection in the UCSC genome browser.

The likelihood of the phylogenetic tree for the three different models were calculated using `EvoNC` [24]. The estimated parameters for ζ and according likelihood ratio tests did not favor the two-, or the three-category model over the neutral model (Supplemental Table S16). Hence, no evidence is provided that STAiR1 evolves faster than *SETBP1*, thus the possibility of a *cis* regulatory function of STAiR1 cannot be excluded. The same analysis was conducted with an alignment containing STAiR1 and a conserved region of the second exon of *SYT4*, again *cis* regulation of STAiR1 cannot be excluded.

Model	Log likelihood	Selection on neighbor gene		Selection on STAiR1						LR-Test	
	of combined tree	κ	d_N/d_S	ζ_0		ζ_1		ζ_2		LR	P-value
				SETBP1							
Neutral	-3303.759	4.1035	0.0145	0.001	[0.37]	1.000	[0.62]				
Two-category	-3302.404	3.9299	0.0201	0.001	[0.37]	1.382	[0.62]			0.257	0.099
Three-category	-3301.316	4.2299	0.0144	0.001	[0.37]	1.000	[0.61]	11.061	[0.01]	0.086	0.086
				SYT4							
Neutral	-2951.751	5.1380	0.0269	0.001	[0.38]	1.000	[0.61]				
Two-category	-2951.752	5.1370	0.0269	0.001	[0.38]	1.001	[0.61]			1.001	1.000
Three-category	-2950.092	5.2851	0.0266	0.001	[0.38]	1.000	[0.61]	15.856	[0.006]	0.190	0.190

Table S16: **Evolutionary selection acting on STAiR1 compared to its neighbor protein-coding genes *SETBP1* and *SYT4*.** Log-likelihoods of phylogenetic trees under three different models as computed by `EvoNC` [24]. In detail, the parameters for a model reflecting neutral evolution (constraints: $0 < \zeta_0 < 1$ and $\zeta_1 = 1$), and two models reflecting faster evolution of STAiR1 compared to the according protein-coding gene (two-category model: $0 < \zeta_0 < 1$ and $\zeta_1 \geq 1$; three-category model $0 < \zeta_0 < 1$, $\zeta_1 = 1$, and $\zeta_2 > 1$) were calculated. Phylogenetic trees were derived from combined multiple alignments containing the conserved element 2 of STAiR1 (731bp) and either the `Multiz` alignment of a conserved region (192bp) of the first exon in *SETBP1* or a conserved region (180bp) of the second exon of *SYT4*. Numbers in brackets denote the probability to observe a particular value of ζ_0, ζ_1, or ζ_2. Likelihood ratio tests (**LR-Test**) have been computed for the neutral model compared to the two-category model with one degree of freedom ($df = 1$) and for the neutral model compared to the three-category model with two degrees of freedom ($df = 2$). **P-value** denotes the probability to receive a likelihood ratio (**LR**) at least as extreme as the observed one under the null hypothesis of neutral evolution. P-values were approximated by $-2 \log \mathrm{LR} \sim \chi^2_{df}$.

6 Disease associated ncRNAs

6.1 Clinical data of astrocytoma tumor subtypes

ID	Pathology	Age	Sex	Tumor location	OS (months)	Ki67
I-1	AI	9	F	cerebellar hemisphere	Alive after 104	5%
I-2	AI	10	M	parietal	Alive after 93	10%
I-3	AI	14	F	cerebellar hemisphere	Alive after 73	5%
I-4	AI	10	M	cerebellar hemisphere	Alive after 45	1%
III-1	AIII	32	F	temporal	107	5-10%
III-2	AIII	22	M	frontal	51	25%
III-3	AIII	45	M	frontal	Alive after 71	5-10%
III-4	AIII	23	M	parietal	Alive after 63	5%
IV-1	GBM	62	M	parietal	18	50%
IV-2	GBM	62	M	frontal	3	25%
IV-3	GBM	42	M	frontal	11	40%
IV-4	GBM	69	M	parietal	11	30-40%

Table S17: Clinical, pathological, and immunohistochemical data of presented tumors. For the proliferative marker Ki67, percentage values were attributed to each case evaluating ten fields (x400 magnification).

6.2 Content of the custom microarray - nONCOchip

Annotation	Number of probes	Fraction of probes
CDS (sense)	12254	0.062
CDS (antisense)	6034	0.030
5'UTRs (sense)	1553	0.007
5'UTRs (antisense)	1278	0.006
3'UTRs (sense)	23639	0.120
3'UTRs (antisense)	11183	0.057
Introns (sense)	39405	0.200
Introns (antisense)	40862	0.208
Pseudogenes	2761	0.014
Intergenic	59050	0.301
`Evofold`	16057	0.081
`RNAz`	1197	0.006
`SISSIz`	5338	0.027
CARs (intergenic)	319	0.001
CARs (intronic)	2004	0.010
lncRNAs (`Gencode`)	772	0.003
`lncRNAdb`	439	0.002
lincRNAs	1347	0.006
TUCP	723	0.003
TINs	2251	0.011
PINs	1595	0.008
miRNAs	248	0.001
snoRNAs/scaRNAs	354	0.001
DE-TARs (*bona fide* non-coding)		
CC (intergenic)	445	0.002
CC (intron)	2504	0.012
P53 (intergenic)	641	0.003
P53 (intron)	6598	0.033
STAT3 (intergenic)	266	0.001
STAT3 (intron)	405	0.002

Table S18: **nONCOchip custom microarray.** Absolute and relative number of probes on the nONCOchip overlapping with different annotation categories. Each probe overlapping to at least 95% (i.e. 57 nucleotides) with an annotation is counted. Detailed description of annotation categories is provided in Supplemental Table S28.

6.3 Differential expression of astrocytoma of grade I versus aggressive states (grade III or IV)

	Grade I < Aggressive				Grade I > Aggressive			
	Probes	Gencode v12 genes		Novel	Probes	Gencode v12 genes		Novel
		Probes	Genes			Probes	Genes	
All probes	7727	1764	1532	1747	5581	4132	2572	132
Bona fide non-coding probes	4860	105	93 (29)	1635	690	71	33 (6)	90
Protein-coding probes	1365	1365	1151	-	3881	3881	2382	-

Table S19: **Differential expression between astrocytoma of grade I versus aggressive states.** NONCOchip probes and their corresponding transcripts (Gencode v12) that are significantly differentially expressed between more benign versus aggressive states of diffuse astrocytoma (FDR < 0.05). Column headings **Probes**, **Gencode v12 genes**, **Novel** indicate unique number of significantly differentially expressed probes, unique number of these found in exons of known Gencode v12 genes, and unique number of novel probes, respectively. A probe is novel, if it does not overlap with any genetype known in Gencode v12. For Gencode v12 genes the number of significantly differentially expressed probes as well as the unique number of genes these probes map to are provided. Numbers in brackets indicate unique number of *bona fide* non-protein coding Gencode v12 genes.

6.4 *Bona fide* non-coding probes overlap with genomic annotation and DE-TARs

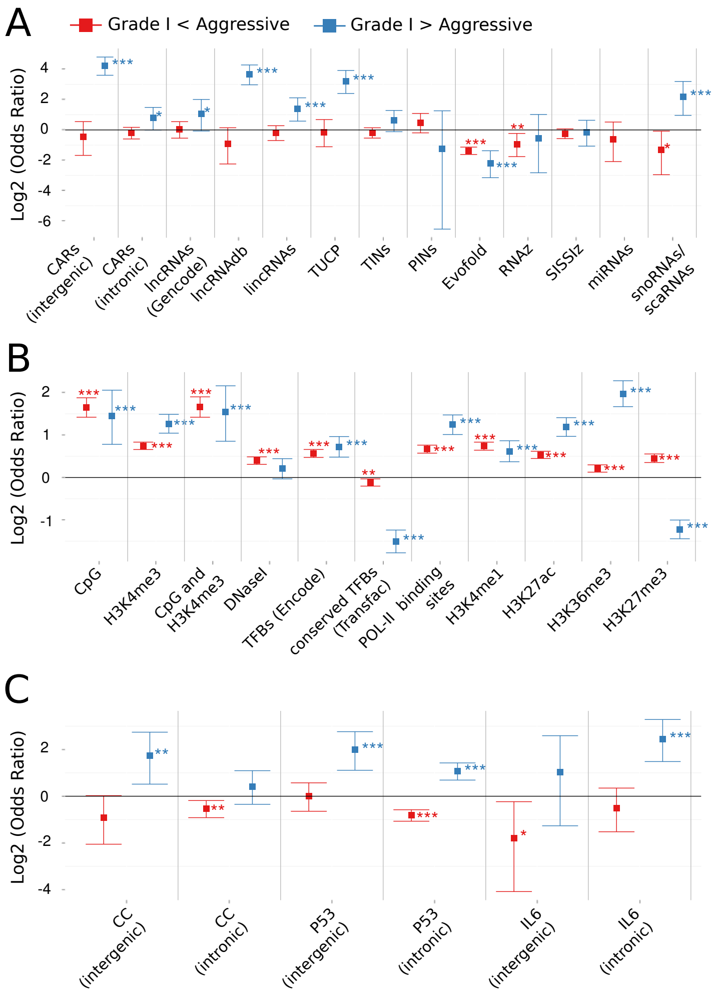

Figure S18: **DE-Probes overlap with different annotation categories.** Overlap of *bona fide* non-coding probes significantly differentially expressed between astrocytoma of grade I versus aggressive states of grade III and IV (FDR < 0.05) with different annotation categories. Grade I $>$ Aggressive, if expression of probe is higher in grade I than in aggressive states of astrocytoma, and Grade I $<$ Aggressive vice versa. Log2 transformed odds ratios and their 95% confidence interval for the respective annotation dataset are shown (annotations are described in detail in Supplemental Table S28). To assess the significance of the observed overlap, odds ratios of observed versus background (all probes on nONCOchip) relative overlaps are calculated and tested by Fisher's exact test for significant enrichment or depletion. *** indicates a p-value $p < 0.001$ of the observed versus random nucleotide overlaps, ** a p-value $p < 0.01$, and * a p-value $p < 0.05$, respectively. A probe is counted if it maps to at least 90% to an interval in the according annotation. (**A**) Overlap of *bona fide* non-coding probes with several classes of experimentally verified and predicted ncRNAs. (**B**) Overlap of *bona fide* non-coding probes with putative promoter regions, transcription factor binding sites, polII binding sites and epigenetically modified regions. (**C**) Overlap of *bona fide* non-coding probes with *bona fide* non-coding DE-TARs detected in at least one contrast in cell cycle, P53 or STAT3 experiment. For detailed output of Fisher's exact tests refer to Supplemental Tables S20, S21, and S22.

Table S20: ***Bona fide* non-coding DE-Probes overlap with known ncRNAs.** *Bona fide* non-coding DE-Probes overlapping with known ncRNAs. Annotation datasets are described in Supplemental Table S28. Overlaps are calculated by using the Bioconductor `genomeIntervals` package [6]. The significance of the observed overlap is assessed by calculating odds ratios of observed (DE-Probes) versus expected (all probes on microarray) relative overlaps. Odds ratios are calculated and tested by Fisher's exact test for significant enrichment or depletion (see Materials and Methods). Column heading **Annotation** indicates annotation datasets for which overlap is computed, and **Survey** if overlap is for probes that are higher expressed in astrocytoma of grade I than in aggressive states of grade III and IV (Grade I $>$ Aggressive) or if overlap is for probes that are lower expressed (Grade I $<$ Aggressive). Remaining columns indicate the results (**Odds ratio**, **P-value**, and 95% confidence interval for odds ratio - **95%CI**) and the data (**DE-Probes**: number of differentially expressed probes - FDR < 0.05 - completely overlapping with annotation, i.e. fraction of overlapping probe nucleotides $= 1$, or non-overlapping with annotation, i.e. fraction of overlapping probe nucleotides < 1; **BG**: number of array probes completely overlapping with annotation, i.e. fraction of overlapping probe nucleotides $= 1$, or non-overlapping with annotation, i.e. fraction of overlapping probe nucleotides < 1) of Fisher's exact test.

		Fisher's exact test			overlap		no overlap	
Annotation	Survey	Odds ratio	P-value	95% CI	DE-Probes	BG	DE-Probes	BG
CARs (intergenic)	Grade I > Aggressive	18.77	3.45e-25	[12.16,27.97]	28	279	662	123803
	Grade I < Aggressive	0.73	5.32e-01	[0.31, 1.46]	8	279	4852	123803
CARs (intron)	Grade I > Aggressive	1.73	3.52e-02	[1.00, 2.80]	17	1786	673	122296
	Grade I < Aggressive	0.87	3.24e-01	[0.66, 1.13]	61	1786	4799	122296
Evofold	Grade I > Aggressive	0.22	2.74e-11	[0.11, 0.39]	12	9259	678	114823
	Grade I < Aggressive	0.39	9.34e-39	[0.33, 0.46]	147	9259	4713	114823
lincRNAs	Grade I > Aggressive	2.64	6.48e-04	[1.50, 4.34]	16	1106	674	122976
	Grade I < Aggressive	0.88	4.82e-01	[0.62, 1.21]	38	1106	4822	122976
lncRNAdb	Grade I > Aggressive	12.66	1.74e-17	[7.86,19.49]	23	337	667	123745
	Grade I < Aggressive	0.53	1.16e-01	[0.21, 1.10]	7	337	4853	123745
lncRNAs (Gencode)	Grade I > Aggressive	2.10	4.53e-02	[0.95, 4.03]	9	777	681	123305
	Grade I < Aggressive	1.02	9.26e-01	[0.69, 1.46]	31	777	4829	123305
PINs	Grade I > Aggressive	0.43	7.35e-01	[0.01, 2.40]	1	421	689	123661
	Grade I < Aggressive	1.40	1.32e-01	[0.88, 2.13]	23	421	4837	123661
RNAz	Grade I > Aggressive	0.69	8.06e-01	[0.14, 2.03]	3	783	687	123299
	Grade I < Aggressive	0.52	6.59e-03	[0.30, 0.85]	16	783	4844	123299
miRNAs	Grade I > Aggressive	0.00	6.45e-01	[0.00, 2.84]	0	236	690	123846
	Grade I < Aggressive	0.65	3.96e-01	[0.24, 1.43]	6	236	4854	123846
snoRNAs or scaRNAs	Grade I > Aggressive	4.57	5.25e-04	[1.95, 9.16]	8	318	682	123764
	Grade I < Aggressive	0.40	3.84e-02	[0.13, 0.95]	5	318	4855	123764
SISSIz	Grade I > Aggressive	0.90	8.93e-01	[0.48, 1.55]	13	2590	677	121492
	Grade I < Aggressive	0.85	1.37e-01	[0.67, 1.05]	86	2590	4774	121492
TINs	Grade I > Aggressive	1.54	8.25e-02	[0.92, 2.43]	19	2236	671	121846
	Grade I < Aggressive	0.88	2.95e-01	[0.69, 1.10]	77	2236	4783	121846
TUCP	Grade I > Aggressive	9.25	2.67e-11	[5.29,15.15]	17	338	673	123744
	Grade I < Aggressive	0.91	8.88e-01	[0.46, 1.61]	12	338	4848	123744

Table S21: ***Bona fide* non-coding DE-Probes overlap with regulatory sites and epigenetically modified regions.** Number of *bona fide* non-coding DE-Probes overlapping with putative promoter regions, transcription factor bindings sites and epigenetically modified regions. Annotation datasets are described in Supplemental Table S28. Overlaps are calculated by using the Bioconductor `genomeIntervals` package [6]. The significance of the observed overlap is assessed by calculating odds ratios of observed (DE-Probes) versus expected (all probes on microarray) relative overlaps. Odds ratios are calculated and tested by Fisher's exact test for significant enrichment or depletion (see Materials and Methods). Column heading **Annotation** indicates annotation datasets for which overlap is computed, and **Survey** if overlap is for probes that are higher expressed in astrocytoma of grade I than in aggressive states of grade III and IV (Grade I > Aggressive) or if overlap is for probes that are lower expressed (Grade I < Aggressive). Remaining columns indicate the results (**Odds ratio**, **P-value**, and 95% confidence interval for odds ratio - **95%CI**) and the data (**DE-Probes**: number of differentially expressed probes - FDR < 0.05 - completely overlapping with annotation, i.e. fraction of overlapping probe nucleotides = 1, or non-overlapping with annotation, i.e. fraction of overlapping probe nucleotides < 1; **BG**: number of array probes completely overlapping with annotation, i.e. fraction of overlapping probe nucleotides = 1, or non-overlapping with annotation, i.e. fraction of overlapping probe nucleotides < 1) of Fisher's exact test.

		Fisher's exact test			overlap		no overlap	
Annotation	Survey	Odds ratio	P-value	95% CI	DE-Probes	BG	DE-Probes	BG
CpG	Grade I > Aggressive	2.74	3.07e-05	[1.72,4.16]	23	1543	667	122539
	Grade I < Aggressive	3.14	1.75e-36	[2.68,3.67]	185	1543	4675	122539
CpG and H3K4me3	Grade I > Aggressive	2.91	1.84e-05	[1.81,4.47]	22	1387	668	122695
	Grade I < Aggressive	3.17	1.03e-33	[2.68,3.73]	168	1387	4692	122695
DNaseI	Grade I > Aggressive	1.15	8.91e-02	[0.98,1.36]	214	34779	476	89303
	Grade I < Aggressive	1.32	1.62e-18	[1.24,1.40]	1648	34779	3212	89303
H3K27ac	Grade I > Aggressive	2.28	1.09e-26	[1.96,2.66]	358	39843	332	84239
	Grade I < Aggressive	1.45	2.14e-34	[1.36,1.54]	1975	39843	2885	84239
H3k27me3	Grade I > Aggressive	0.43	2.52e-27	[0.37,0.50]	364	89668	326	34414
	Grade I < Aggressive	1.37	6.64e-20	[1.28,1.47]	3796	89668	1064	34414
H3K36me3	Grade I > Aggressive	3.91	4.32e-50	[3.18,4.85]	582	71883	108	52199
	Grade I < Aggressive	1.16	9.31e-07	[1.09,1.23]	2987	71883	1873	52199
H3K4me1	Grade I > Aggressive	1.53	3.61e-07	[1.29,1.82]	501	78626	189	45456
	Grade I < Aggressive	1.67	7.80e-57	[1.56,1.78]	3609	78626	1251	45456
H3K4me3	Grade I > Aggressive	2.41	1.37e-28	[2.06,2.80]	309	31281	381	92801
	Grade I < Aggressive	1.68	3.46e-61	[1.58,1.78]	1756	31281	3104	92801
POL-II	Grade I > Aggressive	2.37	1.48e-24	[2.02,2.78]	245	23396	445	100686
	Grade I < Aggressive	1.59	1.10e-41	[1.49,1.70]	1311	23396	3549	100686
TFBs (Transfac)	Grade I > Aggressive	0.35	3.70e-34	[0.29,0.42]	149	54379	541	69703
	Grade I < Aggressive	0.92	6.11e-03	[0.87,0.98]	2033	54379	2827	69703
TFBs (Encode)	Grade I > Aggressive	1.65	7.35e-09	[1.40,1.95]	203	24995	487	99087
	Grade I < Aggressive	1.48	5.74e-31	[1.39,1.58]	1322	24995	3538	99087

Table S22: ***Bona fide* non-coding DE-Probes overlap with DE-TARs.** Number of *bona fide* non-coding DE-Probes overlapping with DE-TARs. Overlaps are calculated by using the Bioconductor `genomeIntervals` package [6]. The significance of the observed overlap is assessed by calculating odds ratios of observed (DE-Probes) versus expected (all probes on microarray) relative overlaps. Odds ratios are calculated and tested by Fisher's exact test for significant enrichment or depletion (see Materials and Methods). Column heading **Annotation** indicates annotation datasets for which overlap is computed, and **Survey** if overlap is for probes that are higher expressed in astrocytoma of grade I than in aggressive states of grade III and IV (Grade I $>$ Aggressive) or if overlap is for probes that are lower expressed (Grade I $<$ Aggressive). Remaining columns indicate the results (**Odds ratio**, **P-value**, and 95% confidence interval for odds ratio - **95%CI**) and the data (**DE-Probes**: number of differentially expressed probes - FDR < 0.05 - completely overlapping with annotation, i.e. fraction of overlapping probe nucleotides $= 1$, or non-overlapping with annotation, i.e. fraction of overlapping probe nucleotides < 1; **BG**: number of array probes completely overlapping with annotation, i.e. fraction of overlapping probe nucleotides $= 1$, or non-overlapping with annotation, i.e. fraction of overlapping probe nucleotides < 1) of Fisher's exact test.

		Fisher's exact test			overlap		no overlap	
Annotation	Survey	Odds ratio	P-value	95% CI	DE-Probes	BG	DE-Probes	BG
CC (intergenic)	Grade I > Aggressive	3.35	3.49e-03	[1.43,6.70]	8	433	682	123649
	Grade I < Aggressive	0.53	5.92e-02	[0.24,1.02]	9	433	4851	123649
CC (intron)	Grade I > Aggressive	1.34	2.15e-01	[0.79,2.13]	18	2437	672	121645
	Grade I < Aggressive	0.69	2.07e-03	[0.53,0.88]	66	2437	4794	121645
P53 (intergenic)	Grade I > Aggressive	3.99	2.27e-05	[2.16,6.80]	14	640	676	123442
	Grade I < Aggressive	1.00	1.00e+00	[0.64,1.49]	25	640	4835	123442
P53 (intron)	Grade I > Aggressive	2.10	6.91e-08	[1.61,2.69]	71	6436	619	117646
	Grade I < Aggressive	0.57	3.69e-13	[0.48,0.67]	146	6436	4714	117646
IL6 (intergenic)	Grade I > Aggressive	2.03	1.88e-01	[0.42,6.03]	3	266	687	123816
	Grade I < Aggressive	0.29	1.54e-02	[0.06,0.85]	3	266	4857	123816
IL6 (intron)	Grade I > Aggressive	5.49	4.16e-06	[2.80,9.75]	12	399	678	123683
	Grade I < Aggressive	0.70	2.98e-01	[0.35,1.27]	11	399	4849	123683

6.5 Proximal ncRNA – mRNA pairs

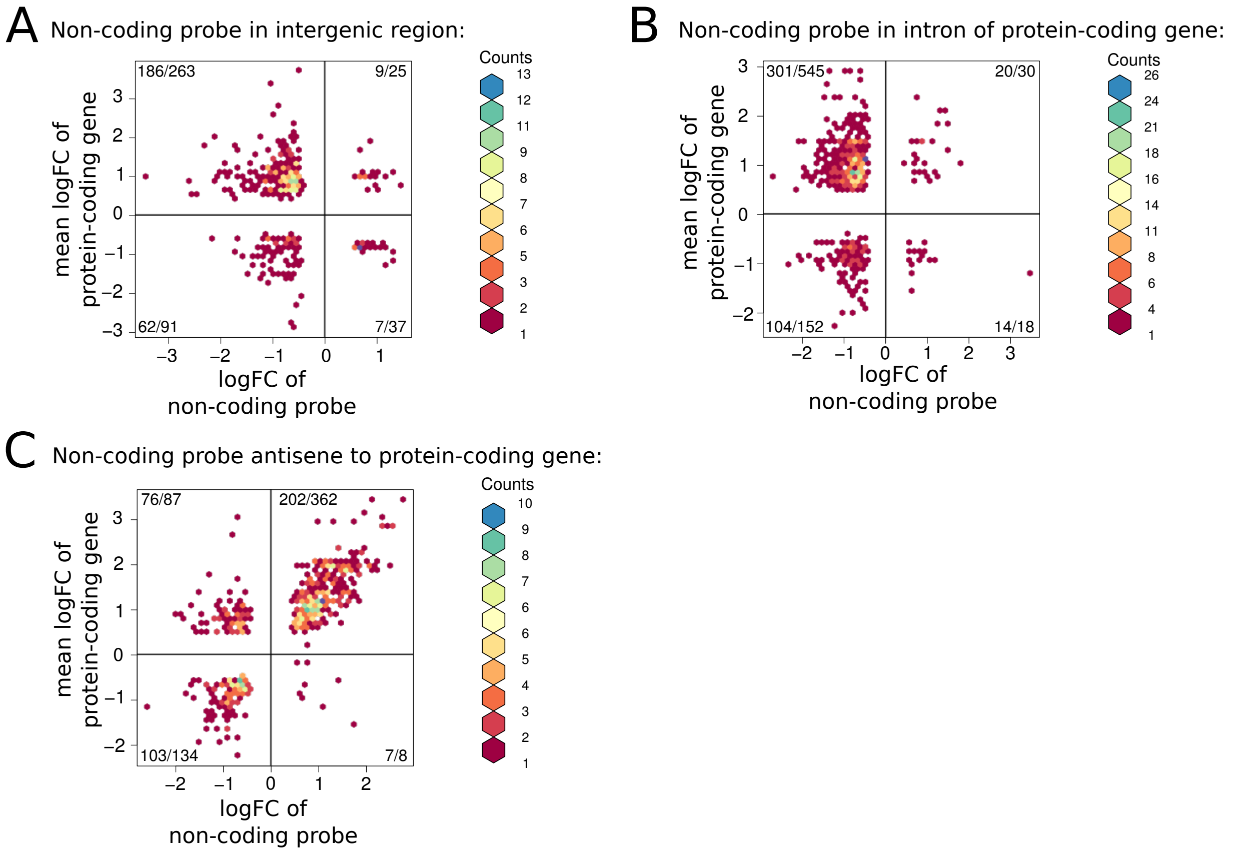

Figure S19: **Proximal ncRNA – mRNA pairs.** For *bona fide* non-coding probes significantly differentially expressed (FDR < 0.05) between astrocytoma of grade I and aggressive states (grade III or IV) the protein-coding gene (`Gencode` release v12) with closest genome coordinates was identified, and the pair retained if the protein-coding gene was differentially expressed at the same FDR. All pairs including a protein-coding gene with inconsistent probes, i.e. fold changes of significant probes mapping to exons of the gene exhibit opposite signs, were discarded. Log2 fold change of the *bona fide* non-coding probe (x-axis) and the average log2 fold change of protein-coding gene (y-axis) is depicted as a bivariate histogram using hexagonal binning (`R` package `hexbin`). Pairs with converse fold changes are shown in the left upper and right lower quadrant. Pairs with consistent fold changes but opposite reading direction are shown in the left lower and right upper quadrant. Numbers in quadrant correspond to number of unique genes/number of unique pairs depicted. (**A**) Proximal pairs, where the *bona fide* non-coding probe is intergenic. (**B**) Pairs where the *bona fide* non-coding probes is in an intron of the protein-coding gene. (**C**) Pairs where the *bona fide* non-coding probe and the protein-coding gene are on opposite strands and overlap at least partially.

Table S23: **Protein-coding genes proximal to *bona fide* non-coding DE-Probes and related to astrocytoma.** Several ncRNAs associated with different grades of glioma are transcribed from loci in the proximity of differentially expressed mRNAs with well known functions in glioma. Column heading **Location** denotes the genomic location of the *bona fide* non-coding DE-Probe relative to the astrocytoma related protein-coding gene (**mRNA**).

Location	mRNA	Role	Reference
intergenic	CTNNB1	A pivotal GBM oncogene.	[26]
	IGF1	Found to be associated with astrocytoma.	[27]
	KLF6	A tumor suppressor for astrocytoma.	[28]
	KLF9	Inhibitor of GBM-initiating stem cells.	[29]
	MET	A regulator of GBM stem cells.	[30]
	PTPRD	A tumor suppressor for astrocytoma.	[31]
	SMAD2	Negative prognostic factor and component of TGFβ.	[32]
	SULF2	A regulator of GBM cell growth.	[33]
intronic	CDH2	Found to promote glioblastoma cell migration upon cleavage by a proteinase.	[34]
	CDKN1A (p21)	The main target of p53 and suppressor of glioblastoma cell growth.	[35]
	GAB1	A component of EGFR signaling relevant in glioblastoma.	[36]
	HDAC1	A drug target in the disease.	[35]
	ITGA7	Frequently mutated in GBM.	[37]
	KLF6	A tumor suppressor for astrocytoma.	[28]
	KLF9	Inhibitor of GBM-initiating stem cells.	[29]
	LRP1	Promoter of GBM cell invasion.	[38]
	SOX6	A GBM antigen.	[39]
	WHSC1	A promoter of GBM proliferation.	[40]
antisense	BIRC5	A negative prognostic factor and drug target in GBM.	[41]
	CCND1	Associated with negative prognosis.	[42]
	CDKN1A (p21)	The main target of p53 and suppressor of glioblastoma cell growth.	[35]
	CST3	Involved in invasiveness.	[43]
	CTNNA1	Inhibiting migration, invasion and proliferation of glioma cells.	[44]
	EMC10 (HSS1)	An inhibitor of GBM cell growth.	[45]
	LGALS1	Involved in growth, invasion, and chemoresistance.	[46]
	LRP1	Promoter of GBM cell invasion.	[38]
	MARCKS	Involved in invasion.	[47]
	MKI67	Used as proliferation marker for GBM in histological grading (KI-67 labeling index).	[48]
	NTN1	An autocrine inhibitor of glioma cell motility.	[49]
	PDGFRA	Frequently amplified in astrocytoma.	[50, 51]
	PFKFB3	Overexpressed in high grades.	[52]
	PHF3	Is frequently downregulated or lost.	[53]
	SULF2	A regulator of GBM cell growth.	[33]

Table S24: **GO term enrichment for protein-coding genes proximal to *bona fide* non-coding DE-Probes in intergenic space.** Most enriched GO terms (P-value$< 5 \times 10^{-2}$, ontology Biological Process) of significantly differentially expressed genes (`Gencode` release 12) with a significantly differentially expressed *bona fide* non-coding probe in intergenic space (FDR $<$ 0.05). Column headings indicate ID of GO term (**ID**), significance of enrichment (**P-value**), odds ratios (**Odds ratio**), expected number of genes associated with tested GO term (**Exp. count**), number of significantly differentially expressed genes associated with this GO term (**Count**), number of genes from the gene universe that are annotated at this GO term (**Size**), and the GO term itself (**Term**). Analysis was done by using the Bioconductor `GOstats` package. Mapping of genes to GO terms is based on the `NCBI` gene information table (version: July 1, 2012). GO terms with evidence codes *IEA* were removed in order to discard automatically annotated relations. Significance of enrichment was assessed by a one-sided hypergeometric test where the universe contains all genes of the nONCOchip which passed unspecific filtering (Materials and Methods). Number of genes in universe: 6933. Number of genes in universe mapped to ontology: 4624. Number of selected genes: 257. Number of selected genes mapped to ontology and universe: 170.

ID	P-value	Odds ratio	Exp. count	Count	Size	Term
GO:0051239	3.031E-06	3.191	9.794	26	281	regulation of multicellular organismal process
GO:0051960	1.142E-05	4.347	4.118	15	112	regulation of nervous system development
GO:0006355	1.024E-04	1.987	30.221	50	822	regulation of transcription, DNA-dependent
GO:0045664	1.089E-04	4.986	2.392	10	66	regulation of neuron differentiation
GO:0060284	2.948E-04	3.709	3.727	12	103	regulation of cell development
GO:0010976	6.926E-04	13.392	0.441	4	12	positive regulation of neuron projection development
GO:0019219	7.818E-04	1.918	23.755	39	673	regulation of nucleobase-containing compound metabolic process
GO:0050773	7.980E-04	8.405	0.772	5	21	regulation of dendrite development
GO:0010628	9.356E-04	2.144	13.676	26	372	positive regulation of gene expression
GO:0019222	1.130E-03	1.766	36.312	53	1046	regulation of metabolic process
GO:0031344	1.402E-03	3.775	2.721	9	74	regulation of cell projection organization
GO:0048813	1.849E-03	6.718	0.919	5	25	dendrite morphogenesis
GO:0032501	1.971E-03	1.773	31.188	46	1006	multicellular organismal process
GO:0032774	2.113E-03	1.690	34.228	50	931	RNA biosynthetic process
GO:0060021	2.269E-03	8.920	0.588	4	16	palate development
GO:0048699	2.382E-03	2.077	12.316	23	335	generation of neurons
GO:0050769	2.648E-03	6.105	0.993	5	27	positive regulation of neurogenesis
GO:0050807	2.883E-03	8.232	0.625	4	17	regulation of synapse organization
GO:0021953	2.883E-03	8.232	0.625	4	17	central nervous system neuron differentiation
GO:0050770	3.670E-03	5.593	1.066	5	29	regulation of axonogenesis
GO:0022604	3.682E-03	2.997	3.713	10	101	regulation of cell morphogenesis
GO:0051130	4.123E-03	2.525	5.678	13	156	positive regulation of cellular component organization
GO:0051101	4.433E-03	7.131	0.699	4	19	regulation of DNA binding
GO:0003013	4.551E-03	3.387	2.647	8	72	circulatory system process

GO:0001501	4.598E-03	3.763	2.103	7	58	skeletal system development

Table S25: **GO term enrichment for protein-coding genes with *bona fide* non-coding DE-Probes in introns.** Most enriched GO terms (P-value$< 5 \times 10^{-2}$, ontology Biological Process) of significantly differentially expressed genes (`Gencode` release 12) with a significantly differentially expressed *bona fide* non-coding probe in intron (FDR < 0.05). Column headings indicate ID of GO term (**ID**), significance of enrichment (**P-value**), odds ratios (**Odds ratio**), expected number of genes associated with tested GO term (**Exp. count**), number of significantly differentially expressed genes associated with this GO term (**Count**), number of genes from the gene universe that are annotated at this GO term (**Size**), and the GO term itself (**Term**). Analysis was done by using the Bioconductor `GOstats` package. Mapping of genes to GO terms is based on the `NCBI` gene information table (version: July 1, 2012). GO terms with evidence codes *IEA* were removed in order to discard automatically annotated relations. Significance of enrichment was assessed by a one-sided hypergeometric test where the universe contains all genes of the nONCOchip which passed unspecific filtering (Materials and Methods). Number of genes in universe: 6933. Number of genes in universe mapped to ontology: 4624. Number of selected genes: 417. Number of selected genes mapped to ontology and universe: 292.

ID	P-value	Odds ratio	Exp. count	Count	Size	Term
GO:0010646	5.575E-05	1.999	28.164	49	446	regulation of cell communication
GO:0035113	6.480E-05	9.648	1.137	7	18	embryonic appendage morphogenesis
GO:0035108	3.793E-04	6.625	1.452	7	23	limb morphogenesis
GO:0030035	4.116E-04	8.241	1.074	6	17	microspike assembly
GO:0035637	8.154E-04	2.092	14.966	28	237	multicellular organismal signaling
GO:0065007	8.159E-04	1.507	175.301	201	2776	biological regulation
GO:0010718	8.196E-04	9.416	0.821	5	13	positive regulation of epithelial to mesenchymal transition
GO:0048736	8.635E-04	5.575	1.642	7	26	appendage development
GO:0023051	9.318E-04	1.658	39.973	59	633	regulation of signaling
GO:0070482	1.087E-03	3.711	3.221	10	51	response to oxygen levels
GO:0019219	1.355E-03	1.521	64.349	86	1019	regulation of nucleobase-containing compound metabolic process
GO:0071456	1.455E-03	6.038	1.326	6	21	cellular response to hypoxia
GO:0008624	1.690E-03	3.222	3.978	11	63	induction of apoptosis by extracellular signals
GO:0031644	1.951E-03	3.692	2.905	9	46	regulation of neurological system process
GO:0042733	2.414E-03	10.014	0.631	4	10	embryonic digit morphogenesis
GO:0045668	3.606E-03	8.581	0.695	4	11	negative regulation of osteoblast differentiation
GO:0009968	3.830E-03	2.054	11.269	21	180	negative regulation of signal transduction
GO:0012502	3.862E-03	2.093	10.546	20	167	induction of programmed cell death

Table S26: **GO term enrichment for protein-coding genes with antisense *bona fide* non-coding DE-Probes.** Most enriched GO terms (P-value$< 5 \times 10^{-2}$, ontology Biological Process) of significantly differentially expressed genes (Gencode release 12) with a significantly differentially expressed *bona fide* non-coding probe on the antisense strand (FDR < 0.05). Column headings indicate ID of GO term (**ID**), significance of enrichment (**P-value**), odds ratios (**Odds ratio**), expected number of genes associated with tested GO term (**Exp. count**), number of significantly differentially expressed genes associated with this GO term (**Count**), number of genes from the gene universe that are annotated at this GO term (**Size**), and the GO term itself (**Term**). Analysis was done by using the Bioconductor GOstats package. Mapping of genes to GO terms is based on the NCBI gene information table (version: July 1, 2012). GO terms with evidence codes *IEA* were removed in order to discard automatically annotated relations. Significance of enrichment was assessed by a one-sided hypergeometric test where the universe contains all genes of the nONCOchip which passed unspecific filtering (Materials and Methods). Number of genes in universe: 6933. Number of genes in universe mapped to ontology: 4624. Number of selected genes: 365. Number of selected genes mapped to ontology and universe: 263.

ID	P-value	Odds ratio	Exp. count	Count	Size	Term
GO:0060491	4.962E-05	9.910	1.081	7	19	regulation of cell projection assembly
GO:0051494	6.270E-05	7.569	1.479	8	26	negative regulation of cytoskeleton organization
GO:0031110	3.321E-04	8.461	1.024	6	18	regulation of microtubule polymerization or depolymerization
GO:0030203	4.625E-04	7.808	1.081	6	19	glycosaminoglycan metabolic process
GO:0030154	7.022E-04	1.723	35.437	54	629	cell differentiation
GO:0045664	7.085E-04	3.188	4.721	13	83	regulation of neuron differentiation
GO:0048015	8.966E-04	4.686	2.104	8	37	phosphatidylinositol-mediated signaling
GO:0030195	1.635E-03	11.210	0.569	4	10	negative regulation of blood coagulation
GO:0048731	2.461E-03	1.562	46.110	64	826	system development
GO:0050793	2.737E-03	1.750	23.320	37	410	regulation of developmental process
GO:0032886	3.022E-03	4.231	1.991	7	35	regulation of microtubule-based process
GO:0051130	3.045E-03	2.241	8.896	18	158	positive regulation of cellular component organization
GO:0046503	3.520E-03	8.403	0.683	4	12	glycerolipid catabolic process
GO:0006639	3.573E-03	4.085	2.048	7	36	acylglycerol metabolic process
GO:0031333	4.383E-03	5.615	1.138	5	20	negative regulation of protein complex assembly
GO:0032411	4.860E-03	7.468	0.739	4	13	positive regulation of transporter activity
GO:0009914	4.953E-03	3.118	3.299	9	58	hormone transport

7 Supplemental material and methods

7.1 Primers for RT-PCR and ChIP

Table S27: Primer and probe sequences used for PCR. All primers were synthesized by Eurofins MWG Operon (Ebersberg, Germany). Taqman probes were purchased from Roche Diagnostics (Mannheim, Germany) or Metabion (Martinsried, Germany), as indicated.

Assay	Forward	Reverse	Probe
Tissue distribution of STAiRs			
STAiR1	CTCAGTTTGGCATCCG-TTTT	ATTGACTTCCCAGGCC-TTTT	-
STAiR2	GTGAAGGGGCATGTTG-AGAT	GGTGCTAGCCCTGAAG-TCTG	-
STAiR18	GGAACACTCTGAAAAAC-ACCAA	TGAGAATACATATGT-GTGCAAGGA	-
GAPDH	AGCCACATCGCTCAGA-CAC	GCCCAATACGACCAA-ATCC	-
STAiR1 expression time course and ChIP			
GAPDH	AGCCACATCGCTCAGA-CAC	GCCCAATACGACCAA-ATCC	TGGGGAAG (#60 Roche Universal ProbeLibrary)
β-actin	TCGTGCGTGACATTAA-GGAGAA	AGCAGCCGTGGCCATCT	TACGTCGCCCTGGACTT-CGAGCA (Metabion)
STAiR1-P1	GCAGTCCCTTATACT-TACCATCAA	CTTACCACATTCGC-TGTAGATAGG	TCCTCTTCT (#35 Roche Universal ProbeLibrary)
STAiR1-P2	GGCACACACAGATTTTTA-CAGTG	TTGGATCCTCTTGACTTC-TGTCT	CAGCCTCC (#75 Roche Universal ProbeLibrary)
STAiR1-P3	CATGGTGGTACGTGCC-TGT	CCCCTACCTCATGGGTT-TAAG	GGAGGCTG (#75 Roche Universal ProbeLibrary)
STAiR1-P4	TACCATGATGTGACGAT-TCAGA	AGCCACCTCATGTACCC-AGA	GGAGGCAG (#16 Roche Universal ProbeLibrary)
STAiR1-P5	CTGGCCAGGGCAGAATTA	GCAAACAGGGACAATT-TGACT	CAGGAGAA (#2 Roche Universal ProbeLibrary)
STAiR1-P6	CAAACAATTTCTTGAAG-CGAT	GGGAGAACCAGGCTAT-TATGG	CAGGAGAA (#1 Roche Universal ProbeLibrary)

7.2 Annotation categories

For a complete annotation of known protein-coding genes we relied on RefSeq [54], UCSC [55], Ensembl [56], and Gencode v12 [57]. The first three datasets were downloaded from the University of California Santa Cruz (UCSC) table browser (hg19), while Gencode annotation were directly taken from http://www.gencodegenes.org/releases/12.html. For all sets the genomic coordinates of protein-coding genes, protein-coding transcript isoforms, and protein-coding exons (CDS) were used to define the coordinates of known protein-coding genes, transcript variants and exons, respectively. Intronic regions were defined by intervals annotated as an intron in at least one of the gene annotations sets above, but never annotated as an exon of a protein-coding transcript. Intergenic regions were defined as the complement of all protein-coding transcript variants known in at least one of the above annotation sets. For untranslated regions (UTRs) and pseudogenes we relied solely on the coordinates as defined in Gencode v12.

Annotation for known non-coding RNA genes has been collected from different sites: (1) A set of *bona fide* intergenic long non-coding RNAs was constructed from the 18855 transcripts defined in the long non-coding RNA dataset of Gencode v12. In order to exclude non-coding isoforms of protein-coding genes and antisense RNAs (which are not detectable by tiling arrays), we discarded all those transcripts that overlapped at least one known protein-coding transcript, no matter of reading direction (Gencode v12 - 7401 transcripts; UCSC, Ensembl, and RefSeq protein-coding genes - 8671). To further exclude transcripts predicted to contain conserved short open reading frames, we discarded all those transcripts with an exon that overlapped a significant RNAcode [58] segment (p-value< 0.05, 7500 transcripts), or if not scored by RNAcode, an exon that overlaps a significant tblastn hit (E-value< 0.05, RefSeq database from March 7, 2012; 8848 transcripts). The filtering steps resulted in 5209 long non-coding transcripts which corresponded to 3814 non-coding genes. (2) Large intergenic non-coding RNAs (lincRNAs) and transcripts of uncertain coding potential (TUCPs) as detected in a comprehensive expression study across 22 human tissues and cell lines have been downloaded from the Human Body Map catalog (http://www.broadinstitute.org/genome_bio/human_lincrnas/) [59]. (3) Genomic coordinates of large RNAs found in chromatin were taken from [60]. (4) Sequences of validated large non-coding RNAs were downloaded from the lncRNAdb database [61] and mapped to the human genome version hg19 by employing BLAT [62] with parameters -trimHardA -minIdentity=95. (5) Genomic coordinates of known short RNAs, like miRNAs and snoRNAs, were downloaded from the wgRNA track available from the UCSC table browser, and split in a subset containing the precursors of miRNAs and a subset of C/D box and H/ACA box snoRNAs as well as small Cajal body-specific RNAs (scaRNAs) [63, 64]. (6) Human intronic non-coding RNAs [65] were downloaded from the UCSC Genome Browser mirror for functional RNA (http://www.ncrna.org/glocal/cgi-bin/hgGateway) and mapped to hg19. Original sets of totally intronic non-coding RNAs (TINs) and partially intronic non-coding RNAs (PINs) were reannotated according to Gencode v12 gene annotation (no matter of reading direction) in order to receive reliable sets of intronic non-coding RNAs. 31023 TINs out of 55126 original TINs mapping to hg19 are completely found in introns and did not overlap with conserved open reading frames as detected by RNAcode (p-value< 0.05), or did not exhibit sequence similarity to known human amino acid sequences (tblastn, RefSeq database from March 7, 2012, E-value< 0.05) if RNAcode could not be applied due to low sequence conservation. 621 intronic non-coding RNAs classified as TINs in [65] overlapped Gencode v12 exons and were assigned to the set of partially intronic non-coding RNAs (PINs). 6268 PINs out of 12589 PINs

mapping to hg19 were partially found in introns and did not overlap with conserved short open reading frames detected in introns (RNAcode, p-value< 0.05). 141 intronic non-coding RNAs originally annotated as PINs did not overlap Gencode v12 exons and, hence, have been added to the set of totally intronic non-coding RNAs (TINs).

The number of DE-TAR segments with conserved secondary structure was retrieved by mapping their coordinates to genomic regions known to contain conserved secondary structure elements (Evofold [66], RNAz 2.0 [67, 68], and SISSIz [69]). For RNAz and SISSIz we relied on high scoring predictions from *Smith et al.* [70].

We retrieved genomic coordinates of selected histone modifications from the Encode consortium [71] in order to assess independent evidence for transcription initiation and elongation (including data for 6 normal cell lines, 1 cancer, and 1 embryonic stem cell line). To detect differential expression of known promoter-sites we relied on the histone modification H3K4 trimethylation, which marks promoter regions of actively transcribed genes [72, 73]. This chromatin mark often co-occurs with CpG islands, which are also associated with transcription start sites [74, 75]. In addition DNaseI-hypersensitive sites define regions where the chromatin structure is changed in a way such that transcription factor binding is possible [73, 76]. The genomic coordinates of transcription factor binding sites (TFBs) corresponded to binding sites identified by ChIP-seq [71] or found to be conserved within human/mouse/rat alignments [77]. PolII binding sites were also derived from Encode to assess the fraction of DE-TAR segments possibly transcribed by Polymerase II. A transcribed region of polII transcripts is marked by H3K36me3 [78], while transcriptional repression of a region is marked by H3k27me3 [79]. In contrast, H3K4me1 is associated with enhancer regions, but not with transcription start sites [80, 73], and H3K27Ac is associated with enhancer and promoter sites [71, 81, 82].

We used the R library genomeIntervals [6] to revise and adapt all annotation sets. A detailed listing of annotations sets and their sources is provided in Supplemental Table S28.

Table S28: **Detailed documentation of annotation categories used for enrichment analyses and general comparison of differentially expressed regions with known annotation sets.** Column headings **Annotation**, **Abbreviation**, **Source/URL**, **Assembly**, **Citation**, and **Comment** indicate the according genomic feature, the abbreviation used in figures and tables throughout the paper, the online source of the annotation data set, the human genome assembly for which annotation was available, references, and comments about required preprocessing of the annotation data, respectively.

Annotation	Abbreviation	Source/URL	Assembly	Citation	Comment
Protein-coding gene annotation					
Coding exons	CDS	`Gencode` v12	GRCh37/hg19	`Gencode` [57]	-
Introns	intron	`Gencode` v12 and `UCSC` table browser (tracks: `UCSC` genes, `RefSeq` genes, `Ensembl` genes)	GRCh37/hg19	`Gencode` [57], `Ensembl` [56], `RefSeq` [54], `UCSC` [55]; [83]	Defined as intronic nucleotides which do not overlap any exon of a protein-coding transcript.
Intergenic	intergenic	`Gencode` v12 and `UCSC` table browser (tracks: `UCSC` genes, `RefSeq` genes, `Ensembl` genes)	GRCh37/hg19	`Gencode` [57], `Ensembl` [56], `RefSeq` [54], `UCSC` [55]; [83]	Defined as the complement of all known protein-coding transcripts.
UTRs	UTRs	`Gencode` v12	GRCh37/hg19	`Gencode` [57]	-
Non-coding gene annotation					
Long non-coding RNAs	lncRNAs (`Gencode`)	`ftp://ftp.sanger.ac.uk/pub/gencode/release_12/gencode.v12.long_noncoding_RNAs.gtf.gz`	GRCh37/hg19	`Gencode` [57]	The original set of long non-coding RNAs as annotated in `Gencode` was reduced to a set of *bona fide* non-coding RNAs without any evidence for functional short ORFs (see descriptions above).
Large intergenic non-coding RNAs	lincRNAs	`http://www.broadinstitute.org/genome_bio/human_lincrnas/sites/default/files/lincRNA_catalog/lincRNAs_transcripts.bed`	GRCh37/hg19	[59]	-

Annotation	Abbreviation	Source/URL	Assembly	Citation	Comment
Transcripts of uncertain coding potential	TUCP	http://www.broadinstitute.org/genome_bio/human_lincrnas/sites/default/files/TUCP_transcripts_catalog/TUCP_transcripts.gtf	GRCh37/hg19	[59]	-
Chromatin associated RNAs	CARs	-	NCBI36/hg18	[60]	Mapped to GRCh37/hg19 using liftOver [83].
LncRNAdb	lncRNAdb	http://lncrnadb.com	-	[61]	Coordinates in GRCh37/hg19 have been derived by BLAT [62] with parameters -trimHardA -minIdentity=95.
Short RNAs	miRNAs, snoRNAs, scaRNAs	UCSC table browser (track: sno/miRNA)	GRCh37/hg19	[63, 64, 83]	-
Intronic non-coding RNAs	TINs, PINs	UCSC Genome Browser mirror for functional RNA (http://www.ncrna.org/glocal/cgi-bin/hgGateway)	NCBI36/hg18	[65]	Mapped to GRCh37/hg19 using liftOver [83]. The original set of human intronic non-coding RNAs [65] was reassessed according to gene annotation in hg19 (see descriptions above).
Regions of conserved secondary structure					
RNAz	RNAz	-	GRCh37/hg19	[70]	-
SISSIz	SISSIz	-	GRCh37/hg19	[70]	-
Evofold	Evofold	UCSC table browser (track: EvoFold)	GRCh37/hg19	[66, 83]	-
Regulation Tracks					
H3K4 trimethylation	H3K4me3	ftp://hgdownload.cse.ucsc.edu/goldenPath/hg18/encodeDCC/wgEncodeBroadChipSeq/	NCBI36/hg18	[71, 83]	Chromatin-mark associated with promoter sites [72, 73]. Mapped to GRCh37/hg19 using liftOver [83].
CpG islands	CpG	UCSC table browser (track: CpG Islands)	GRCh37/hg19	[84, 83]	Associated with transcription start sites [74, 75].
DNaseI-hypersensitive sites	DNaseI	UCSC table browser (track: DNaseI Clusters)	GRCh37/hg19	[71, 83]	Associated with transcription factor binding sites [73, 76].

Annotation	Abbreviation	Source/URL	Assembly	Citation	Comment
Transcription factor binding sites (TFBs)	TFBs (Encode)	UCSC table browser (track: Txn Factor ChIP)	GRCh37/hg19	[71, 83]	Binding sites identified by ChIP-seq [71].
Transcription factor binding sites (TFBs)	TFBs (Transfac)	UCSC table browser (track: TFBS Conserved)	GRCh37/hg19	[77, 83]	Binding sites conserved in human, mouse and rat from Transfac Matrix Database (v7.0) [77].
PolII binding sites	POL-II	ftp://hgdownload.cse.ucsc.edu/goldenPath/hg18/encodeDCC/wgEncodeBroadChipSeq/	NCBI36/hg18	[71, 83]	PolII binding sites derived by ChIP-seq [71]. Mapped to GRCh37/hg19 using liftOver [83].
H3K36 trimethylation	H3K36me3	ftp://hgdownload.cse.ucsc.edu/goldenPath/hg18/encodeDCC/wgEncodeBroadChipSeq/	NCBI36/hg18	[71, 83]	Chromatin-mark associated with active regions of PolII transcripts [78]. Mapped to GRCh37/hg19 using liftOver [83].
H3K27 trimethylation	H3K27me3	ftp://hgdownload.cse.ucsc.edu/goldenPath/hg18/encodeDCC/wgEncodeBroadChipSeq/	NCBI36/hg18	[71, 83]	Chromatin-mark associated with repressed regions of PolII transcripts [79]. Mapped to GRCh37/hg19 using liftOver [83].
H3K4 monomethylation	H3K4me1	ftp://hgdownload.cse.ucsc.edu/goldenPath/hg18/encodeDCC/wgEncodeBroadChipSeq/	NCBI36/hg18	[71, 83]	Chromatin-mark associated with enhancer regions [80, 73]. Mapped to GRCh37/hg19 using liftOver [83].
H3K27 acetylation	H3K27ac	ftp://hgdownload.cse.ucsc.edu/goldenPath/hg18/encodeDCC/wgEncodeBroadChipSeq/	NCBI36/hg18	[71, 83]	Chromatin-mark associated with enhancer and promoter sites [71, 81, 82].Mapped to GRCh37/hg19 using liftOver [83].
Other					
Repeats	-	UCSC table browser (track: RepeatMasker)	GRCh37/hg19	[85, 83]	-
Genome gaps	-	UCSC table browser (track: Gap)	GRCh37/hg19	[83]	-

References

[1] Böhlig L, Friedrich M, Engeland K: **p53 activates the PANK1/miRNA-107 gene leading to downregulation of CDK6 and p130 cell cycle proteins.** *Nucleic Acids Res* 2011, **39**(2):440–453, [http://dx.doi.org/10.1093/nar/gkq796].

[2] Sohr S, Engeland K: **The tumor suppressor p53 induces expression of the pregnancy-supporting human chorionic gonadotropin (hCG) CGB7 gene**. *Cell Cycle* 2011, **10**(21):3758–3767.

[3] Quaas M, Müller GA, Engeland K: **p53 can repress transcription of cell cycle genes through a p21(WAF1/CIP1)-dependent switch from MMB to DREAM protein complex binding at CHR promoter elements**. *Cell Cycle* 2012, **11**(24):4661–4672.

[4] Müller GA, Quaas M, Schumann M, Krause E, Padi M, Fischer M, Litovchick L, DeCaprio JA, Engeland K: **The CHR promoter element controls cell cycle-dependent gene transcription and binds the DREAM and MMB complexes**. *Nucleic Acids Res.* 2012, **40**(4):1561–1578.

[5] Otto C, Reiche K, Hackermüller J: **Detection of differentially expressed segments in tiling array data.** *Bioinformatics* 2012, **28**(11):1471–1479, [http://dx.doi.org/10.1093/bioinformatics/bts142].

[6] Gagneur J, Toedling J, Bourgon R, Delhomme N: *genomeIntervals: Operations on genomic intervals* 2012. [R package version 1.14.0].

[7] Ishii N, Ozaki K, Sato H, Mizuno H, Saito S, Takahashi A, Miyamoto Y, Ikegawa S, Kamatani N, Hori M, Saito S, Nakamura Y, Tanaka T: **Identification of a novel non-coding RNA, MIAT, that confers risk of myocardial infarction.** *J Hum Genet* 2006, **51**(12):1087–1099, [http://dx.doi.org/10.1007/s10038-006-0070-9].

[8] Eißmann M, Gutschner T, Hämmerle M, Günther S, Caudron-Herger M, Groß M, Schirmacher P, Rippe K, Braun T, Zörnig M, Diederichs S: **Loss of the abundant nuclear non-coding RNA MALAT1 is compatible with life and development.** *RNA Biol* 2012, **9**(8):1076–1087, [http://dx.doi.org/10.4161/rna.21089].

[9] Zhang B, Arun G, Mao YS, Lazar Z, Hung G, Bhattacharjee G, Xiao X, Booth CJ, Wu J, Zhang C, Spector DL: **The lncRNA Malat1 is dispensable for mouse development but its transcription plays a cis-regulatory role in the adult.** *Cell Rep* 2012, **2**:111–123, [http://dx.doi.org/10.1016/j.celrep.2012.06.003].

[10] Zhou Y, Zhang X, Klibanski A: **MEG3 noncoding RNA: a tumor suppressor.** *J Mol Endocrinol* 2012, **48**(3):R45–R53, [http://dx.doi.org/10.1530/JME-12-0008].

[11] Chun HK, Chung KS, Kim HC, Kang JE, Kang MA, Kim JT, Choi EH, Jung KE, Kim MH, Song EY, Kim SY, Won M, Lee HG: **OIP5 is a highly expressed potential therapeutic target for colorectal and gastric cancers.** *BMB Rep* 2010, **43**(5):349–354.

[12] Ulitsky I, Shkumatava A, Jan CH, Sive H, Bartel DP: **Conserved function of lincRNAs in vertebrate embryonic development despite rapid sequence evolution.** *Cell* 2011, **147**(7):1537–1550, [http://dx.doi.org/10.1016/j.cell.2011.11.055].

[13] Askarian-Amiri ME, Crawford J, French JD, Smart CE, Smith MA, Clark MB, Ru K, Mercer TR, Thompson ER, Lakhani SR, Vargas AC, Campbell IG, Brown MA, Dinger ME, Mattick JS: **SNORD-host RNA Zfas1 is a regulator of mammary development and a potential marker for breast cancer.** *RNA* 2011, **17**(5):878–891, [http://dx.doi.org/10.1261/rna.2528811].

[14] Zhang X, Lian Z, Padden C, Gerstein MB, Rozowsky J, Snyder M, Gingeras TR, Kapranov P, Weissman SM, Newburger PE: **A myelopoiesis-associated regulatory intergenic noncoding RNA transcript within the human HOXA cluster.** *Blood* 2009, **113**(11):2526–2534, [http://dx.doi.org/10.1182/blood-2008-06-162164].

[15] Wang KC, Yang YW, Liu B, Sanyal A, Corces-Zimmerman R, Chen Y, Lajoie BR, Protacio A, Flynn RA, Gupta RA, Wysocka J, Lei M, Dekker J, Helms JA, Chang HY: **A long noncoding RNA maintains active chromatin to coordinate homeotic gene expression.** *Nature* 2011, **472**(7341):120–124, [http://dx.doi.org/10.1038/nature09819].

[16] Williams GT, Mourtada-Maarabouni M, Farzaneh F: **A critical role for non-coding RNA GAS5 in growth arrest and rapamycin inhibition in human T-lymphocytes.** *Biochem Soc Trans* 2011, **39**(2):482–486, [http://dx.doi.org/10.1042/BST0390482].

[17] Mourtada-Maarabouni M, Pickard MR, Hedge VL, Farzaneh F, Williams GT: **GAS5, a non-protein-coding RNA, controls apoptosis and is downregulated in breast cancer.** *Oncogene* 2009, **28**(2):195–208, [http://dx.doi.org/10.1038/onc.2008.373].

[18] Vigneau S, Rohrlich PS, Brahic M, Bureau JF: **Tmevpg1, a candidate gene for the control of Theiler's virus persistence, could be implicated in the regulation of gamma interferon.** *J Virol* 2003, **77**(10):5632–5638.

[19] Collier SP, Collins PL, Williams CL, Boothby MR, Aune TM: **Cutting edge: influence of Tmevpg1, a long intergenic noncoding RNA, on the expression of Ifng by Th1 cells.** *J Immunol* 2012, **189**(5):2084–2088, [http://dx.doi.org/10.4049/jimmunol.1200774].

[20] Portales-Casamar E, Thongjuea S, Kwon AT, Arenillas D, Zhao X, Valen E, Yusuf D, Lenhard B, Wasserman WW, Sandelin A: **JASPAR 2010: the greatly expanded open-access database of transcription factor binding profiles.** *Nucleic Acids Res* 2010, **38**(Database issue):D105–D110, [http://dx.doi.org/10.1093/nar/gkp950].

[21] Chen X, Xu H, Yuan P, Fang F, Huss M, Vega VB, Wong E, Orlov YL, Zhang W, Jiang J, Loh YH, Yeo HC, Yeo ZX, Narang V, Govindarajan KR, Leong B, Shahab A, Ruan Y, Bourque G, Sung WK, Clarke ND, Wei CL, Ng HH: **Integration of external signaling pathways with the core transcriptional network in embryonic stem cells.** *Cell* 2008, **133**(6):1106–1117, [http://dx.doi.org/10.1016/j.cell.2008.04.043].

[22] Trimarchi T, Ntziachristos P, Aifantis I: **A new player SETs in myeloid malignancy.** *Nat Genet* 2013, **45**(8):846–847, [http://dx.doi.org/10.1038/ng.2709].

[23] Cristbal I, Garcia-Orti L, Cirauqui C, Cortes-Lavaud X, Garca-Snchez MA, Calasanz MJ, Odero MD: **Overexpression of SET is a recurrent event associated with poor outcome and contributes to protein phosphatase 2A inhibition in acute myeloid leukemia.** *Haematologica* 2012, **97**(4):543–550, [http://dx.doi.org/10.3324/haematol.2011.050542].

[24] Wong WSW, Nielsen R: **Detecting selection in noncoding regions of nucleotide sequences.** *Genetics* 2004, **167**(2):949–958, [http://dx.doi.org/10.1534/genetics.102.010959].

[25] Hasegawa M, Kishino H, Yano T: **Dating of the human-ape splitting by a molecular clock of mitochondrial DNA.** *J Mol Evol* 1985, **22**(2):160–174.

[26] Pu P, Zhang Z, Kang C, Jiang R, Jia Z, Wang G, Jiang H: **Downregulation of Wnt2 and beta-catenin by siRNA suppresses malignant glioma cell growth.** *Cancer Gene Ther* 2009, **16**(4):351–361, [http://dx.doi.org/10.1038/cgt.2008.78].

[27] Lönn S, Rothman N, Shapiro WR, Fine HA, Selker RG, Black PM, Loeffler JS, Hutchinson AA, Inskip PD: **Genetic variation in insulin-like growth factors and brain tumor risk.** *Neuro Oncol* 2008, **10**(4):553–559, [http://dx.doi.org/10.1215/15228517-2008-026].

[28] Kimmelman AC, Qiao RF, Narla G, Banno A, Lau N, Bos PD, Rodriguez NN, Liang BC, Guha A, Martignetti JA, Friedman SL, Chan AM: **Suppression of glioblastoma tumorigenicity by the Kruppel-like transcription factor KLF6.** *Oncogene* 2004, **23**(29):5077–5083, [http://dx.doi.org/10.1038/sj.onc.1207662].

[29] Ying M, Sang Y, Li Y, Guerrero-Cazares H, Quinones-Hinojosa A, Vescovi AL, Eberhart CG, Xia S, Laterra J: **Krüppel-like family of transcription factor 9, a differentiation-associated transcription factor, suppresses Notch1 signaling and inhibits glioblastoma-initiating stem cells.** *Stem Cells* 2011, **29**:20–31, [http://dx.doi.org/10.1002/stem.561].

[30] Joo KM, Jin J, Kim E, Kim KH, Kim Y, Kang BG, Kang YJ, Lathia JD, Cheong KH, Song PH, Kim H, Seol HJ, Kong DS, Lee JI, Rich JN, Lee J, Nam DH: **MET signaling regulates glioblastoma stem cells.** *Cancer Res* 2012, **72**(15):3828–3838, [http://dx.doi.org/10.1158/0008-5472.CAN-11-3760].

[31] Veeriah S, Brennan C, Meng S, Singh B, Fagin JA, Solit DB, Paty PB, Rohle D, Vivanco I, Chmielecki J, Pao W, Ladanyi M, Gerald WL, Liau L, Cloughesy TC, Mischel PS, Sander C, Taylor B, Schultz N, Major J, Heguy A, Fang F, Mellinghoff IK, Chan TA: **The tyrosine phosphatase PTPRD is a tumor suppressor that is frequently inactivated and mutated in glioblastoma and other human cancers.** *Proc Natl Acad Sci U S A* 2009, **106**(23):9435–9440, [http://dx.doi.org/10.1073/pnas.0900571106].

[32] Bruna A, Darken RS, Rojo F, Ocaña A, Peñuelas S, Arias A, Paris R, Tortosa A, Mora J, Baselga J, Seoane J: **High TGFbeta-Smad activity confers poor prognosis in glioma patients and promotes cell proliferation depending on the methylation of the PDGF-B gene.** *Cancer Cell* 2007, **11**(2):147–160, [http://dx.doi.org/10.1016/j.ccr.2006.11.023].

[33] Phillips JJ, Huillard E, Robinson AE, Ward A, Lum DH, Polley MY, Rosen SD, Rowitch DH, Werb Z: **Heparan sulfate sulfatase SULF2 regulates PDGFRα signaling and growth in human and mouse malignant glioma.** *J Clin Invest* 2012, **122**(3):911–922, [http://dx.doi.org/10.1172/JCI58215].

[34] Kohutek ZA, diPierro CG, Redpath GT, Hussaini IM: **ADAM-10-mediated N-cadherin cleavage is protein kinase C-alpha dependent and promotes glioblastoma cell migration.** *J Neurosci* 2009, **29**(14):4605–4615, [http://dx.doi.org/10.1523/JNEUROSCI.5126-08.2009].

[35] Bajbouj K, Mawrin C, Hartig R, Schulze-Luehrmann J, Wilisch-Neumann A, Roessner A, Schneider-Stock R: **P53-dependent antiproliferative and pro-apoptotic effects of trichostatin A (TSA) in glioblastoma cells.** *J Neurooncol* 2012, **107**(3):503–516, [http://dx.doi.org/10.1007/s11060-011-0791-2].

[36] Kapoor GS, Zhan Y, Johnson GR, O'Rourke DM: **Distinct domains in the SHP-2 phosphatase differentially regulate epidermal growth factor receptor/NF-kappaB activation through Gab1 in glioblastoma cells.** *Mol Cell Biol* 2004, **24**(2):823–836.

[37] Ren B, Yu YP, Tseng GC, Wu C, Chen K, Rao UN, Nelson J, Michalopoulos GK, Luo JH: **Analysis of integrin alpha7 mutations in prostate cancer, liver cancer, glioblastoma multiforme, and leiomyosarcoma.** *J Natl Cancer Inst* 2007, **99**(11):868–880, [http://dx.doi.org/10.1093/jnci/djk199].

[38] Song H, Li Y, Lee J, Schwartz AL, Bu G: **Low-density lipoprotein receptor-related protein 1 promotes cancer cell migration and invasion by inducing the expression of matrix metalloproteinases 2 and 9.** *Cancer Res* 2009, **69**(3):879–886, [http://dx.doi.org/10.1158/0008-5472.CAN-08-3379].

[39] Ueda R, Ohkusu-Tsukada K, Fusaki N, Soeda A, Kawase T, Kawakami Y, Toda M: **Identification of HLA-A2- and A24-restricted T-cell epitopes derived from SOX6 expressed in glioma stem cells for immunotherapy.** *Int J Cancer* 2010, **126**(4):919–929, [http://dx.doi.org/10.1002/ijc.24851].

[40] Li J, Yin C, Okamoto H, Mushlin H, Balgley BM, Lee CS, Yuan K, Ikejiri B, Glasker S, Vortmeyer AO, Oldfield EH, Weil RJ, Zhuang Z: **Identification of a novel proliferation-related protein, WHSC1 4a, in human gliomas.** *Neuro Oncol* 2008, **10**:45–51, [http://dx.doi.org/10.1215/15228517-2007-036].

[41] Chakravarti A, Noll E, Black PM, Finkelstein DF, Finkelstein DM, Dyson NJ, Loeffler JS: **Quantitatively determined survivin expression levels are of prognostic value in human gliomas.** *J Clin Oncol* 2002, **20**(4):1063–1068.

[42] Sallinen SL, Sallinen PK, Kononen JT, Syrjäkoski KM, Nupponen NN, Rantala IS, Helén PT, Helin HJ, Haapasalo HK: **Cyclin D1 expression in astrocytomas is associated with cell proliferation activity and patient prognosis.** *J Pathol* 1999, **188**(3):289–293, [http://dx.doi.org/3.0.CO;2-X].

[43] Konduri SD, Yanamandra N, Siddique K, Joseph A, Dinh DH, Olivero WC, Gujrati M, Kouraklis G, Swaroop A, Kyritsis AP, Rao JS: **Modulation of cystatin C expression impairs the invasive and tumorigenic potential of human glioblastoma cells.** *Oncogene* 2002, **21**(57):8705–8712, [http://dx.doi.org/10.1038/sj.onc.1205949].

[44] Ji H, Wang J, Fang B, Fang X, Lu Z: **α-Catenin inhibits glioma cell migration, invasion, and proliferation by suppression of β-catenin transactivation.** *J Neurooncol* 2011, **103**(3):445–451, [http://dx.doi.org/10.1007/s11060-010-0413-4].

[45] Junes-Gill KS, Gallaher TK, Gluzman-Poltorak Z, Miller JD, Wheeler CJ, Fan X, Basile LA: **hHSS1: a novel secreted factor and suppressor of glioma growth located at chromosome 19q13.33.** *J Neurooncol* 2011, **102**(2):197–211, [http://dx.doi.org/10.1007/s11060-010-0314-6].

[46] Mercier ML, Fortin S, Mathieu V, Kiss R, Lefranc F: **Galectins and gliomas.** *Brain Pathol* 2010, **20**:17–27, [http://dx.doi.org/10.1111/j.1750-3639.2009.00270.x].

[47] Micallef J, Taccone M, Mukherjee J, Croul S, Busby J, Moran MF, Guha A: **Epidermal growth factor receptor variant III-induced glioma invasion is mediated through myristoylated alanine-rich protein kinase C substrate overexpression.** *Cancer Res* 2009, **69**(19):7548–7556, [http://dx.doi.org/10.1158/0008-5472.CAN-08-4783].

[48] Shibata T, Burger PC, Kleihues P: **Ki-67 immunoperoxidase stain as marker for the histological grading of nervous system tumours.** *Acta Neurochir Suppl (Wien)* 1988, **43**:103–106.

[49] Jarjour AA, Durko M, Luk TL, Maral N, Shekarabi M, Kennedy TE: **Autocrine netrin function inhibits glioma cell motility and promotes focal adhesion formation.** *PLoS One* 2011, **6**(9):e25408, [http://dx.doi.org/10.1371/journal.pone.0025408].

[50] Hermanson M, Funa K, Hartman M, Claesson-Welsh L, Heldin CH, Westermark B, Nistr M: **Platelet-derived growth factor and its receptors in human glioma tissue: expression of messenger RNA and protein suggests the presence of autocrine and paracrine loops.** *Cancer Res* 1992, **52**(11):3213–3219.

[51] Fleming TP, Saxena A, Clark WC, Robertson JT, Oldfield EH, Aaronson SA, Ali IU: **Amplification and/or overexpression of platelet-derived growth factor receptors and epidermal growth factor receptor in human glial tumors.** *Cancer Res* 1992, **52**(16):4550–4553.

[52] Kessler R, Bleichert F, Warnke JP, Eschrich K: **6-Phosphofructo-2-kinase/fructose-2,6-bisphosphatase (PFKFB3) is up-regulated in high-grade astrocytomas.** *J Neurooncol* 2008, **86**(3):257–264, [http://dx.doi.org/10.1007/s11060-007-9471-7].

[53] Fischer U, Struss AK, Hemmer D, Michel A, Henn W, Steudel WI, Meese E: **PHF3 expression is frequently reduced in glioma.** *Cytogenet Cell Genet* 2001, **94**(3-4):131–136, [http://dx.doi.org/48804].

[54] Pruitt KD, Tatusova T, Maglott DR: **NCBI reference sequences (RefSeq): a curated non-redundant sequence database of genomes, transcripts and proteins.** *Nucleic Acids Res* 2007, **35**(Database issue):D61–D65, [http://dx.doi.org/10.1093/nar/gkl842].

[55] Meyer LR, Zweig AS, Hinrichs AS, Karolchik D, Kuhn RM, Wong M, Sloan CA, Rosenbloom KR, Roe G, Rhead B, Raney BJ, Pohl A, Malladi VS, Li CH, Lee BT, Learned K, Kirkup V, Hsu F, Heitner S, Harte RA, Haeussler M, Guruvadoo L, Goldman M, Giardine BM, Fujita PA, Dreszer TR, Diekhans M, Cline MS, Clawson H, Barber GP, Haussler D, Kent WJ: **The UCSC Genome Browser database: extensions and updates 2013.** *Nucleic Acids Res* 2012, [http://dx.doi.org/10.1093/nar/gks1048].

[56] Hubbard T, Barker D, Birney E, Cameron G, Chen Y, Clark L, Cox T, Cuff J, Curwen V, Down T, Durbin R, Eyras E, Gilbert J, Hammond M, Huminiecki L, Kasprzyk A, Lehvaslaiho H, Lijnzaad P, Melsopp C, Mongin E, Pettett R, Pocock M, Potter S, Rust A, Schmidt E, Searle S, Slater G, Smith J, Spooner W, Stabenau A, Stalker J, Stupka E, Ureta-Vidal A, Vastrik I, Clamp M: **The Ensembl genome database project.** *Nucleic Acids Res* 2002, **30**:38–41.

[57] Harrow J, Frankish A, Gonzalez JM, Tapanari E, Diekhans M, Kokocinski F, Aken BL, Barrell D, Zadissa A, Searle S, Barnes I, Bignell A, Boychenko V, Hunt T, Kay M, Mukherjee G, Rajan J, Despacio-Reyes G, Saunders G, Steward C, Harte R, Lin M, Howald C, Tanzer A, Derrien T, Chrast J, Walters N, Balasubramanian S, Pei B, Tress M, Rodriguez JM, Ezkurdia I, van Baren J, Brent M, Haussler D, Kellis M, Valencia A, Reymond A, Gerstein M, Guigó R, Hubbard TJ: **GENCODE: the reference human genome annotation for The ENCODE Project.** *Genome Res* 2012, **22**(9):1760–1774, [http://dx.doi.org/10.1101/gr.135350.111].

[58] Washietl S, Findeiß S, Müller SA, Kalkhof S, von Bergen M, Hofacker IL, Stadler PF, Goldman N: **RNAcode: robust discrimination of coding and noncoding regions in comparative sequence data.** *RNA* 2011, **17**(4):578–594, [http://dx.doi.org/10.1261/rna.2536111].

[59] Cabili MN, Trapnell C, Goff L, Koziol M, Tazon-Vega B, Regev A, Rinn JL: **Integrative annotation of human large intergenic noncoding RNAs reveals global properties and specific subclasses.** *Genes Dev* 2011, **25**(18):1915–1927, [http://dx.doi.org/10.1101/gad.17446611].

[60] Mondal T, Rasmussen M, Pandey GK, Isaksson A, Kanduri C: **Characterization of the RNA content of chromatin.** *Genome Res* 2010, **20**(7):899–907, [http://dx.doi.org/10.1101/gr.103473.109].

[61] Amaral PP, Clark MB, Gascoigne DK, Dinger ME, Mattick JS: **lncRNAdb: a reference database for long noncoding RNAs.** *Nucleic Acids Res* 2011, **39**(Database issue):D146–D151, [http://dx.doi.org/10.1093/nar/gkq1138].

[62] Kent WJ: **BLAT–the BLAST-like alignment tool.** *Genome Res* 2002, **12**(4):656–664, [http://dx.doi.org/10.1101/gr.229202.ArticlepublishedonlinebeforeMarch2002].

[63] Lestrade L, Weber MJ: **snoRNA-LBME-db, a comprehensive database of human H/ACA and C/D box snoRNAs.** *Nucleic Acids Res* 2006, **34**(Database issue):D158–D162, [http://dx.doi.org/10.1093/nar/gkj002].

[64] Griffiths-Jones S: **The microRNA Registry.** *Nucleic Acids Res* 2004, **32**(Database issue):D109–D111, [http://dx.doi.org/10.1093/nar/gkh023].

[65] Nakaya HI, Amaral PP, Louro R, Lopes A, Fachel AA, Moreira YB, El-Jundi TA, da Silva AM, Reis EM, Verjovski-Almeida S: **Genome mapping and expression analyses of human intronic noncoding RNAs reveal tissue-specific patterns and enrichment in genes related to regulation of transcription.** *Genome Biol* 2007, **8**(3):R43, [http://dx.doi.org/10.1186/gb-2007-8-3-r43].

[66] Pedersen JS, Bejerano G, Siepel A, Rosenbloom K, Lindblad-Toh K, Lander ES, Kent J, Miller W, Haussler D: **Identification and classification of conserved RNA secondary structures in the human genome.** *PLoS Comput Biol* 2006, **2**(4):e33.

[67] Washietl S, Hofacker IL, Stadler PF: **Fast and reliable prediction of noncoding RNAs.** *Proc Natl Acad Sci U S A* 2005, **102**(7):2454–2459.

[68] Gruber A, Findeiß S, Washietl S, Hofacker I, Stadler P: **RNAz 2.0: Improved noncoding RNA detection**. In *PSB in press* 2010.

[69] Gesell T, Washietl S: **Dinucleotide controlled null models for comparative RNA gene prediction.** *BMC Bioinformatics* 2008, **9**:248.

[70] Smith MA, Gesell T, Stadler PF, Mattick JS: **Widespread purifying selection on RNA structure in mammals.** *Nucleic Acids Res* 2013, [http://dx.doi.org/10.1093/nar/gkt596].

[71] The ENCODE Project Consortium: **Identification and analysis of functional elements in 1% of the human genome by the ENCODE pilot project.** *Nature* 2007, **447**(7146):799–816.

[72] Roh TY, Cuddapah S, Cui K, Zhao K: **The genomic landscape of histone modifications in human T cells.** *Proc Natl Acad Sci U S A* 2006, **103**(43):15782–15787, [http://dx.doi.org/10.1073/pnas.0607617103].

[73] Bulger M, Groudine M: **Enhancers: the abundance and function of regulatory sequences beyond promoters.** *Dev Biol* 2010, **339**(2):250–257, [http://dx.doi.org/10.1016/j.ydbio.2009.11.035].

[74] Deaton AM, Bird A: **CpG islands and the regulation of transcription.** *Genes Dev* 2011, **25**(10):1010–1022, [http://dx.doi.org/10.1101/gad.2037511].

[75] Guenther MG, Levine SS, Boyer LA, Jaenisch R, Young RA: **A chromatin landmark and transcription initiation at most promoters in human cells.** *Cell* 2007, **130**:77–88, [http://dx.doi.org/10.1016/j.cell.2007.05.042].

[76] Xi H, Shulha HP, Lin JM, Vales TR, Fu Y, Bodine DM, McKay RDG, Chenoweth JG, Tesar PJ, Furey TS, Ren B, Weng Z, Crawford GE: **Identification and characterization of cell type-specific and ubiquitous chromatin regulatory structures in the human genome.** *PLoS Genet* 2007, **3**(8):e136, [http://dx.doi.org/10.1371/journal.pgen.0030136].

[77] Wingender E, Chen X, Hehl R, Karas H, Liebich I, Matys V, Meinhardt T, Prüss M, Reuter I, Schacherer F: **TRANSFAC: an integrated system for gene expression regulation.** *Nucleic Acids Res* 2000, **28**:316–319.

[78] Mikkelsen TS, Ku M, Jaffe DB, Issac B, Lieberman E, Giannoukos G, Alvarez P, Brockman W, Kim TK, Koche RP, Lee W, Mendenhall E, O'Donovan A, Presser A, Russ C, Xie X, Meissner A, Wernig M, Jaenisch R, Nusbaum C, Lander ES, Bernstein BE: **Genome-wide maps of chromatin state in pluripotent and lineage-committed cells.** *Nature* 2007, **448**(7153):553–560, [http://dx.doi.org/10.1038/nature06008].

[79] Barski A, Cuddapah S, Cui K, Roh TY, Schones DE, Wang Z, Wei G, Chepelev I, Zhao K: **High-resolution profiling of histone methylations in the human genome.** *Cell* 2007, **129**(4):823–837, [http://dx.doi.org/10.1016/j.cell.2007.05.009].

[80] Heintzman ND, Stuart RK, Hon G, Fu Y, Ching CW, Hawkins RD, Barrera LO, Calcar SV, Qu C, Ching KA, Wang W, Weng Z, Green RD, Crawford GE, Ren B: **Distinct and predictive chromatin signatures of transcriptional promoters and enhancers in the human genome.** *Nat Genet* 2007, **39**(3):311–318, [http://dx.doi.org/10.1038/ng1966].

[81] Terrenoire E, McRonald F, Halsall JA, Page P, Illingworth RS, Taylor AMR, Davison V, O'Neill LP, Turner BM: **Immunostaining of modified histones defines high-level features of the human metaphase epigenome.** *Genome Biol* 2010, **11**(11):R110, [http://dx.doi.org/10.1186/gb-2010-11-11-r110].

[82] Shin JH, Li RW, Gao Y, Baldwin R 6th, Li Cj: **Genome-wide ChIP-seq mapping and analysis reveal butyrate-induced acetylation of H3K9 and H3K27 correlated with transcription activity in bovine cells.** *Funct Integr Genomics* 2012, **12**:119–130, [http://dx.doi.org/10.1007/s10142-012-0263-6].

[83] Kuhn RM, Haussler D, Kent WJ: **The UCSC genome browser and associated tools**. *Brief. Bioinformatics* 2012.

[84] Gardiner-Garden M, Frommer M: **CpG islands in vertebrate genomes**. *J. Mol. Biol.* 1987, **196**(2):261–282.

[85] Smit A, Hubley R, Green P: *RepeatMasker Open-3.0* 2010, [http://www.repeatmasker.org].
