# Supplementary material for: Cell cycle, oncogenic and tumor suppressor pathways regulate numerous long and macro non-protein-coding RNAs
Source: Genome Biol. 2014 Mar 4;15(3):R48. doi: 10.1186/gb-2014-15-3-r48 (PMC4054595; doi:10.1186/gb-2014-15-3-r48)
Supplement: Additional file 2 — Online supplemental material containing BED formatted files of differentially expressed regions and macroRNAs, as well as CSV tables listing all proximal ncRNA-mRNA pairs regulated in astrocytoma. [file gb-2014-15-3-r48-S2.zip › online_data/index.html]

**Differentially expressed TARs in hg19 (DE-TARs)**

1. Cell cycle regulated
   - G0 versus G1 [BED:all] [BED:*bona fide* non-coding DE-TARs]
   - G1 versus S [BED:all] [BED:*bona fide* non-coding DE-TARs]
   - S versus G2 [BED:all] [BED:*bona fide* non-coding DE-TARs]
   - G0 versus G2 [BED:all] [BED:*bona fide* non-coding DE-TARs]
2. P53 regulated
   - D53wt cells with defunct endogenous P53 versus 53wt cells stably transfected with P53 wild type [BED:all] [BED:*bona fide* non-coding DE-TARs]
3. Stat-3 regulated
   - INA-6 cells deprived from IL-6 for 13h versus INA-6 cells after one hour of restimulation [BED:all] [BED:*bona fide* non-coding DE-TARs]
   - INA-6 cells deprived from IL-6 for 13h versus INA-6 cells permanently cultured in presence of IL-6 [BED:all] [BED:*bona fide* non-coding DE-TARs]
   - INA-6 cells restimulated for one hour versus INA-6 cells permanently cultured in presence of IL-6 [BED:all] [BED:*bona fide* non-coding DE-TARs]

**Transcriptionally active macroRNAs in hg19**

1. Expressed within cell cycle (HFF cells):
   - G0 (resting phase) [BED]
   - G1 phase [BED]
   - S phase [BED]
   - G2 phase [BED]
2. Expressed in D53wt cells:
   - D53wt cells with defunct endogenous P53 [BED]
   - D53wt cells stably transfected with tetracycline responsive wild type P53 [BED]
3. Expressed in INA-6 cells:
   - INA-6 cells deprived from IL-6 for 13h [BED]
   - INA-6 cells after one hour of restimulation [BED]
   - INA-6 cells permanently cultured in presence of IL-6 [BED]

**Differentially expressed macro RNAs in hg19**

1. Cell cycle regulated
   - G0 versus G1 [BED:up-regulated] [BED:down-regulated]
   - G1 versus S [BED:up-regulated] [BED:down-regulated]
   - S versus G2 [BED:up-regulated] [BED:down-regulated]
   - G0 versus G2 [BED:up-regulated] [BED:down-regulated]
2. P53 regulated
   - D53wt cells with defunct endogenous P53 versus 53wt cells stably transfected with P53 wild type [BED:up-regulated] [BED:down-regulated]
3. Stat-3 regulated
   - INA-6 cells deprived from IL-6 for 13h versus INA-6 cells after one hour of restimulation [BED:up-regulated] [BED:down-regulated]
   - INA-6 cells deprived from IL-6 for 13h versus INA-6 cells permanently cultured in presence of IL-6 [BED:up-regulated] [BED:down-regulated]
   - INA-6 cells restimulated for one hour versus INA-6 cells permanently cultured in presence of IL-6 [BED:up-regulated] [BED:down-regulated]

**Pairs of ncRNAs and closest mRNA**
  
  
Pairs of bona fide non-coding probes and protein-coding genes (Gencode v12) with closest genome coordinates and both significantly differentially expressed between clinical samples of astrocytoma of grade I versus samples of astrocytoma of aggressive grades (grades III or IV, FDR<0.05).
A fold change larger than 0 denotes high expression in tissue samples of grade I and low expression in samples of aggressive grades, i.e. logFC < 0 ≡ Grade I < Aggressive. A fold change less than 0 denotes low expression in grade I and high expression in aggressive grades, i.e. logFC > 0 ≡ Grade I > Aggressive.

1. *Bona fide* non-coding probe in intergenic space [CSV]- *Bona fide* non-coding probe in intronic space [CSV]- *Bona fide* non-coding probe antisense to protein-coding gene [CSV]

**R tool *stairFinder*** [R]
